# Genetic and functional analyses of *CTBP2* in anorexia nervosa and body weight regulation

**DOI:** 10.1038/s41380-024-02791-3

**Published:** 2024-11-07

**Authors:** Johanna Giuranna, Yiran Zheng, Matthäus Brandt, Sigrid Jall, Amrita Mukherjee, Soni Shankhwar, Simone Renner, Nirup Kumar Kurapati, Caroline May, Triinu Peters, Beate Herpertz-Dahlmann, Jochen Seitz, Martina de Zwaan, Wolfgang Herzog, Stefan Ehrlich, Stephan Zipfel, Katrin Giel, Karin Egberts, Roland Burghardt, Manuel Föcker, Katrin Marcus, Kathy Keyvani, Timo D. Müller, Frank Schmitz, Luisa Sophie Rajcsanyi, Anke Hinney

**Affiliations:** 1 Department of Child and Adolescent Psychiatry, Psychosomatics and Psychotherapy, University Hospital Essen, University of Duisburg-Essen, Essen, Germany; 2 Center for Translational Neuro- and Behavioral Sciences, University Hospital Essen, Essen, Germany; 3 Lead Discovery Center GmbH, Dortmund, Germany; 4 Institute for Diabetes and Obesity, Helmholtz Diabetes Center at Helmholtz Zentrum München, German Research Center for Environmental Health (GmbH), Neuherberg, Germany; 5 German Center for Diabetes Research (DZD), Neuherberg, Germany; 6 Department of Neuroanatomy, Institute of Anatomy and Cell Biology, Medical School, Saarland University, Homburg, Germany; 7 Institute of Molecular Animal Breeding and Biotechnology, Ludwig-Maximilian University Munich (LMU), Munich, Germany; 8 Institute of Neuropathology, University Hospital Essen, University of Duisburg-Essen, Essen, Germany; 9 Medizinisches Proteom-Center, Ruhr-University Bochum, Bochum, Germany; 10 Section of Molecular Genetics in Mental Disorders, University Hospital Essen, Essen, Germany; 11 Institute of Sex and Gender-Sensitive Medicine, University Hospital Essen, Essen, Germany; 12 Department of Child and Adolescent Psychiatry and Psychotherapy, University Hospital of the RWTH Aachen, Aachen, Germany; 13 Department of Psychosomatic Medicine and Psychotherapy, Hannover Medical School, Hannover, Germany; 14 Department of Internal Medicine II, General Internal and Psychosomatic Medicine, University of Heidelberg, Heidelberg, Germany; 15 Eating Disorders Research and Treatment Center, Department of Child and Adolescent Psychiatry, Faculty of Medicine, TU Dresden, Dresden, Germany; 16 Translational Developmental Neuroscience Section, Division of Psychological and Social Medicine and Developmental Neurosciences, Faculty of Medicine, TU Dresden, Germany; 17 Department of Psychosomatic Medicine and Psychotherapy, Medical University Hospital Tübingen, Tübingen, Germany; 18 Center of Excellence in Eating Disorders KOMET, Tübingen, Germany; 19 German Center for Mental Health (DZPG), Tübingen, Germany; 20 Department of Child and Adolescent Psychiatry, Psychosomatics and Psychotherapy, University of Würzburg, Würzburg, Germany; 21 Child and Adolescent Psychiatry Clinic, Oberberg Fachklinik Fasanenkiez Berlin, Berlin, Germany; 22 Department of Child and Adolescent Psychiatry, Psychosomatics and Psychotherapy, University Hospital Münster, Münster, Germany; 23 LWL-University Hospital Hamm for Child and Adolescent Psychiatry, Ruhr-University Bochum, Hamm, Germany; 24 Walther-Straub-Insitute for Pharmacology and Toxicology, Ludwig-Maximilians University Munich (LMU), Munich, Germany; 25 Present Address: Helmholtz Pioneer Campus, Helmholtz Zentrum München, Munich, Germany

**Keywords:** Genetics, Psychiatric disorders

## Abstract

The C-terminal binding protein 2 (*CTBP2*) gene (translational isoforms: CTBP2-L/S, RIBEYE) had been identified by a cross-trait analysis of genome-wide association studies for anorexia nervosa (AN) and body mass index (BMI). Here, we did a mutation analysis in *CTBP2* by performing polymerase chain reactions with subsequent Sanger-sequencing to identify variants relevant for AN and body weight regulation and ensued functional studies. Analysis of the coding regions of *CTBP2* in 462 female patients with AN (acute or recovered), 490 children and adolescents with severe obesity, 445 healthy-lean adult individuals and 168 healthy adult individuals with normal body weight detected 24 variants located in the specific exon of RIBEYE. In the initial analysis, three of these were rare non-synonymous variants (NSVs) detected heterozygously in patients with AN (p.Arg72Trp - rs146900874; p.Val289Met -rs375685611 and p.Gly362Arg - rs202010294). Four NSVs and one heterozygous frameshift variant were exclusively detected in children and adolescents with severe obesity (p.Pro53Ser - rs150867595; p.Gln175Arg*fs*Ter45 - rs141864737; p.Leu310Val - rs769811964; p.Pro397Ala - rs76134089 and p.Pro402Ser - rs113477585). *Ribeye* mRNA was detected in mouse hypothalamus. No effect of fasting or overfeeding on murine hypothalamic *Ribeye* expression was determined. Yet, increased *Ribeye* expression was detected in hypothalami of leptin-treated *Lep*^*ob/ob*^ mice. This increase was not related to reduced food intake and leptin-induced weight loss. We detected rare and frequent variants in the *RIBEYE* specific exon in both patients with AN and in children and adolescents with severe obesity. Our data suggest *RIBEYE* as a relevant gene for weight regulation.

## Introduction

Anorexia nervosa (AN) is a severe eating disorder characterized by an extremely low body weight, food restriction, body image disturbance and fear of gaining weight [1–3]. AN typically begins in adolescence and affects females more frequently than males (ratio at least 13:1; [4]). Further, it is the psychiatric disorder with the highest morbidity and mortality [5, 6].

Genome-wide association studies (GWAS) led to the identification of genes that contribute to complex phenotypes [7]. Previously, we have performed a look-up analysis of the 1.000 single nucleotide polymorphisms (SNPs) with the lowest *p*-values from a GWAS for AN [8] to identify associations in the at that time largest published GWAS for BMI variation [7]. Hereby, nine study-wide significant SNPs (*p-*value < 5 × 10^−5^) at three independent loci (chr2: calcium responsive transcription factor gene (*CARF*) and neurobeachin like 1 gene (*NBEAL1*), chr10: C-terminal binding protein 2 gene (*CTBP2*) and chr19: cyclin E1 gene (*CCNE1*)) were identified [9]. All AN-susceptibility alleles were associated with a decreased BMI. Three of these SNPs (rs1561589, rs12771627, rs11245456) with the lowest *p*-values are located in the *CTBP2* gene. The reported BMI associations mainly derived from females (lowest nominal *p*_females_ = 3.45 × 10^−7^) [9]. All nine SNPs were found to be genome-wide significant in the latest GWAS for BMI [10].

CTBP2 is part of the CTBP protein family that bind to the C-terminus of the adenovirus E1A [11]. CTBP1 and CTBP2 are highly homologous to NAD^+^-dependent dehydrogenases through their central domain [12] which plays an important role in the oligomerization of CTBPs [12–14]. Homo- and heterodimers of CTBP1 and CTBP2 can be formed [12]. CTBPs recognize the PXDLS motif (Pro-X-Asp-Leu-Ser) of DNA-binding proteins [15]. The C-terminus of Forkhead box O1 (FoxO1), a transcription factor that can regulate adipocyte differentiation, can directly interact with CTBP2 in mice [16]. The CTBP2/FOXO1 complex, formed by increased concentrations of NADH, can regulate gluconeogenesis responding to metabolic alterations in liver [16].

**Fig. 1:**
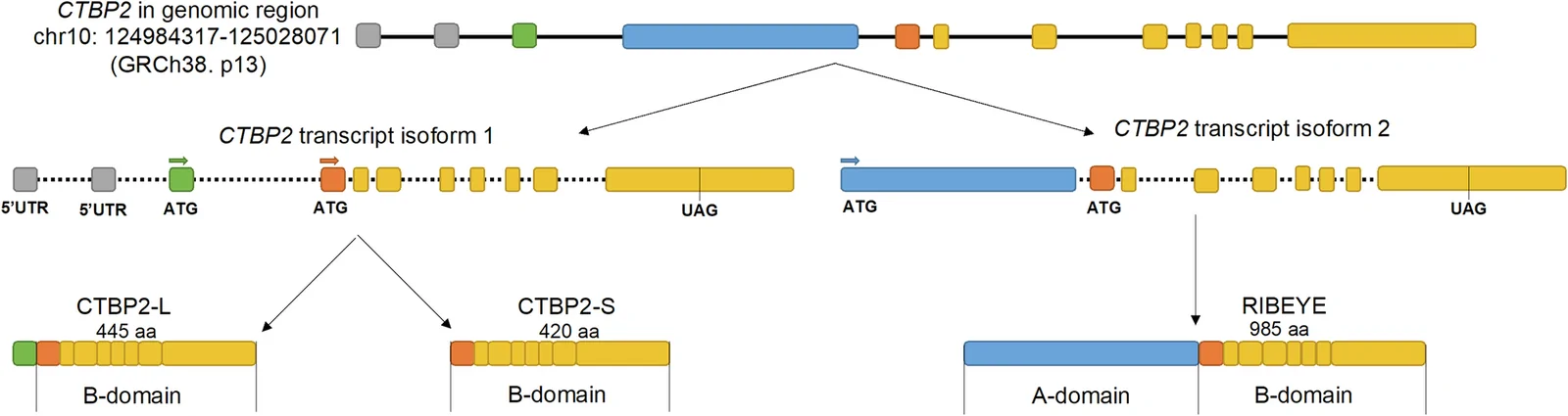
Schematic representation (not to scale) of the exon-intron structure of the *CTBP2* genomic locus and splicing pattern. The green boxes represent exons exclusively producing CTBP2-L. The orange boxes are exons specific for CTBP2-S, while the blue boxes indicate RIBEYE’s specific A-domain [17].

To date, three isoforms of *CTBP2* are known, namely CTBP2-L, CTBP2-S [17], and RIBEYE [18]. The two isoforms CTBP2-L and CTBP2-S are translated from the same transcript, while RIBEYE is formed by differential promoter usage (Fig. 1). The upstream promoter generates CTBP2-L/S, a transcriptional co-repressor that associates with other repressors and histone modifying enzymes, including class I histone deacetylases HDAC1/2, histone methyltransferases (HMTs, G9a) histone-lysine-specific demethylase (LSD1) and polycomb proteins to turn off target genes [19, 20]. CTBP2-S is 25 amino acids shorter (N-terminal nuclear localization signal, NLS) than CTBP2-L and has a cytosolic localization, while CTBP2-L has a predominant nuclear localization [17, 18]. All three isoforms contain the full-length C-terminal B-domain (420 aa), whereas the N-terminal A-domain (565 aa) is specific for RIBEYE (Fig. 1). A different promoter produces RIBEYE, a component of synaptic ribbons that possesses many primed vesicles undergoing Ca^2+^ stimulated exocytosis at high rates [21]. CTBP2 and RIBEYE B-domain bind NAD^+^ with high affinity. CTBP2, that forms RIBEYE B-domain (except for CtBP2-specific N-terminal sequences) is expressed in all tissues, while RIBEYE is only detectable in synaptic ribbons as found in the retina [18]. Three binding sites in the A-domain and two binding sites in the B-domain mediate the multiple RIBEYE-RIBEYE interactions to generate the scaffold of the synaptic ribbon [22]. RIBEYE knockout mice (deleted A-domain genomic region) fully lose the presynaptic ribbons in retinal synapses [18]. All *CTBP2*-derived proteins are differently regulated. While CTBP2-L/S is ubiquitously expressed, RIBEYE is expressed only where synaptic ribbons are found, e.g., at photoreceptors and bipolar cells in the retina, hair cells in the inner ear and pinealocytes of the pineal gland [18, 21, 23, 24].

As the hypothalamus is the major regulator of appetite and energy homeostasis [25], we previously have investigated the effects of fasting and re-feeding on the hypothalamic expression of genes associated with an increased risk for AN and a decreased BMI (*Ctbp2* and *Nbeal1*) [9]. Thus, functional *ex*-*vivo* studies in mice were conducted revealing that fasting led to a reduced hypothalamic expression of *Ctbp2* (both transcriptional isoforms) and *Nbeal1*, while the expression of *Ctbp2* in diet-induced obese (DIO) mice was upregulated in comparison to age-matched lean controls [9].

Previous studies already indicated a putative involvement of *CTBP2* in weight regulation. In fact, CTBP2-L/S together with CTBP1/BARS regulate the brown adipogenic program by building a complex with the proline-rich (PR) domain-containing protein 16 (PRDM16) and repressing the expression of white adipose tissue genes like resistin [26, 27]. Further, one recent study revealed that the proteins of *Ctbp2* are inactive in obesity and showed a relevance between its inactivation and pathogenesis of obesity-related metabolic disturbances [16]. However, this study did not consider different isoforms. A recent study provided further evidence for *CTBP2’s* role in obesity. It was reported that CTBP2’s protein expression was reduced in pancreatic islets cells in various mouse models, but also in human obesity. Further, CTBP2 stimulates the gene expression of insulin by altering histone modifications directly impacting the chromatin architecture. A depletion of CTBP2 affected insulin secretion and led to a glucose intolerance [28]. Yet, the impact of RIBEYE on the molecular physiology of body weight regulation has not been confirmed so far. Nevertheless, few studies showed a functional interaction of the brain derived neurotrophic factor (BDNF) and RIBEYE [29–31]. BDNF is known to influence the development of both, AN and obesity [7, 32–34]. A previous study even suggested that the variation of BDNF might be a state marker for AN [35].

**Fig. 2:**
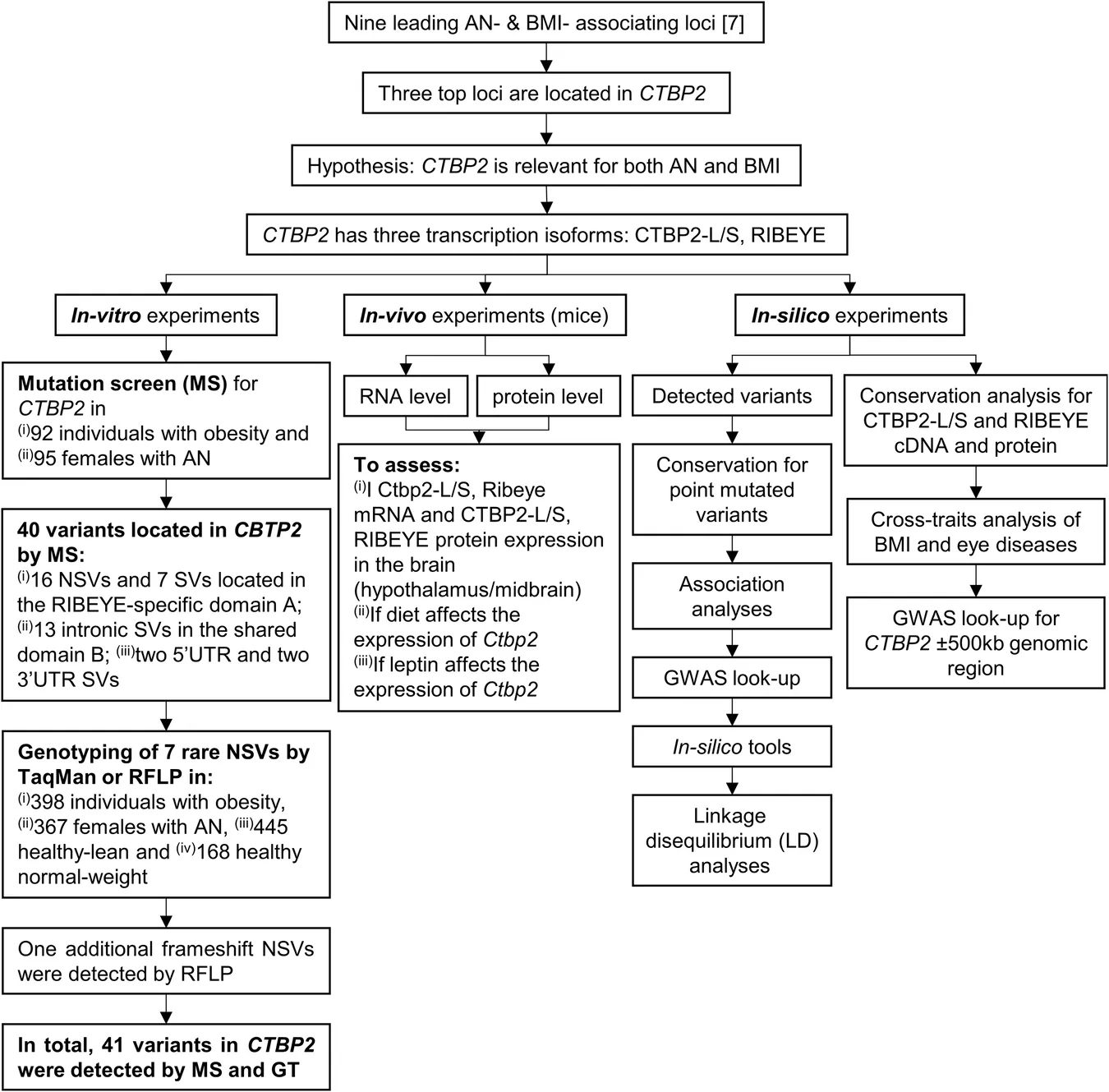
Workflow of the study. Based on previous analyses [ref for Hinney 2017, MolPsych], *Ctbp2* and its respective isoforms (CTBP2-L/S and RIBEYE) were analysed in in vitro, in vivo and in silico analyses. AN anorexia nervosa, BMI body mass index, Ctbp2 C-terminal binding protein 2, GT genotyping, MS mutation screen.

Hence, we performed genetic and functional studies to analyze *CTBP2* with all its isoforms (CTBP2-L/S and RIBEYE; Fig. 2). A mutation screen of *CTBP2’s* coding regions was conducted to detect variants that might confer a monogenic effect for the etiology of AN and/or body weight regulation. For the detected variants, in silico analyses ensued. The hypothalamus is the main center for energy homeostasis, whereas the ventral tegmental area (VTA) in the midbrain plays an essential role in the mesolimbic dopamine reward pathway, but is also required for feeding behavior [36, 37]. Experiments in mice were preformed to analyze the impact of fasting and re-feeding, a high-fat diet (HFD) and leptin on the expression of *Ribeye* in murine retina, hypothalamus, and midbrain (Fig. 2).

## Materials and methods

### In-depth analysis of the *CTBP2* gene and adjacent regions

To investigate whether the gene region of *CTBP2* and its ± 500 kb adjacent regions (chr10: 126,172,886–127,349,599; GRCh37) encompass variants with sexually dimorphic effects, the summary statistics of GWASs for AN [38] and BMI [39] were downloaded. For the BMI GWAS, datasets pertaining sex-combined (females + males) as well as sex-separated data were analyzed [39]. The GWAS data (genomic position vs. *p*-value) were plotted using GraphPad’s Prism (version 9.4.1). If there was a deviating number of significant SNPs (*p*-value < 5*10^−8^) between females and males, the Z-score was calculated (Supplementary Table 1) [40, 41]. A sexually dimorphic effect was present if |Z-score|> 2.

### Mutation screen and genotyping of rare non-synonymous variants (NSVs) in *CTBP2*

#### Study group

The mutation screen study group comprised 95 patients with AN (acute or recovered) [2] and 92 children and adolescents with severe obesity (86% with BMI percentile ≥ 97^th^). All participants were previously included in GWAS analyses with details pertaining the recruitment being described in the respective publications [9, 38, 42–44]. Certain rare NSVs were genotyped in larger independent study groups of 367 patients with AN (acute or recovered), 398 children and adolescents with severe obesity (95% with BMI-percentile ≥ 97^th^), 445 healthy-lean (BMI-percentile ≤ 15^th^) and 168 individuals with a normal body weight (40^th^ ≤ BMI-percentile ≤ 60^th^; Table 1).

**Table 1:** Phenotypic description of the analyzed study groups.

Study groups	Analyses	Study subgroups	*n*	Age (years)	Weight (kg)	Height (cm)	BMI (kg/m^2^)
				Mean ± SD	Mean ± SD	Mean ± SD	Mean ± SD
Patients with AN^a^	Mutation screen	All	95	23.63 ± 7.92	43.37 ± 7.68	166.33 ± 5.90	17.12 ± 2.58
		Acute	79	24.07 ± 7.95	45.21 ± 4.37	166.55 ± 5.78	16.29 ± 1.22
		Recovered	16	21.44 ± 7.35	58.06 ± 10.88	165.23 ± 6.34	21.22 ± 3.46
	Genotyping	All	367	20.12 ± 8.65	44.69 ± 9.03	165.20 ± 7.18	16.31 ± 2.80
		Acute	289	18.02 ± 7.10	41.44 ± 6.11	164.88 ± 7.29	15.20 ± 1.70
		Recovered	78	27.85 ± 9.43	56.73 ± 7.81	166.41 ± 6.63	20.44 ± 2.10
Children and adolescents with obesity	Mutation screen	All	92	13.91 ± 3.76	94.30 ± 33.38	163.34 ± 17.61	34.21 ± 8.66
		Female	48	14.13 ± 3.77	95.60 ± 34.89	160.65 ± 16.30	35.76 ± 9.84
		Male	44	13.66 ± 3.73	92.88 ± 31.60	166.28 ± 18.50	32.51 ± 6.76
	Genotyping	All	398	14.51 ± 3.73	89.80 ± 25.22	163.20 ± 12.68	33.19 ± 6.27
		Female	231	14.65 ± 3.67	87.05 ± 22.63	161.40 ± 11.44	32.97 ± 6.28
		Male	167	14.31 ± 3.79	93.64 ± 27.99	165.72 ± 13.83	33.51 ± 6.23
Healthy-lean controls	Genotyping	All	445	26.04 ± 5.77	57.11 ± 8.24	174.88 ± 9.6	18.08 ± 1.14
		Female	275	26.47 ± 6.38	52.1 ± 4.58	169.56 ± 6.56	17.58 ± 0.94
		Male	170	25.35 ± 4.56	65.21 ± 6.13	183.48 ± 7.2	18.89 ± 0.94
Normal weight controls	Genotyping	All	168	24.62 ± 2.49	67.01 ± 9.99	173.76 ± 9.4	21.55 ± 1.16
		Female	102	24.11 ± 2.48	60.05 ± 4.68	167.99 ± 5.99	20.71 ± 0.49
		Male	66	25.42 ± 2.3	77.54 ± 5.65	182.46 ± 6.41	22.81 ± 0.56

*SD* standard deviation.

^a^The included patients with AN were exclusively female.

Written informed consent was given by all participants and in case of minors by their parents. The study was approved by the Ethics committees of the Universities of Essen, Marburg, Aachen, Dresden, Frankfurt, Hannover, Heidelberg, Tübingen and Würzburg and was performed in accordance with the *Declaration of Helsinki* [45].

#### Mutation screen

To identify genomic variants, the coding regions of the *CTBP2* gene (ENSG00000175029, chr10: 124,984,317-125,161,170, GRCh38) were analyzed. The eight common exons for CTBP2 and RIBEYE as well as the additional exons for CTBP2-L (ENST00000337195.9) and RIBEYE’s A-domain (ENST00000309035.11; Fig. 1) were screened for variants in 95 female patients with AN and 92 children and adolescents with severe obesity (Table 1). Due to the location of the primer binding sites, flanking intronic regions were partially and, in some cases, even completely screened. All fragments were amplified in a polymerase chain reaction (PCR) using coding sequence spanning primers (designed with Primer3; Supplementary Table 2). Fragments were confirmed by a 2.5% agarose gel electrophoresis and subsequent unidirectional Sanger sequencing (LGC Genomics). All sequences were checked for variants by two experienced scientists using the SeqMan Pro software by DNAStar, Inc. (version: 10.1.0). In case of discrepancies or in presence of variants, samples were re-sequenced bi-directionally (MicroSynth Seqlab GmbH).

#### Genotyping

Rare NSVs exclusively detected in patients with AN (rs146900874 - p.Arg72Trp; rs375685611 - p.Val289Met and rs202010294 - p.Gly362Arg), or in children and adolescents with severe obesity (rs150867595 - p.Pro52Ser; rs141864737 - p.Gln175*fs*; rs769811964 - p.Leu310Val; rs76134089 - p.Pro397Ala and rs113477585 - p.Pro402Ser) were genotyped by either restriction fragment length polymorphism (RFLP) or TaqMan assays (Life Technologies). For RFLP, the restriction endonucleases *BanII* (rs769811964 - p.Leu310Val and rs202010294 - p.Gly362Arg), *HinfI* (rs150867595 - p.Pro52Ser) and *NlaIII* (rs375685611 - p.Val289Met) by New England Biolabs were used. TaqMan assays were employed for the following NSVs: rs146900874 (p.Arg72Trp; C_160707507_10), rs141864737 (p.Gln175*fs*; ANWCWZJ), rs76134089 (p.Pro397Ala; C_104707534_10) and rs113477585 (p.Pro402Ser; C_160138334_10). Both methods included a negative control (H_2_O) and at least one positive control from previously re-sequenced samples. Genotypes were independently rated by two experienced scientists. In case of discrepancies genotyping was repeated.

### In silico analyses for detected variants in *CTBP2*

#### Conservation analysis

Human CTBP2-L/S and RIBEYE complementary DNA (cDNA) and protein sequences were compared to 40 other species (ten primates, ten rodents and related species, ten laurasiatherians, eight fishes, two sauropsidas; Supplementary Table 12). For all analyzed species, both transcripts (CTBP2-L/S and RIBEYE) were available. The sequences of cDNAs and proteins were extracted from Ensembl [46] and were aligned in MegAlign (DNASTAR Lasergene 11, version 11.0.0) using the ClustalW method. The conservation percentiles were calculated for all detected coding region SNPs.

#### GWAS look-up for *CTBP2* and detected variants

The detected variants were looked-up for the *p*-values and effect sizes (*β*) in GWAS datasets for AN and BMI (datasets pertaining combined sexes as well as separate datasets for females and males) [38, 39].

#### Pathogenicity prediction of detected SNPs

Variants detected in the coding region of *CTBP2/RIBEYE* were assessed pertaining their putative pathogenicity by applying multiple in silico tools, such as MutationTaster2 [47], Combined Annotation Dependent Depletion (CADD) [48] and PredictSNP2 [49]. A series of in silico analyses on NSVs to explore their impact on protein stability and function ensued. The pathogenicity of amino acid substitutions were evaluated with Polymorphism Phenotyping v2 (Polyphen2.0) [50], Protein Variation Effect Analyzer v1.1 (PROVEAN) [51], Sorting Intolerant from Tolerant (SIFT) [52] and HOPE [53]. Three programs, MUPro [54], I-Mutant 2.0 [55] and iStable 2.0 [56], were applied to predict effects on protein stability. For synonymous variants (SVs), one tool relied on nucleotide alterations (Transcript-inferred Pathogenicity, TraP) [57] and three tools analyzed splice site alterations (ESEfinder 3.0, Spliceman, SpliceAI) [58–60].

#### Analysis of the linkage disequilibrium (LD) of detected variants

To investigate the LD structures of the detected variants, LDmatrix was utilized. Thus, all detected variants, our previously identified SNPs associated with increased AN-risk and decreased BMI (rs11245456, rs12771627, rs1561589) [9], as well as genome-wide significant SNPs (*p*-value < 5*10^−8^) located either in the *CTBP2* gene region or its ± 500 kb adjacent region [39], were used to construct the linkage map in LDmatrix. Variants in strong LD (D’ > 0.8, R^2^ > 0.3) with SNPs associated with AN were further analyzed regarding their haplotypes using LDpair. Both LDmatrix and LDpair are accessible via LDlink and genotype data of 99 CEU (Utah residents with Northern and Western European ancestry) individuals from the 1000 Genomes Project Phase 3 available at ENSEMBL was used.

#### Animals

For fasting and re-feeding experiments, male C57BL/6J mice and leptin deficient (*Lep*^*ob/ob*^) mice were obtained from the Jackson Laboratory. The male C57BL/6J mice were 27/28 weeks old. They were fed *ad libitum* with a standard chow diet, while some animals were fasted for 12 h, 24 h or 36 h. Another subgroup of animals was fasted for 36 h and then re-fed for 6 h with either a fat-free diet (FFD) or high-fat diet (HFD). Each group comprised 6–8 animals. For RNA analyses, the hypothalamus of these animals was extracted. These experiments were performed in Cincinnati, OH, USA with the approval of the Animal Ethics Committee of Cincinnati, OH, USA. The experimental design was based on previously performed animal models [61].

The hypothalamus and midbrain of *Lep*^*ob/ob*^ mice (*n* = 18) were extracted. These adult mice were chow fed. Further, a DIO mouse model was generated by exposing young C57BL/6J mice to a HFD over six months. Mice were euthanized in CO_2_. Perfusion was performed with saline, followed by a 4 °C cooled 4% paraformaldehyde in 0.1 M PBS (pH 7.4).

For expression analyses on mRNA and protein levels, hypothalamic and retina of female wildtype C57BL/6J mice were obtained from the Institute of Neuropathology of the University Hospital Essen (Keyvani’s lab). Permission for mice breeding and decapitation was granted by the local committee LANUV NRW, Germany (AZ 84-02.04.2014.A488).

All animal experiments were carried out in accordance with the EU Directive 2010/63/EU and complied with the ARRIVE guidelines. No blinding of the investigators was performed. Animals were assigned to the different study groups without a specific randomization.

All mice were maintained at a constant temperature (22 ± 1 °C), relative humidity and a 12-h light/dark cycle. All animals had free access to water and were fed either a HFD consisting of 58% kcal fat (D12331; Research Diets) or a normal chow diet consisting of 5.6% kcal fat (Harlan Teklad LM-485).

#### Quantification of *Ribeye* gene expression

To investigate effects of fasting and re-feeding on *Ribeye* gene expression, male C57BL/6J mice were fed *ad libitum* with a standard chow diet. Some animals were fasted for either 12 h, 24 h or 36 h, or were fasted for 36 h and then re-fed with a FFD or a HFD for six hours (*N* = 6–8 mice per group). Total RNA was extracted from murine hypothalamus using TRIzol (Invitrogen Life Technologies) and treated with DNAse I (New England BioLabs). RNA was subsequently reversely transcribed into cDNA which was used as a template for a two-step quantitative real-time PCR (qRT-PCR). For each reaction, 2 ng/µl cDNA, 1 µM specific forward primers, 1 µM specific reverse primers, 1 U SYBR Green master mix (Life Technologies) was used. The used primers to amplify *Ribeye*’s A-domain were as follows: forward 5’-GCAAGAGGACCATGTACCCT-3’ and reverse 5’-TCCTGTCTCCGAAACTGCAT-3’. The specific primers for murine *Ctbp2* shared B-domain were described in our previous study [9]. Amplification of *Ribeye* and *Ctbp2* was conducted on the ViiA 7 realtime PCR system (Life Technologies). Results were normalized to the housekeeping gene hypoxanthine guanine phosphoribosyltransferase 1 (*Hprt*).

To assess the effects of HFD on *Ribeye* mRNA expression in the hypothalamus, DIO mice (*N* = 8, weight: 54.72 ± 1.25 g) and age-matched C57BL/6J fed a regular chow diet (*N* = 7, weight: 32.69 ± 0.45 g) were analyzed. Subsequently, the expression of *Ctbp2* and *Ribeye* in the hypothalamus and midbrain of leptin-deficient mice was investigated with adult chow-fed *Lep*^*ob/ob*^ mice receiving subcutaneous daily injections for six days of either human recombinant leptin (1 mg/kg, R&D Systems, *N* = 6) or a vehicle containing PBS (*N* = 6). An additional pair-fed group of vehicle-treated *Lep*^*ob/ob*^ mice (*N* = 6) had access to a restricted amount of food to match the leptin-treated mice’s food intake. For details on the pair-fed experiment please refer to a previous study by Kabra et al. [62].

### Verification of *Ribeye* in the hypothalamus

#### Qualitative study on hypothalamic *Ribeye* mRNA expression

Total RNA from hypothalamus and retina of female C57BL/6J mice was extracted. Again, a DNase I treatment and a RT-PCR using 500 ng to 1 µg RNA and a commercial RT-Mix (Quanta Bio) was performed. Two-step RT-PCR was then performed with the same amounts of cDNA as before and specific primers for murine *Ribeye* cDNA. In addition, a nested PCR was performed using three primers (one common forward and two reverse), which were positioned to span the intron to exclude amplification of genomic DNA (Supplementary Fig. 1). The used primer sequences were *Ribeye*-F: 5’-ACTGCTTAAGAGGGAACGCA-3’; *Ribeye*-R: 5’-CATCACAGAAGGCCACAGTG-3’; *Ribeye*-R1: 5’-GCATCTCCACAGTGCAGTCTC-3’. The two reverse primers served to perform the nested PCR (Supplementary Fig. 1) to increase product specificity and sensitivity. Murine retina samples known to express *Ribeye* [18] acted as positive controls. PCR products were purified using the QIAquick PCR Purification Kit (Qiagen GmbH) and commercially sequenced in both directions by MicroSynth SeqLab GmbH. Sequences were evaluated in house (DNAStar Version 10.1.0, Lasergene).

#### Protein isolation

Isolated frozen murine hypothalami and retina from female C57BL/6J mice were homogenized in RIPA (radio immune precipitation assay; Sigma Aldrich) containing a protease inhibitor (cOmplete, EDTA-free Protease Inhibitor Cocktail, Roche) and phosphatase inhibitor (Sigma Aldrich) with the FastPrep-24 5 G System (MP, Biomedicals) for 30 s at 6.0 m/s. To remove cell debris, samples were centrifuged twice for 5 min and once for 10 min at 10,000 × *g* and 4 °C. The supernatant was collected, and protein concentration was determined using the BCA protein kit (Thermo Scientific).

#### Immunoblotting

All immunoblot experiments were performed at least in triplicate at the Lead Discovery Center (LDC) in Dortmund. Lysates were diluted 2:1 in 3x SDS Laemmli Buffer and boiled at 96 °C for 5 min. Equal amounts of protein were separated by SDS-PAGE on 4–20% Mini-PROTEAN TGX Precast Gels (Bio-Rad) and transferred onto Immobilon-FL PVDF membranes (Merck). The membranes were blocked with Odyssey blocking buffer (LI-COR Biosciences) for 1 h and washed thrice with 1x Phosphate Buffered Saline + Tween 20 (PBST). Afterwards, the membranes were incubated with the primary mouse anti-RIBEYE(B)-domain/CTBP2 (1:200 dilution; C-terminus, bind to aa 977 to 988, clone 2D9, molecular weight of protein: 50 kDa) [63] and mouse anti-RIBEYE(A)-domain antibodies (1:500 dilution; N-terminus, bind to aa 95 to 207, clone 12A10; molecular weight of protein: 110 or 120 kDa), respectively, in Odyssey blocking buffer at 4 °C over the weekend under gentle agitation. Both primary antibodies were kindly provided by Prof. Dr. F. Schmitz, University of Saarland, Institute of Anatomy and Cell Biology, Germany. Next day, each membrane was incubated with one fluorescence-conjugated donkey anti-mouse secondary antibody (1:10.000 dilution; LI-COR Biosciences, #926-32212) for 1 h at room temperature (RT) and protected from light. After that, the membranes were washed with 1x PBST three times, then scanned using the Odyssey Infrared Imager (LI-COR Biosciences) and visualized with the Image Studio 5.x for Odyssey CLx Image Acquisition Program. Three independent experiments were performed, and a positive control was derived from murine retina samples [18]. For protein loading control, each membrane was additionally incubated with the primary rabbit anti-Alpha-Tubulin antibody (1:5.000 dilution; Sigma Aldrich; molecular weight of protein: 55 kDa) for 1 h at RT and a fluorescence-conjugated donkey anti-rabbit secondary antibody (1:10.000; LI_COR Biosciences #926-68023) for 1 h at RT and protected from light. As above, after each incubation step, membranes were washed three times with 1x PBST.

#### Statistics

The Hardy–Weinberg equilibrium, and either the Chi-square (*χ*^2^) or the Fisher’s exact test (alternative allele count less than five) as well as the odds ratio were calculated for variants detected more than once. *p*-values were Bonferroni corrected. Healthy-lean and normal weight individuals were recruited as controls for rare NSVs detected in the genotyping, whereas the gnomAD v3.1.2. European, non-Finnish population served as control groups to analyze associations between traits and variants detected by Sanger sequencing (Supplementary Tables 7–9 and 11).

For all animal experiments, statistical analyses were performed using GraphPad’s Prism or SPSS. One-way ANOVA with Tukey’s multiple comparison *post hoc* test was applied to examine the differences between groups. No differences of variance between the groups were observed with the Bartlett’s test for equal variance which was performed prior to the ANOVA. Data is represented as mean ± SEM (standard error of mean) and *p*-values ≤0.05 were considered significant.

## Results

### In-depth analyses of *CTBP2* ± 500 kb adjacent regions

Initially, we checked whether the gene region of *CTBP2* ± 500 kb adjacent regions (GRCH37; chr10: 126,172,886~127,349,599) comprise genome-wide significant variants (*p* < 5*10^−8^) for AN and BMI (datasets for male and female combined and separate analyses) based on the latest GWAS datasets [38, 39]. In all analyzed regions, no variant reached genome-wide significance for AN (Supplementary Fig. 2A). Yet, a large number of genome-wide significant SNPs for BMI in females was found (~83% significant SNPs were located in *CTBP2*, Supplementary Fig. 2C), whereas no variant exceeded the significance threshold in males (Supplementary Fig. 2D). For the female-associated SNPs, Z-scores were calculated to identify putative sexually dimorphic SNPs. One SNP (rs12220302) was determined to be sexually dimorphic exhibiting stronger BMI-altering effects in females than males (Supplementary Table 1).

### Mutation screen reveals 23 variants in *RIBEYE*’s A-domain

To identify genetic variants that might be implicated in AN and/or weight regulation, we performed a mutation screen of *CTBP2* in 95 female patients with AN and 92 children and adolescents with severe obesity (including 48 females). Our screen covered the eight common exons of CTBP2 and RIBEYE (B-domain) as well as the 1A exon of CTBP2-L and exons of the A-domain of RIBEYE (Fig. 1). A total of 23 variants within the coding region were detected (Supplementary Table 3). All of these were found in the A-domain specific for RIBEYE. Sixteen were NSVs, including one frameshift (rs1411864737 - p.Gln175*fs*) and one in-frame variant (rs372118432 - p.Pro391_Leu392*Ins*). Three NSVs (rs146900874 - p.Arg72Trp; rs375685611 - p.Val289Met and rs202010294 - p.Gly362Arg) were rare with frequencies below 1% and were identified exclusively in patients with AN. Four rare NSVs (rs150867595 - p.Pro53Ser; rs769811964 - p.Leu310Val; rs76134089 - p.Pro397Ala and rs113477585 - p.Pro402Ser) and one frameshift variant (rs141864737 - p.Gln175*fs*) were observed only in children and adolescents with severe obesity (Supplementary Table 3). One rare NSV (rs535621897 - p.Glu377Asp) was detected in both study groups (patients with AN and obesity) in similar frequencies (MAF: 0.54%; Supplementary Table 3). All detected variants were in Hardy–Weinberg Equilibrium.

None of the detected variants was associated with AN or obesity (Supplementary Tables 10 and 11).

As flanking intronic sequences were screened due to the localization of the primer binding sites, 13 intronic and four untranslated region (UTR) variants were identified (Supplementary Table 4). These were not considered for any follow-up analyses.

### Genotyping for rare NSVs in independent study groups

Subsequently, we genotyped rare variants exclusively found either in patients with AN or in children and adolescents with severe obesity in larger independent study groups of 367 females with AN, 398 children and adolescents with severe obesity (including 231 females), 445 healthy-lean individuals (including 275 females) and 168 participants with normal weight (including 102 females). Three NSVs (rs375685611 - p.Val289Met; rs769811964 - p.Leu310Val and rs113477585 - p.Pro402Ser) identified in our preceding mutation screen (Supplementary Table 3) were not detected in additional individuals (Supplementary Tables 5 and 6). During the genotyping process of the rare NSV rs150867595 (p.Pro53Ser), a heterozygous frameshift variant p.Val132Ala*fs*Ter35 (rs1379972000) which was not detected in our mutation screen was identified in one female patient with obesity (Supplementary Tables 3, 5 and 6).

rs202010294 (p.Gly362Arg) was detected in ten independent females in all study groups. No male carriers were identified (Supplementary Tables 5 and 6). The variant rs146900874 (p.Arg72Trp) detected in one female patient with AN in our mutation screen, was identified in one additional healthy-lean female (Supplementary Tables 3, 5 and 6).

Again, no associations of the SNPs and traits (AN and obesity) were determined when analyzing the larger study groups and various control groups (healthy-lean and normal-weight controls and gnomAD’s European, non-Finnish population; Supplementary Tables 7–11).

### In silico analyses

#### Conservation analysis for CTBP2-L/S and RIBEYE

To assess sequence distances, human RIBEYE and CTBP2-L/S cDNA and protein sequences were compared to 40 species from 5 superorders (Supplementary Table 12). In all ten primates, CTBP2-L/S and RIBEYE are highly conserved on both cDNA and protein level. Four NSVs (rs3781409 - p.Val234Met; rs3781411 - p.Arg298Gln; rs76134089 - p.Pro397Ala and rs2946994 - p.Gln539Glu) exhibited a high conservation percentile of > 90% (Table 2, Supplementary Table 13 and Supplementary Figs 3 and 4).

**Table 2:** In silico analyzes for detected variants applying multiple tools.

SNP ID	AA alteration	Cper. (%)^a^		Pathogenicity		RNA splicing^d^	Protein stability^e^	
		RIBEYE		NT^b^	AA^c^			
		Protein	cDNA	i/n^f^	j/m^g^	k/h^h^	Decreased	Increased
rs150867595	p.Pro53Ser	78.05	73.17	3/3	3/3	NA.	6	0
rs146900874	p.Arg72Trp	95.12	51.22	3/3	1/2	NA.	6	0
rs3781407	p.Asp195=	56.10	NA.	0/3	NA.	0/2	NA.	NA.
rs116403181	p.Ser212=	75.61	NA.	0/3	NA.	0/2	NA.	NA.
rs3781408	p.Asp213Asn	82.93	82.93	0/3	0/3	NA.	6	0
rs3781409	p.Val234Met	**92.68**	**90.24**	**3/3**	**1/2**	**NA**.	**6**	**0**
rs45440394	p.Arg287=	87.80	NA.	1/3	NA.	0/2	NA.	NA.
rs375685611	p.Val289Met	80.49	68.29	0/3	1/3	NA.	6	0
rs3781411	p.Arg298Gln	**90.24**	**90.24**	**3/3**	**2/2**	**NA**.	**6**	**0**
rs769811964	p.Leu310Val	87.80	92.86	1/3	2/3	NA.	5	1
rs142101185	p.Ser331=	60.98	NA.	1/3	NA.	0/2	NA.	NA.
rs202010294	p.Gly362Arg	68.29	65.85	0/3	2/3	NA.	6	0
rs45535234	p.Ser376=	51.22	NA.	1/3	NA.	1/2	NA.	NA.
rs535621897	p,Glu377Asp	34.15	78.05	0/3	0/2	NA.	4	2
rs3781412	p.Leu392Pro	92.68	75.61	2/2	1/2	NA.	5	1
rs76134089	p.Pro397Ala	92.68	92.68	2/2	3/3	NA.	4	2
rs113477585	p.Pro402Ser	70.73	39.02	0/2	2/2	NA.	4	2
rs3781413	p.Ala418=	31.71	NA.	0/3	NA.	2/2	NA.	NA.
rs3012075	p.Tyr455His	92.68	85.37	0/3	1/2	NA.	6	0
rs894087529	p.Pro466=	46.34	NA.	1/3	NA.	1/2	NA.	NA.
rs2946994	p.Gln539Glu	90.24	90.24	2/3	1/2	NA.	3	3

Variants shown in bold are non-synonymous variants with high conservation percentage that may cause decreased protein stability and function alteration.

*AA* amino acid, *NA* not applicable in our study.

^a^Cper. (%): conservation percentage in percentile.

^b^NT: deleteriousness analysis for nucleotide exchange (both SVs and NSVs).

^c^AA: deleteriousness analysis for amino acid exchange (NSVs).

^d^RNA splicing: the effect of synonymous variants (located in coding and non-coding region) on RNA splicing.

^e^Protein stability: the effect of non-synonymous coding variant on protein structure.

^f^i/n: the count of in silico tools which predicted pathogenic (i) in (n) accessible software for NT deleteriousness analyses.

^g^j/m: the count of in silico tools which predicted pathogenic (j) in (m) accessible software for AA deleteriousness analyses.

^h^k/h: the count of in silico tools which predicted as the variant may affect RNA splicing pattern (k) in (h) accessible software.

#### GWAS look up for all detected variants

All coding region located variants detected either in our mutation screen or genotyping approach were looked up in GWAS for BMI and AN [38, 39] (Supplementary Tables 14 and 15). Three NSVs (rs3781409 - p.Val234Met; rs3012075 - p.Tyr455His and rs2946994 - p.Gln539Glu) are associated with BMI in both sexes combined and in females. No variant was relevant for BMI in males or for AN (Supplementary Tables 14 and 15).

#### Pathogenicity of detected variants analyzed in multiple in silico tools

To assess the functional relevance of the variants located in the coding region, several in silico tools were applied (Supplementary Tables 16–20). The reported frameshift variants (rs1379972000 - p.Val132*fs* and rs141864737 - p.Gln175*fs*) change the protein structure and function due to alterations of amino acid sequences and its length. Four NSVs and two SVs were predicted to be pathogenic in all estimated scales (overall pathogenicity of nucleotide and amino acid exchange, protein stability and RNA splicing patterns; Table 2 and Supplementary Tables 16–20).

#### Linkage disequilibrium analyses

LD analyses were conducted with LDmatrix and LDpair. Initially, we assessed whether the identified variants located in the coding regions of *CTBP2* are in LD with the SNPs previously reported by us to be associated with increased AN risk and decreased BMI [9]. Four NSVs (rs3781409 - p.Val234Met; rs3781412 - p. Leu392Pro; rs3012075 - p.Tyr455His and rs2946994 - p.Gln539Glu) were found to be in LD (R^2^ > 0.3 & D’ > 0.8) with two of the AN and BMI overlapping SNPs (rs12771627 and rs1561589; Supplementary Table 21) [9]. None of the identified variants was in LD with rs11245456 (Supplementary Table 21) [9].

Subsequently, we investigated the LD structures of the detected variants and genome-wide significant SNPs for BMI which were either located within *CTBP2* or adjacent regions (±500 kb) [39]. Again, the four variants (rs3781409 - p.Val234Met; rs3781412 - p. Leu392Pro; rs3012075 - p.Tyr455His and rs2946994 - p.Gln539Glu) which are in strong LD with the AN and BMI overlapping SNPs [9] were in LD with multiple GWAS hits [39] (Supplementary Tables 22 and 23). A perfect LD (R^2^ = 1; D’ = 1) was found for rs3781409 (p.Val234Met) and 12 BMI-associated variants (Supplementary Tables 22 and 23). These twelve variants are located within ~18,000 bp of rs3781409. Further, 14 additional BMI-associated variants were found to be in high LD (R^2^ > 0.9, D’ > 0.8) with rs3781409 (Supplementary Tables 22 and 23). Additionally, rs3012075 (p.Tyr455His) was in perfect LD (R^2^ = 1; D’ = 1) with the BMI-associated SNP rs2363893 [39].

#### Presence of *Ribeye* mRNA and RIBEYE protein expression in murine hypothalamus

A previous study demonstrated that CTBP2-L/S protein is expressed in all analyzed rat tissues, while RIBEYE protein was exclusively detected in the retina [18]. Here, we aimed to evaluate RIBEYE’s impact on food intake which is primarily controlled in the hypothalamus [37, 64] and thus examined whether *Ribeye* and its respective protein are present in the brain. We extracted RNA from murine hypothalamus and retina of female wildtype C57BL/6J mice and performed a nested RT-PCR with subsequent Sanger-sequencing. For both hypothalamus and retina, mRNA of *Ribeye* was verified (Supplementary Fig. 5).

To assess RIBEYE protein expression, 30 µg of total protein from hypothalamus and retina of female wildtype C57BL/6J mice were employed to immunoblotting using two specific antibodies: one against the A-domain of RIBEYE and another recognizing the shared B-domain of RIBEYE and CTBP2. In retina controls, two RIBEYE-specific protein bands were detected as previously described [65–68]. In contrast, RIBEYE-specific protein bands were absent in samples of murine hypothalami (Supplementary Fig. 6).

#### Quantification of *Ribeye* expression in response to fasting and re-feeding

**Fig. 3:**
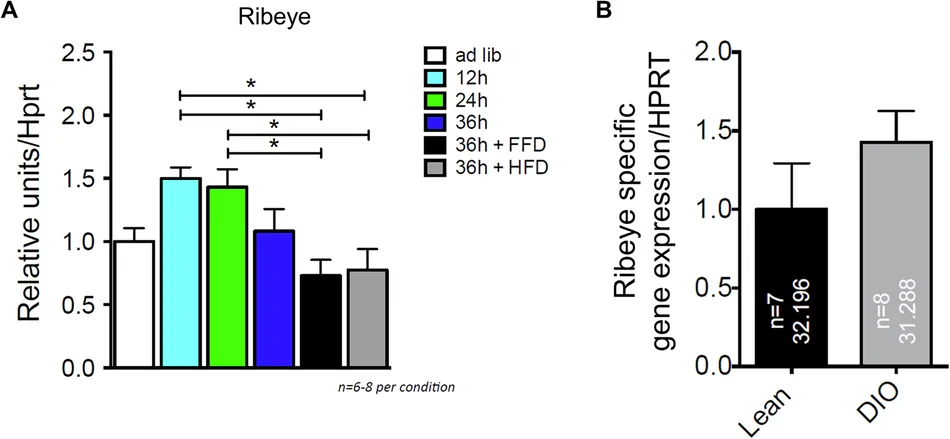
Gene expression profiling of *Ribeye* mRNA in murine hypothalamus. **A** Hypothalamic expression in mice fed *ad libitum* (ad lib), in mice fasted for 12 h, 24 h or 36 h or in mice fasted for 36 h with subsequent re-feeding for 6 h with either a fat-free diet (FFD) or a high fat diet (HFD). **B** Hypothalamic expression profiles for diet-induced obesity (DIO) and age-matched lean control mice. All results were normalized to the housekeeping gene *HPRT*. Average of Ct values are displayed in the respective bar graphs. One-way analysis of variance (ANOVA) with Tukey’s multiple post-hoc comparison test. HPRT hypoxanthine guanine phosphoribosyltransferase 1. *n* number of samples.

In our previous study, we reported that expression levels of *Ctbp2* (combined effect of both transcriptional isoforms) decrease after fasting and remained reduced after re-feeding in male C57BL/6J mice, regardless of the diet given after fasting [9]. Here, we examined the impact of fasting and re-feeding on *Ribeye* expression in the hypothalamus of male C57BL/6 J mice. RNA from murine hypothalamus that were either fed *ad libitum* with a regular chow diet, were fasted for 12 h, 24 h or 36 h or were fasted for 36 h and then re-fed with either a FFD or HFD were applied to qRT-PCR. Comparison between mice fed a chow diet (*ad libitum*) and mice fasted for different durations (12 h, 24 h or 36 h) revealed no alteration in hypothalamic expression of *Ribeye* mRNA (Fig. 3A). Yet, the hypothalamic *Ribeye* expression is downregulated after refeeding with FFD and HFD compared to fasted conditions without refeeding (Fig. 3A).

Subsequently, the *Ribeye* mRNA expression was analyzed in a DIO mouse model. As seen in the fasting and re-feeding experiments, no difference in mRNA expression was found between DIO and lean mice (Fig. 3B).

**Fig. 4:**
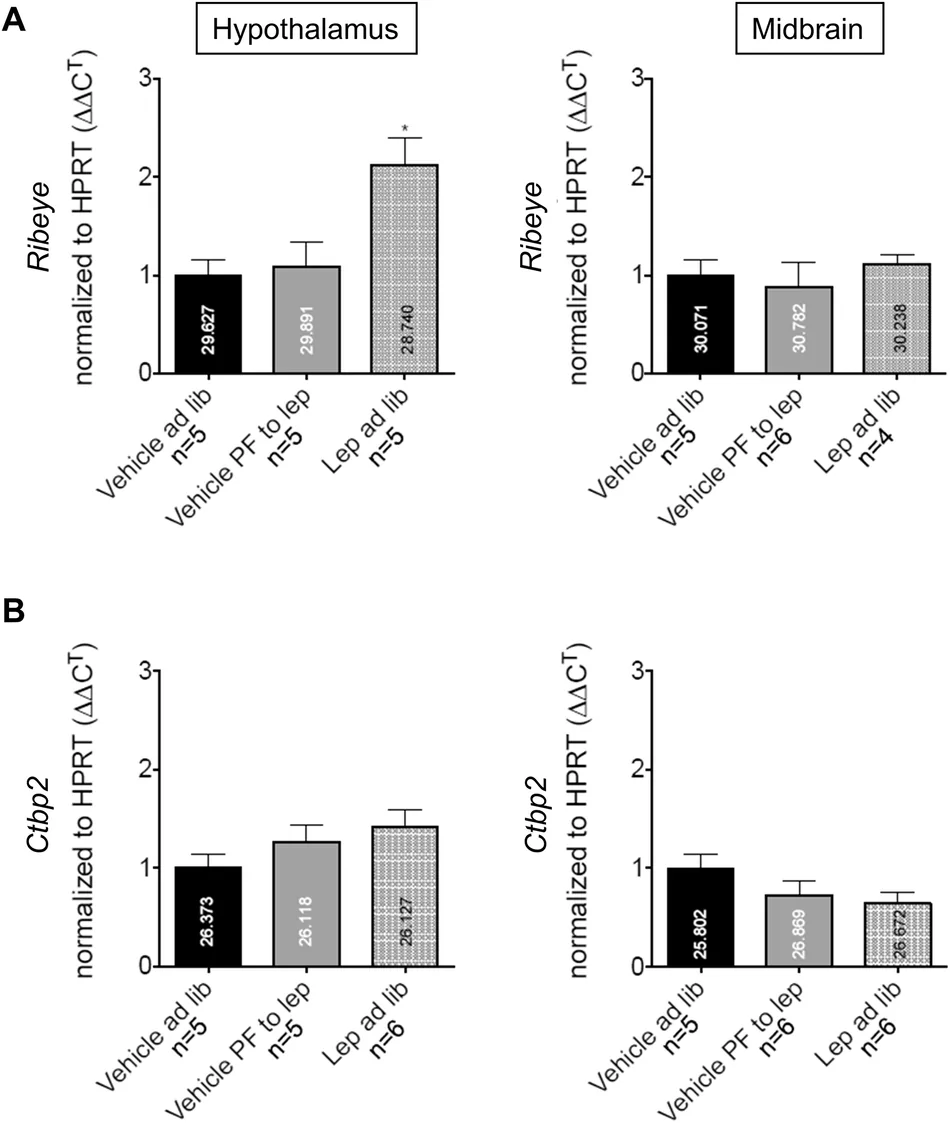
Effect of leptin administration and pair feeding of *Lep*^*ob/ob*^ mice on hypothalamic and midbrain Ribeye and Ctbp2 mRNA expression. *Ribeye* (**A**) and *Ctbp2* (**B**; all isoforms) expression analyses were performed in vehicle-treated *Lep*^*ob/ob*^ mice fed *ad libitum* (ad lib), in vehicle-treated pair-fed (PF) *Lep*^*ob/ob*^ mice and in leptin-treated *Lep*^*ob/ob*^ mice. *p < 0.05, based on a one-way ANOVA with Tukey’s multiple post-hoc comparison test. Average of Ct values are displayed in the respective bar graphs, HPRT hypoxanthine guanine phosphoribosyltransferase. *n* number of mice used.

Afterwards, we investigated the effects of leptin on *Ctbp2* (both isoforms) and *Ribeye* mRNA expression in hypothalamus and midbrain of leptin-deficient *Lep*^*ob/ob*^ mice as these brain regions are major appetite and energy homeostasis regulators [25, 36, 37]. Chow fed *Lep*^*ob/ob*^ mice were treated subcutaneously either with recombinant leptin (daily injections: 1 mg/kg) or PBS. Pair-fed *Lep*^*ob/ob*^ mice were used to analyze if putative expression differences are due to variations in food intake [62]. We observed an increased expression of *Ribeye* mRNA in the hypothalamus of leptin-treated *Lep*^*ob/ob*^ mice compared to the vehicle-treated control mice (Fig. 4A). *Ribeye* expression in the midbrain did not change (Fig. 4A). The observed increase in hypothalamic expression is not due to a reduced food intake, as *Ribeye’s* expression does not differ between vehicle-treated mice fed with an *ad libitum* diet and the pair-fed vehicle-treated mice (Fig. 4A) and was not caused by a leptin-induced weight loss, since pair-fed and leptin-treated *Lep*^*ob/ob*^ mice showed a similar weight loss (not shown).

In contrast to *Ribeye*, *Ctbp2* mRNA levels did not differ in the hypothalamus of *Lep*^*ob/ob*^ mice after leptin administration compared to PBS-treated pair-fed control mice (Fig. 4B).

## Discussion

Previously, we have identified a locus at *CTBP2* containing three SNPs (rs1561589, rs12771627 and rs11245456) associated with increased AN-risk and a decreased BMI [9]. *Ctbp2*’s expression was decreased in fasted mice, while DIO mice showed an increased expression compared to age-matched controls [9]. Here, we initially screened the coding regions of *CTBP2* in patients with AN and severe obesity reporting 24 variants located in the RIBEYE specific exon (A-domain). This localization was unexpected as to date no study had linked RIBEYE to body weight-related traits, though the role of *CTBP2* in obesity is well established [16, 26–28, 69].

For the first time, we demonstrate evidence that *Ribeye* mRNA is present at a very low level in the murine hypothalamus suggesting that the expression either occurs only in few specific hypothalamic cell populations, or that *Ribeye* might be transcribed as an illegitimate transcript [70]. Illegitimate transcription is known as very low transcription of all genes in virtually all cell types [70]. Such transcripts are usually detected infrequently in e.g. fibroblasts and lymphoblasts of patients with Duchenne dystrophy [71]. To validate if *Ribeye* mRNA is expressed in an illegitimate manner, we analyzed the protein expression via western blot. As none of the antibodies detected RIBEYE protein in the murine hypothalamus, we can assume that the initial finding of *Ribeye* mRNA expression is indeed due to an illegitimate transcript. Of note, some reports conclude that there is often no correlation between mRNA and protein quantity [72–74]. Yet, the lack of RIBEYE protein detection may be due to the total amount being too low to be detected by western blotting, e.g., if it is only expressed in few sub-nuclei of the hypothalamus.

Determining protein expression levels requires consideration of multiple processes apart from transcript concentration, like translation rates, modulation, time of protein synthesis and protein transport [74]. Further, it is plausible that microRNAs (miRNAs) have degraded or at least inhibited the translation of the *Ribeye* mRNA in the hypothalamus. This is particularly intriguing as it is known that the highest density of miRNAs is found on the X chromosome [75]. Here, we have detected this mRNA-protein imbalance in hypothalami of female mice. Additionally, our sex-specific analyses revealed one SNP (rs12220302) in *CTBP2* with a stronger effect on the BMI in females than in males. As the present study is also based on findings that the strongest BMI signals regarding *CTBP2* were derived exclusively from females (*P*_overall_: 2.47*10^−6^, *P*_male_: 0.0043 and *P*_female_: 3.45*10^−7^) [61] and AN is predominantly diagnosed in young female adolescents [4], putative sex-specific effects due to the sex chromosomes or hormones are plausible.

RIBEYE, a major component of synaptic ribbons, is expressed in tissues, where these band-like structures are found: photoreceptor and bipolar cells of the retina, hair cells of the inner ear and pinealocytes of the pineal gland [18, 24]. Interestingly, there is an intricate connection between the pineal organ and the hypothalamus in humans. For the production of melatonin, the regulator of the circadian rhythm, signals concerning the light status are sent from the retina via the suprachiasmatic nucleus (SCN) in the hypothalamus into the pineal organ. Hence, we can assume that RIBEYE may be expressed in the SCN. A differential regulation of RIBEYE in the hypothalamus could be due the presence of the SCN in the dissected murine hypothalami. Furthermore, the SCN is located next to the arcuate nucleus (ARC), known to be involved in food intake regulation [76]. Recently, an essential interaction between these two nuclei was shown [77]. This is necessary for an appropriate response to catabolic and metabolic conditions, as well as for a proper function of the circadian system. Herrera-Moro et al. demonstrated a time-oriented neuronal activity of the ARC in response to hypoglycemia in rats, suggesting an SCN involvement [77]. The SCN appeared to act in an inhibitory manner on the ARC, since unilateral SCN lesion activated ARC neurons in the same side of lesion [77]. Another study reported a time-depend influence of the SCN on the ARC, since light given during the dark phase inhibited ARC alpha-melanocyte-stimulating hormone (α-MSH) neurons in male rats. This α-MSH cell activation at the end of the dark phase persisted in *ad libitum* and fasted rats, indicating that food intake not only triggers α-MSH neuronal activity [78].

Leptin also targets the ARC. Leptin suppresses the activation of the neuropeptide Y and agouti-related protein neurons resulting in decreased appetite [79]. Here, we showed the impact of leptin on the expression of *Ribeye* in *Lep*^*ob/ob*^ mice. Leptin-stimulated *Ribeye* expression was not related to reduced food intake and leptin-induced weight loss. In addition, the increased expression of *Ribeye* could only be observed in the hypothalamus and not in midbrain, confirming the expression of *Ribeye* in the hypothalamus. It is already known that leptin may act as modulator in different hypothalamic functions like the circadian rhythm, as receptors for leptin (LepRb) were detected in the SCN [80] and in neurons of the lateral hypothalamic area (LHA; [64, 81]) that are involved in sleep/wake cycles [82]. LHA LepRb neurons mediate the inhibition of orexin neurons by leptin causing hyperpolarization and decrease orexin expression in the murine hypothalamus [83]. Plasma leptin levels in mice, rats and humans are increased during the night [84–86], whereas circulating leptin in hamsters showed a diurnal peak [87]. Thus, leptin may affect the SCN either directly by binding to SCN neurons or via its impact on the ARC [88]. At treatment initiation, serum leptin levels in patients with AN are lower than those of BMI-matched controls due to reduced fat mass and energy supply [89–91]. With weight gain during treatment, patients with AN develop leptin levels that are well above the reference levels of the control group, resulting in loss of appetite and subsequently in food denial [92]. As a consequence, the weight of the patients stagnates or even decreases. Thus, this abnormally high leptin level increase could be the reason for the difficulty for patients with AN to regain normal weight [92]. However, the contribution of these leptin fluctuations to the maintenance of the disease is still unclear. This warrants further analysis of the interaction between leptin and RIBEYE in AN. Cells overexpressing RIBEYE might be treated with different concentrations of leptin which would allow an evaluation whether RIBEYE protein and mRNA expression is up- or down-regulated in response to leptin. A similar experimental approach can be used to analyze the here detected SNPs' effect on the body weight, by analyzing human cells where the respective SNP was introduced (e.g. by CRISPR-Cas9) in response to varying leptin concentrations [93].

*RIBEYE* mutations may impact weight regulation by projections from intrinsically photosensitive retinal ganglion cells (ipRGCs), a third type of photoreceptors expressing the photopigment melanopsin [94]. It had recently been shown that light via ipRGCs projections to the thalamic perihabenular nucleus (PHb) affects mood, and to the SCN regulates cognition such as learning without disarranging the circadian clock [95]. Next steps would be to determine the exact nuclei of the hypothalamus in which RIBEYE is expressed and to analyze its function, which could be directly through its hypothalamic expression or indirectly through light-induced projections. Additionally, RIBEYE is a major component of synaptic ribbons [19] found in melatonin-secreting neurons [17, 21]. Evidence emerged that (exogenous) melatonin might affect food intake and body weight regulation (e.g. refs. [96–98],). Patients with AN typically have higher melatonin levels than patients with obesity or controls [99, 100]. Thus, melatonin might be a link between RIBEYE and the body weight. Measuring melatonin secretion in mutation carriers would thus be interesting.

In conclusion, we detected 24 variants located within RIBEYE’s A*-*domain. A subset of these were detected exclusively in patients with AN or obesity. Three NSVs (p.Val234Met, p.Tyr455His, p.Gln539Glu) were known to be associated with BMI. These, along with another SNP (p.Leu293Pro), are in high LD with two *CTBP2* SNPs previously associated with reduced BMI and increased AN-risk (rs12771627 and rs1561589), implying a potential functional effect, which should be further analyzed using site-directed mutagenesis or genome engineering technologies like CRISPR/Cas9. Our results indicate for the first time that RIBEYE could play a key role in weight regulation, possibly via its involvement in circadian mechanisms or its interaction with leptin. Further studies are needed to precisely characterize RIBEYE’s role in body weight regulation.

### URLs

Primer3 (http://bioinfo.ut.ee/primer3/)

ThermoFisher (http://www.thermofisher.com/de/de/home/life-science/pcr/real-time-pcr/real-time-pcr-assays/taqman-gene-expression.html)

NEBcutter V2.0 (http://nc2.neb.com/NEBcutter2/)

MutationTaster2 (http://www.mutationtaster.org/)

SIFT (http://sift.jcvi.org/www/SIFT_chr_coords_submit.html)

PolyPhen-2 (http://genetics.bwh.harvard.edu/pph2/)

SNAP (http://archive.broadinstitute.org/mpg/snap/ldsearchpw.php)

PredictSNP2 (https://loschmidt.chemi.muni.cz/predictsnp2/)

PROVEAN: http://provean.jcvi.org/index.php

HOPE: https://www3.cmbi.umcn.nl/hope/

MUPro: http://mupro.proteomics.ics.uci.edu/

I-Mutant 2.0: https://folding.biofold.org/i-mutant/i-mutant2.0.html

iStable 2.0: http://ncblab.nchu.edu.tw/iStable2/seqsubmit.html

TraP: http://trap-score.org/index.jsp

ESEfinder 3.0: http://krainer01.cshl.edu/cgi-bin/tools/ESE3/esefinder.cgi

Spliceman: http://fairbrother.biomed.brown.edu/spliceman/

SpliceAI: https://spliceailookup.broadinstitute.org/

dbSNP (https://www.ncbi.nlm.nih.gov/projects/SNP/)

CTG-VL: https://vl.genoma.io/updates

GWAS Catalog: https://www.ebi.ac.uk/gwas/home

### Software

DNASTAR Lasergene 11 (version11.0.0), GraphPad Prism (v9.4.1), R Studio for mac (2022.12.0+353).

## Supplementary information

Supplementary Material

## Data Availability

DNA sequences and raw data generated within this study are not openly available due to reasons of sensitivity and are only available from the corresponding author upon reasonable request.
