# Supplementary material for: Genetic and functional analyses of CTBP2 in anorexia nervosa and body weight regulation
Source: Mol Psychiatry. 2024 Nov 7;30(5):1836–46. doi: 10.1038/s41380-024-02791-3 (PMC12014503; doi:10.1038/s41380-024-02791-3)
Supplement: Supplementary file 1 — Supplementary Material [file 41380_2024_2791_MOESM1_ESM.pdf]

Supplementary Tables for Giuranna, Zheng et al. - Genetic and functional analyses of *CTBP2* in anorexia nervosa and body weight regulation

Supplementary Table 1: Sex-specific effect analyses on Pulit et al. [1] sex-stratified BMI GWAS datasets.

Supplementary Table 2: Used primer to amplify the coding regions of CTBP2.

Supplementary Table 3: SNPs located in the coding region of CTBP2 and detected in the mutation screen.

Supplementary Table 4: SNPs located in non-coding regions of CTBP2 and detected in the mutation screen.

Supplementary Table 5: List of genotyped non-synonymous SNPs and the detected genotype counts.

Supplementary Table 6: List of genotype counts of rare non-synonymous variants detected in the mutation screen and genotyping.

Supplementary Table 7: Detected variants and their respective frequencies in the European, non-Finnish population of gnomAD v3.1.2 [2].

Supplementary Table 8: Detected variants and their respective frequencies in the female European, non-Finnish population of gnomAD v3.1.2 [2].

Supplementary Table 9: Detected variants and their respective frequencies in the male European, non-Finnish population of gnomAD v3.1.2 [2].

Supplementary Table 10: Association testing with in-house control.

Supplementary Table 11: Association testing with an European, non-Finnish control population extracted from gnomAD v3.1.2 [2].

Supplementary Table 12: Analyzed species and sequences extracted from Ensembl (https://www.ensembl.org/index.html).

Supplementary Table 13: Conservation analyses.

Supplementary Table 14: Look-up of detected variants in GWAS for BMI [1].

Supplementary Table 15: Look-up of detected variants in GWAS for AN [3].

Supplementary Table 16: Overall predictions of all detected variants in RIBEYE/CTBP2 via MutationTaster2, CADD, PredicSNP2.

Supplementary Table 17: Prediction of the position of amino acid deleteriousness via PolyPhen2.0, PROVEAN, SIFT, and HOPE.

Supplementary Table 18: The prediction of the impact of NSVs on the alteration of RIBEYE protein stability via MUpro, iStable2.0, and I-Mutant2.0.

Supplementary Table 19: The predictions of SVs via TraP.

Supplementary Table 20: The possible effects of SVs on the pre-mRNA alternative splicing pattern via ESEfinder3.0, Spliceman and SpliceAI.

Supplementary Table 21: LD analysis for detected variants and three Hinney et al. 2017 AN and BMI relevant SNPs [20].

Supplementary Table 22: R^2 values of LD analysis for detected variants and genome-wide significant Pulit et al. BMI relevant SNPs [1].

Supplementary Table 23: D' values of LD analysis for detected variants and genome-wide significant Pulit et al. BMI relevant SNPs [1].

Supplementary Figure 1: Part of the Ribeye mRNA-sequence showing the primer binding sites for the nested PCR.

Supplementary Figure 2: Plots of chromosomal location and p values of variants located in CTBP2 and ± 500kb adjacent regions based on BMI and AN GWAS data.

Supplementary Figure 3: Sequence distances between human and 40 species from five superorders in protein level.

Supplementary Figure 4: Sequence distances between human and 40 species from five superorders in mRNA level.

Supplementary Figure 5: Analysis of Ribeye expression in murine hypothalamus by two-step PCR.

Supplementary Figure 6: Immunoblotting of RIBEYE expression in murine hypothalamus and retina.

Supplementary Table 1: Sex-specific effect analyses on Pulit et al. [1] sex-stratified BMI GWAS datasets.

Significant variant[1] information					Combined sexes				Females				Males				Effect size comparison	
SNP	CHR	POS	EA	NEA	FRQ	Beta	SE	P	FRQ	Beta	SE	P	FRQ	Beta	SE	P	Z-score	|Z-score|
rs1561589	10	126695673	A	G	0.362	0.014	0.002	1.04E-15	0.361	0.016	0.002	4.24E-12	0.360	0.011	0.002	1.87E-05	1.594	1.594
rs11245456	10	126684074	G	C	0.263	0.015	0.002	6.10E-16	0.262	0.017	0.003	5.98E-12	0.261	0.012	0.003	7.15E-06	1.386	1.386
rs17636031	10	126594078	C	T	0.276	0.015	0.002	3.87E-17	0.275	0.017	0.003	1.42E-11	0.275	0.013	0.003	7.48E-07	0.951	0.951
rs3012066	10	126737997	C	A	0.314	0.013	0.002	1.58E-13	0.312	0.016	0.002	5.75E-11	0.310	0.009	0.003	4.53E-04	1.894	1.894
rs2949371	10	126733978	A	G	0.455	-0.011	0.002	4.64E-12	0.456	-0.014	0.002	1.89E-10	0.457	-0.008	0.002	6.98E-04	-1.812	1.812
rs2936546	10	126731736	G	A	0.461	-0.012	0.002	1.57E-12	0.462	-0.014	0.002	3.83E-10	0.462	-0.009	0.003	3.70E-04	-1.619	1.619
rs3781446	10	126684134	T	C	0.455	-0.013	0.002	2.64E-14	0.455	-0.015	0.002	4.29E-10	0.456	-0.011	0.003	8.21E-06	-0.922	0.922
rs11245453	10	126683766	A	G	0.270	0.015	0.002	1.24E-14	0.269	0.016	0.003	1.06E-09	0.270	0.013	0.003	9.12E-06	0.837	0.837
rs12771627	10	126691180	A	G	0.273	0.013	0.002	8.10E-13	0.272	0.015	0.002	1.44E-09	0.272	0.011	0.003	6.04E-05	1.187	1.187
rs12220302	10	126607739	A	G	0.452	0.012	0.002	4.53E-10	0.453	0.016	0.003	1.48E-09	0.451	0.008	0.003	7.52E-03	2.105	2.105
rs2938002	10	126719360	G	C	0.473	-0.012	0.002	3.57E-12	0.474	-0.014	0.002	2.50E-09	0.475	-0.009	0.003	2.41E-04	-1.325	1.325
rs2949369	10	126719704	T	C	0.473	-0.012	0.002	3.61E-12	0.474	-0.014	0.002	2.59E-09	0.475	-0.009	0.003	2.39E-04	-1.325	1.325
rs2913112	10	126720673	A	G	0.473	-0.012	0.002	3.38E-12	0.474	-0.014	0.002	2.66E-09	0.474	-0.009	0.003	2.11E-04	-1.295	1.295
rs2949368	10	126726080	T	C	0.464	-0.012	0.002	1.01E-11	0.465	-0.014	0.002	2.80E-09	0.466	-0.009	0.003	4.25E-04	-1.413	1.413
rs72828935	10	126587488	C	G	0.277	0.016	0.002	1.42E-14	0.277	0.017	0.003	3.47E-09	0.278	0.015	0.003	6.21E-06	0.648	0.648
rs2938005	10	126716847	T	C	0.471	-0.012	0.002	2.54E-11	0.473	-0.014	0.002	3.55E-09	0.472	-0.008	0.003	1.16E-03	-1.554	1.554
rs2938004	10	126718350	G	A	0.472	-0.012	0.002	1.00E-11	0.473	-0.013	0.002	3.81E-09	0.473	-0.009	0.003	3.95E-04	-1.354	1.354
rs2919286	10	126719576	T	C	0.473	-0.012	0.002	8.86E-12	0.474	-0.014	0.002	3.90E-09	0.474	-0.009	0.003	3.71E-04	-1.354	1.354
rs11245446	10	126650696	G	A	0.320	0.013	0.002	1.36E-12	0.318	0.015	0.003	3.99E-09	0.319	0.011	0.003	5.76E-05	0.978	0.978
rs2935653	10	126730491	C	T	0.467	-0.012	0.002	6.34E-12	0.468	-0.014	0.002	4.32E-09	0.469	-0.009	0.003	2.46E-04	-1.266	1.266
rs11245458	10	126685344	T	C	0.268	0.015	0.002	5.62E-14	0.266	0.015	0.003	4.87E-09	0.267	0.012	0.003	1.39E-05	0.719	0.719
rs3012067	10	126716870	G	T	0.469	-0.012	0.002	6.85E-12	0.471	-0.013	0.002	5.31E-09	0.472	-0.009	0.003	1.85E-04	-1.177	1.177

rs2937999	10	126726335	C	G	0.462	-0.011	0.002	1.54E-11	0.463	-0.013	0.002	7.19E-09	0.464	-0.009	0.003	2.27E-04	-1.177	1.177
rs11245455	10	126683857	T	C	0.265	0.014	0.002	1.65E-13	0.263	0.015	0.003	7.38E-09	0.264	0.012	0.003	1.36E-05	0.693	0.693
rs2949372	10	126734115	A	G	0.444	-0.012	0.002	1.78E-10	0.443	-0.015	0.003	7.65E-09	0.444	-0.009	0.003	2.83E-03	-1.695	1.695
rs67609008	10	126640936	C	T	0.278	0.016	0.002	2.75E-14	0.278	0.017	0.003	8.13E-09	0.279	0.014	0.003	8.53E-06	0.602	0.602
rs3012074	10	126722535	G	A	0.469	-0.011	0.002	1.66E-11	0.470	-0.013	0.002	8.14E-09	0.471	-0.009	0.003	2.33E-04	-1.177	1.177
rs11245450	10	126658075	A	G	0.413	-0.012	0.002	2.04E-11	0.411	-0.014	0.002	8.62E-09	0.412	-0.009	0.003	3.88E-04	-1.268	1.268
rs2043953	10	126721954	A	G	0.469	-0.011	0.002	1.67E-11	0.470	-0.013	0.002	9.33E-09	0.471	-0.009	0.003	1.99E-04	-1.119	1.119
rs3781396	10	126725553	G	C	0.319	0.013	0.002	5.04E-13	0.319	0.014	0.003	9.54E-09	0.319	0.012	0.003	8.95E-06	0.598	0.598
rs2938001	10	126720288	T	C	0.469	-0.011	0.002	1.84E-11	0.470	-0.013	0.002	1.06E-08	0.471	-0.009	0.003	2.31E-04	-1.148	1.148
rs2913111	10	126722494	A	G	0.463	-0.012	0.002	1.68E-10	0.463	-0.015	0.003	1.10E-08	0.463	-0.009	0.003	3.28E-03	-1.669	1.669
rs2938006	10	126716346	T	C	0.490	-0.011	0.002	1.26E-10	0.491	-0.013	0.002	1.30E-08	0.491	-0.008	0.003	2.13E-03	-1.560	1.560
rs2946994	10	126714714	C	G	0.489	-0.011	0.002	9.98E-11	0.489	-0.013	0.002	1.38E-08	0.489	-0.008	0.003	1.43E-03	-1.472	1.472
rs11245481	10	126732558	A	G	0.272	0.013	0.002	2.37E-12	0.271	0.015	0.003	1.44E-08	0.272	0.010	0.003	2.01E-04	1.099	1.099
rs4962424	10	126730214	A	T	0.322	0.013	0.002	3.21E-13	0.322	0.014	0.003	1.73E-08	0.322	0.012	0.003	3.69E-06	0.408	0.408
rs2946993	10	126728403	T	C	0.462	-0.011	0.002	4.87E-11	0.463	-0.013	0.002	1.75E-08	0.464	-0.009	0.003	4.01E-04	-1.207	1.207
rs2913113	10	126719833	A	G	0.462	-0.012	0.002	4.25E-10	0.462	-0.015	0.003	1.87E-08	0.462	-0.008	0.003	5.11E-03	-1.720	1.720
rs12357688	10	126711516	T	C	0.264	0.013	0.002	8.90E-12	0.263	0.015	0.003	2.08E-08	0.263	0.010	0.003	4.47E-04	1.207	1.207
rs10901851	10	126722650	C	A	0.264	0.013	0.002	4.20E-12	0.263	0.015	0.003	2.09E-08	0.264	0.011	0.003	1.84E-04	1.021	1.021
rs10901854	10	126733643	C	T	0.276	0.013	0.002	2.30E-12	0.276	0.014	0.003	2.14E-08	0.276	0.011	0.003	1.18E-04	0.942	0.942
rs2363893	10	126714200	T	C	0.490	-0.011	0.002	9.88E-11	0.490	-0.013	0.002	2.41E-08	0.491	-0.008	0.003	9.65E-04	-1.295	1.295
rs4962421	10	126724927	A	G	0.268	0.014	0.002	1.94E-12	0.268	0.014	0.003	2.49E-08	0.268	0.011	0.003	9.70E-05	0.890	0.890
rs3012075	10	126714966	G	A	0.489	-0.011	0.002	2.66E-10	0.490	-0.013	0.002	2.61E-08	0.490	-0.008	0.003	1.56E-03	-1.413	1.413
rs11245478	10	126724796	C	G	0.268	0.014	0.002	1.64E-12	0.267	0.014	0.003	2.66E-08	0.268	0.011	0.003	7.12E-05	0.837	0.837
rs3781409	10	126715629	T	C	0.267	0.013	0.002	1.74E-11	0.266	0.014	0.003	3.11E-08	0.267	0.010	0.003	3.95E-04	1.125	1.125
rs10901852	10	126728522	C	G	0.267	0.013	0.002	7.62E-12	0.267	0.014	0.003	3.16E-08	0.267	0.010	0.003	2.58E-04	1.021	1.021
rs7086797	10	126711691	T	C	0.294	0.013	0.002	5.11E-12	0.294	0.014	0.003	3.37E-08	0.294	0.011	0.003	4.22E-05	0.734	0.734
rs4962725	10	126733321	C	T	0.431	0.013	0.002	8.47E-11	0.431	0.015	0.003	3.40E-08	0.431	0.010	0.003	5.68E-04	1.161	1.161

rs2839737	10	126721441	A	C	0.458	-0.012	0.002	7.96E-10	0.458	-0.014	0.003	3.74E-08	0.459	-0.008	0.003	4.01E-03	-1.566	1.566
rs11245479	10	126728195	T	C	0.269	0.013	0.002	1.03E-11	0.269	0.014	0.003	3.86E-08	0.270	0.010	0.003	2.93E-04	1.047	1.047
rs11245469	10	126713807	T	C	0.268	0.013	0.002	1.53E-11	0.267	0.014	0.003	4.38E-08	0.268	0.010	0.003	3.67E-04	1.047	1.047
rs1561586	10	126721685	G	A	0.463	-0.012	0.002	6.97E-10	0.463	-0.014	0.003	4.47E-08	0.463	-0.008	0.003	3.51E-03	-1.540	1.540
rs3781428	10	126702843	T	C	0.264	0.013	0.002	1.75E-11	0.263	0.014	0.003	4.77E-08	0.264	0.010	0.003	5.93E-04	1.178	1.178
rs35186116	10	126582160	G	A	0.286	0.015	0.002	2.77E-13	0.286	0.016	0.003	5.20E-08	0.287	0.014	0.003	8.25E-06	0.370	0.370
rs4962723	10	126705111	T	C	0.268	0.013	0.002	2.91E-11	0.268	0.014	0.003	5.43E-08	0.268	0.010	0.003	6.02E-04	1.125	1.125
rs4962708	10	126617169	C	T	0.181	0.014	0.002	1.72E-09	0.180	0.017	0.003	6.75E-08	0.180	0.010	0.003	4.14E-03	1.521	1.521
rs11245472	10	126719263	C	G	0.266	0.013	0.002	1.18E-11	0.265	0.014	0.003	6.93E-08	0.266	0.011	0.003	2.20E-04	0.942	0.942
rs11245462	10	126697962	G	A	0.269	0.013	0.002	1.86E-11	0.269	0.014	0.003	7.97E-08	0.268	0.010	0.003	3.18E-04	0.968	0.968
rs7073257	10	126711633	A	G	0.268	0.013	0.002	2.73E-11	0.267	0.014	0.003	8.34E-08	0.267	0.010	0.003	4.11E-04	1.021	1.021
rs3781415	10	126712615	T	C	0.486	-0.012	0.002	1.76E-09	0.486	-0.014	0.003	8.40E-08	0.486	-0.008	0.003	7.95E-03	-1.643	1.643
rs2018366	10	126697609	C	T	0.266	0.013	0.002	2.71E-11	0.265	0.014	0.003	8.65E-08	0.265	0.010	0.003	4.09E-04	1.021	1.021
rs893857	10	126720788	A	G	0.261	0.013	0.002	1.07E-11	0.260	0.014	0.003	9.04E-08	0.260	0.011	0.003	1.70E-04	0.873	0.873
rs12414708	10	126685916	A	G	0.452	-0.013	0.002	1.16E-11	0.452	-0.014	0.003	9.59E-08	0.452	-0.012	0.003	3.22E-05	-0.530	0.530
rs3781426	10	126703349	T	C	0.267	0.013	0.002	2.63E-11	0.267	0.014	0.003	9.90E-08	0.267	0.010	0.003	3.16E-04	0.942	0.942
rs4109292	10	126710654	A	G	0.488	-0.012	0.002	1.34E-09	0.488	-0.014	0.003	1.03E-07	0.488	-0.008	0.003	4.90E-03	-1.489	1.489
rs4962709	10	126621489	A	G	0.185	0.013	0.002	5.52E-09	0.184	0.016	0.003	1.08E-07	0.185	0.009	0.003	6.40E-03	1.565	1.565
rs12769019	10	126697327	G	A	0.276	0.012	0.002	5.51E-11	0.274	0.014	0.003	1.29E-07	0.275	0.011	0.003	1.75E-04	0.785	0.785
rs4411245	10	126712781	A	G	0.296	0.013	0.002	1.22E-11	0.296	0.013	0.003	1.32E-07	0.296	0.011	0.003	4.16E-05	0.571	0.571
rs4962416	10	126696872	C	T	0.276	0.012	0.002	5.60E-11	0.274	0.013	0.003	1.40E-07	0.275	0.011	0.003	1.55E-04	0.746	0.746
rs3012065	10	126737579	C	T	0.330	0.013	0.002	3.83E-10	0.330	0.015	0.003	2.16E-07	0.330	0.009	0.003	2.80E-03	1.269	1.269
rs3781445	10	126688370	A	G	0.407	-0.012	0.002	1.08E-11	0.409	-0.012	0.002	2.21E-07	0.410	-0.011	0.003	1.63E-05	-0.339	0.339
rs3781433	10	126695847	G	A	0.480	0.011	0.002	3.87E-10	0.479	0.012	0.002	2.56E-07	0.479	0.009	0.003	4.24E-04	0.883	0.883
rs12782469	10	126688200	T	C	0.271	0.013	0.002	7.04E-11	0.269	0.013	0.003	4.31E-07	0.269	0.011	0.003	7.97E-05	0.523	0.523
rs2946996	10	126738444	T	C	0.390	0.012	0.002	4.66E-10	0.390	0.014	0.003	4.43E-07	0.389	0.010	0.003	4.30E-04	0.808	0.808
rs11245454	10	126683802	A	G	0.278	0.014	0.002	1.04E-11	0.278	0.015	0.003	4.72E-07	0.279	0.013	0.003	3.70E-05	0.347	0.347

rs2028395	10	126726558	C	G	0.443	0.011	0.002	2.79E-10	0.443	0.012	0.002	6.13E-07	0.442	0.010	0.003	1.70E-04	0.618	0.618
rs2919290	10	126738533	A	T	0.495	0.011	0.002	1.66E-08	0.495	0.013	0.003	7.00E-07	0.494	0.008	0.003	4.23E-03	1.232	1.232
rs2028398	10	126696643	G	A	0.373	0.013	0.002	7.08E-11	0.373	0.013	0.003	7.24E-07	0.373	0.011	0.003	1.31E-04	0.496	0.496
rs2028397	10	126696695	G	A	0.372	0.013	0.002	9.98E-11	0.372	0.013	0.003	7.61E-07	0.372	0.011	0.003	1.69E-04	0.545	0.545
rs7923776	10	126690383	C	T	0.482	0.011	0.002	3.73E-10	0.481	0.011	0.002	7.86E-07	0.480	0.010	0.003	1.19E-04	0.500	0.500
rs4962719	10	126696704	G	A	0.372	0.013	0.002	1.05E-10	0.372	0.013	0.003	8.27E-07	0.372	0.011	0.003	1.65E-04	0.545	0.545
rs3781444	10	126688968	G	A	0.481	0.011	0.002	3.63E-10	0.479	0.012	0.002	8.85E-07	0.479	0.010	0.003	1.27E-04	0.452	0.452
rs1970811	10	126696496	C	T	0.453	0.010	0.002	2.55E-09	0.451	0.011	0.002	9.75E-07	0.452	0.008	0.003	1.79E-03	1.001	1.001
rs3781422	10	126708783	T	A	0.487	0.011	0.002	3.18E-09	0.487	0.013	0.003	1.03E-06	0.487	0.009	0.003	1.80E-03	0.976	0.976
rs558740521	10	126718074	C	T	0.487	0.011	0.002	4.99E-09	0.487	0.013	0.003	1.99E-06	0.487	0.009	0.003	1.99E-03	0.924	0.924
rs718947	10	126739149	G	A	0.358	0.012	0.002	6.11E-11	0.358	0.011	0.002	2.29E-06	0.359	0.012	0.003	2.33E-06	-0.231	0.231
rs2289431	10	126727353	A	C	0.320	0.013	0.002	9.66E-10	0.320	0.013	0.003	3.87E-06	0.320	0.012	0.003	1.79E-04	0.359	0.359
rs75011412	10	126616820	T	C	0.051	-0.027	0.005	3.02E-09	0.051	-0.028	0.006	5.11E-06	0.052	-0.031	0.007	3.30E-06	0.340	0.340
rs4962724	10	126733319	G	A	0.278	0.013	0.002	1.76E-09	0.278	0.013	0.003	5.11E-06	0.279	0.011	0.003	8.70E-04	0.625	0.625
rs3781442	10	126690919	G	A	0.489	0.011	0.002	1.05E-08	0.489	0.012	0.003	6.43E-06	0.489	0.010	0.003	8.49E-04	0.565	0.565
rs11245480	10	126728286	G	C	0.323	0.012	0.002	3.76E-09	0.323	0.013	0.003	6.79E-06	0.323	0.011	0.003	3.44E-04	0.407	0.407
rs4962422	10	126728880	T	C	0.270	0.013	0.002	2.94E-09	0.270	0.013	0.003	7.11E-06	0.271	0.011	0.003	1.12E-03	0.638	0.638
rs7923382	10	126576503	C	T	0.226	-0.012	0.002	4.08E-09	0.226	-0.012	0.003	7.52E-06	0.226	-0.014	0.003	2.01E-06	0.496	0.496
rs3781395	10	126726827	T	C	0.270	0.013	0.002	2.35E-09	0.270	0.013	0.003	8.33E-06	0.270	0.011	0.003	8.70E-04	0.547	0.547
rs3781421	10	126709703	T	C	0.265	0.013	0.002	1.62E-09	0.265	0.013	0.003	8.84E-06	0.266	0.011	0.003	9.95E-04	0.561	0.561
rs11245405	10	126572654	C	T	0.221	-0.012	0.002	6.31E-09	0.221	-0.012	0.003	1.04E-05	0.221	-0.014	0.003	2.38E-06	0.512	0.512
rs10444192	10	126714166	T	C	0.270	0.012	0.002	1.00E-08	0.269	0.013	0.003	1.08E-05	0.270	0.010	0.003	2.85E-03	0.775	0.775
rs11245406	10	126572672	C	T	0.221	-0.012	0.002	6.80E-09	0.221	-0.012	0.003	1.14E-05	0.221	-0.014	0.003	2.29E-06	0.536	0.536
rs11245407	10	126572693	C	T	0.221	-0.012	0.002	7.33E-09	0.221	-0.012	0.003	1.23E-05	0.221	-0.014	0.003	2.19E-06	0.560	0.560
rs12355063	10	126572860	C	G	0.221	-0.012	0.002	8.39E-09	0.221	-0.012	0.003	1.24E-05	0.222	-0.014	0.003	2.82E-06	0.512	0.512
rs12247671	10	126575210	A	G	0.223	-0.012	0.002	1.04E-08	0.223	-0.012	0.003	1.33E-05	0.223	-0.014	0.003	2.73E-06	0.512	0.512
rs76497870	10	126679432	A	G	0.054	-0.026	0.004	4.36E-09	0.053	-0.026	0.006	1.39E-05	0.054	-0.032	0.007	1.42E-06	0.605	0.605

rs4962420	10	126710653	T	C	0.271	0.012	0.002	8.62E-09	0.271	0.013	0.003	1.41E-05	0.271	0.010	0.003	2.29E-03	0.684	0.684
rs10901833	10	126572728	G	A	0.229	-0.012	0.002	8.19E-09	0.229	-0.012	0.003	1.42E-05	0.230	-0.014	0.003	2.05E-06	0.585	0.585
rs12769682	10	126697494	C	G	0.286	0.012	0.002	1.78E-08	0.285	0.013	0.003	1.57E-05	0.286	0.010	0.003	1.11E-03	0.486	0.486
rs3781416	10	126712428	A	G	0.270	0.012	0.002	7.61E-09	0.270	0.013	0.003	1.70E-05	0.271	0.010	0.003	1.66E-03	0.570	0.570
rs3781424	10	126704700	C	T	0.267	0.012	0.002	1.68E-08	0.267	0.013	0.003	1.94E-05	0.267	0.010	0.003	3.24E-03	0.707	0.707
rs75274506	10	126600778	C	T	0.054	-0.025	0.004	1.19E-08	0.053	-0.026	0.006	1.96E-05	0.054	-0.030	0.007	5.12E-06	0.463	0.463
rs4962419	10	126697114	A	G	0.286	0.012	0.002	2.61E-08	0.286	0.012	0.003	2.12E-05	0.287	0.010	0.003	1.11E-03	0.440	0.440
rs4962720	10	126696840	T	G	0.286	0.012	0.002	2.59E-08	0.286	0.012	0.003	2.21E-05	0.287	0.010	0.003	1.07E-03	0.440	0.440
rs718948	10	126739338	C	T	0.357	0.010	0.002	2.74E-09	0.358	0.010	0.002	3.53E-05	0.358	0.011	0.003	1.82E-05	-0.353	0.353
rs60306160	10	126703761	T	C	0.270	0.012	0.002	2.93E-08	0.270	0.012	0.003	4.19E-05	0.270	0.010	0.003	2.47E-03	0.524	0.524
rs78159028	10	126675765	C	G	0.048	-0.026	0.005	3.74E-08	0.048	-0.025	0.006	7.21E-05	0.048	-0.032	0.007	3.48E-06	0.727	0.727
rs11598549	10	126689249	A	G	0.273	0.012	0.002	1.96E-08	0.273	0.012	0.003	8.32E-05	0.274	0.012	0.003	2.99E-04	-0.023	0.023

[1]Significant variant: variant significant in at least one Pulit et al. BMI GWAS datasets (combined sexes, females and males) (1). CHR: Chromosome. POS: chromosomal position (GRCH37). EA: effect allele. NEA: non-effect allele. FRQ: frequency of effect allele. Beta: effect size. SE: standard error of effect size. P: P-value.

Supplementary Table 2: Used primer to amplify the coding regions of *CTBP2*.

Transcript	Exon	Primer name	Sequence (5' -> 3')	Amplicon size
CTBP2	specific exon for CTBP2-L	F	AGTCCCCAGTGAGCTGTTGTC	290
		R	GCGAAGATTTGAGTGAGGGA	
RIBEYE	specific exon for RIBEYE (A-domain)	Fragment 1 - F	TAATCCGCATGCTGAGAGAG	866
		Fragment 1 - R	GGGATCATTTTACCTCCAG	
		Fragment 2 - F	GTCTACTCCTGACTTCACCTTCT	639
		Fragment 2 - R	AGAAAGGCCAGGAACTCAG	
		Fragment 3 - F	ATCCCATCCAAGATGGCTTA	990
		Fragment 3 - R	GGTGAGTGAGGACTTGCCAT	
CTBP2/RIBEYE	B-domain exon 2 & 3	F	CCTGAGTGATGAGTGGGGAG	893 bp
		R	AGAGCTCCGGACAACTCCAG	
	B-domain exon 4	F	GCTTCCCCACTGATCTTGAG	483 bp
		R	ACACGCATTCTTCCAGAACC	
	B-domain exon 5 & 6	F	AGCTCAGAAGCAATGGAGGT	990 bp
		R	AAGGGCCTCGAGTTCAAAGT	
	B-domain exon 7 & 8	F	AAGAGCTCCCTTGTGCTGAG	755 bp
		R	TGGTGCTCACAAGCTGAGAC	
	B-domain exon 9	F	CCAGAAGTAGAAGAGCCAAG	491 bp
		R	ACACACAATAACAGAAGTTGG	

Supplementary Table 3: SNPs located in the coding region of *CTBP2* and detected in the mutation screen.

SNP ID	Genomic position (GRCh38)	Location in CTBP2	Ref allele	Alt allele	AA alteration	Female patients with AN				Children and adolescents with obesity											
										Male				Female				All			
						11	12	22	MAF (%)	11	12	22	MAF (%)	11	12	22	MAF (%)	11	12	22	MAF (%)
rs150867595	chr10: 125027603	A-domain	G	A	Pro53Ser	95	0	0	0	43	1	0	1.14	48	0	0	0	91	1	0	0.54
rs146900874	chr10:125027546	A-domain	G	A	Arg72Trp	94	1	0	0.53	44	0	0	0	48	0	0	0	92	0	0	0
rs141864737	chr10:125027235-125027236	A-domain	CT	-	Gln175fs	95	0	0	0	43	1	0	1.14	48	0	0	0	91	1	0	0.54
rs3781407	chr10:125027175	A-domain	G	A	Asp195=	88	6	1	4.21	40	4	0	4.55	45	3	0	3.13	85	7	0	3.8
rs116403181	chr10:125027124	A-domain	G	A	Ser212=	95	0	0	0	44	0	0	0	47	1	0	1.04	91	1	0	0.54
rs3781408	chr10:125027123	A-domain	C	T	Asp213Asn	84	11	0	5.79	40	4	0	4.55	41	7	0	7.29	81	11	0	5.98
rs3781409	chr10:125027060	A-domain	C	T	Val234Met	50	41	4	25.8	20	19	5	33	25	21	2	26	45	40	7	29.3
rs45440394	chr10:125026899	A-domain	C	T	Arg287=	93	2	0	1.05	44	0	0	0	46	2	0	2.08	90	2	0	1.09
rs375685611	chr10:125026895	A-domain	C	T	Val289Met	94	1	0	0.53	44	0	0	0	48	0	0	0	92	0	0	0
rs3781411	chr10:125026867	A-domain	C	T	Arg298Gln	72	22	1	12.6	34	9	1	12.5	40	8	0	8.33	74	17	1	10.3
rs769811964	chr10:125026832	A-domain	G	C	Leu310Val	95	0	0	0	44	0	0	0	47	1	0	1.04	91	1	0	0.54
rs142101185	chr10:125026767	A-domain	A	C	Ser331=	94	1	0	0.53	44	0	0	0	47	1	0	1.04	91	1	0	0.54
rs202010294	chr10:125026676	A-domain	C	G	Gly362Arg	94	1	0	0.53	44	0	0	0	48	0	0	0	92	0	0	0
rs45535234	chr10:125026632	A-domain	G	A	Ser376=	92	3	0	1.58	43	1	0	1.14	48	0	0	0	91	1	0	0.54
rs535621897	chr10:125026629	A-domain	T	A	Glu377Asp	94	1	0	0.53	43	1	0	1.14	48	0	0	0	91	1	0	0.54
rs372118432	chr10:125026591-125026597	A-domain	-	insCT(G)4CTGCAGGGCCGCAG	Pro391_Leu392Ins	72	22	1	12.6	34	9	1	12.5	40	8	0	8.33	74	17	1	10.3

rs3781412	chr10:125026585	A-domain	A	G	Leu392Pro	33	51	11	38.4	13	22	9	45.5	20	23	5	34.4	33	45	14	39.7
rs76134089	chr10:125026571	A-domain	G	C	Pro397Ala	95	0	0	0	44	0	0	0	47	1	0	1.04	91	1	0	0.54
rs113477585	chr10:125026556	A-domain	G	A	Pro402Ser	95	0	0	0	44	0	0	0	47	1	0	1.04	91	1	0	0.54
rs3781413	chr10:125026506	A-domain	T	C	Ala418=	72	22	1	12.6	34	9	1	12.5	40	8	0	8.33	74	17	1	10.3
rs3012075	chr10:125026397	A-domain	A	G	Tyr455His	21	48	26	52.6	16	20	8	40.9	9	27	12	46.9	25	47	20	47.2
rs894087529	chr10:125026362	A-domain	C	G	Pro466=	95	0	0	0	44	0	0	0	47	1	0	1.04	91	1	0	0.54
rs2946994	chr10:125026145	A-domain	G	C	Gln539Glu	21	48	26	52.6	16	20	8	40.9	9	27	12	46.9	25	47	20	47.3

All variants fulfilled the HWE. AA: amino acid. MAF: minor allele frequency. 11: homozygous reference allele. 12: heterozygous. 22: homozygous alternative allele.

Supplementary Table 4: SNPs located in non-coding regions of *CTBP2* and detected in the mutation screen.

SNP ID	Genomic position (GRCh38)	Location in *CTBP2*	Ref allele	Alt allele	Female patients with AN				Children and adolescents with obesity											
									Male				Female				All			
					11	12	22	MAF (%)	11	12	22	MAF (%)	11	12	22	MAF (%)	11	12	22	MAF (%)
rs61870287	chr10: 124989333	3' UTR	C	T	95	0	0	0	43	1	0	1.14	48	0	0	0	91	1	0	0.54
rs78563051	chr10: 124989434	3' UTR	C	A	95	0	0	0	43	1	0	1.14	48	0	0	0	91	1	0	0.54
rs7097802	chr10: 124993119	intronic	T	C	81	14	0	7.4	39	4	0	4.7	43	6	0	6.2	82	10	0	5.4
rs543711955	chr10: 124993120	intronic	G	A	95	0	0	0	44	0	0	0	47	1	0	1.04	91	1	0	0.54
rs111768849	chr10: 124994064	intronic	C	T	95	0	0	0	44	0	0	0	47	1	0	1.04	91	1	0	0.54
rs548203435	chr10: 124994128	intronic	G	A	95	0	0	0	44	0	0	0	47	1	0	1.04	91	1	0	0.54
rs72828988	chr10: 124997821	intronic	A	G	95	0	0	0	43	1	0	1.14	47	1	0	1.04	90	2	0	1.09
rs72828989	chr10: 124998193	intronic	C	T	81	12	2	8.42	36	7	1	10.2	39	8	1	10.42	75	15	2	10.3
rs4348846	chr10: 125002902	intronic	G	A	62	32	1	17.9	33	10	1	13.6	36	12	0	12.5	69	22	1	13
rs376708829	chr10: 125002929	intronic	C	T	94	1	0	0.53	44	0	0	0	48	0	0	0	92	0	0	0
rs750600	chr10: 125003497	intronic	C	A	84	10	1	6.32	38	5	1	7.95	41	7	0	7.29	79	12	1	7.61
rs567213936	chr10: 125003548	intronic	G	A	95	0	0	0	44	0	0	0	47	1	0	1.04	91	1	0	0.54
rs182939420	chr10: 125003554	intronic	G	C	95	0	0	0	43	1	0	1.14	48	0	0	0	91	1	0	0.54
rs750599	chr10: 125003624	intronic	C	T	29	47	19	44.7	14	20	10	45.5	13	28	7	43.8	27	48	17	44.6
rs12571821	chr10: 125026072	intronic	G	C	89	6	0	3.16	41	3	0	3.41	44	4	0	4.17	85	7	0	3.8
rs2938006	chr10: 125027777	5' UTR	C	T	21	48	26	47.4	16	20	8	40.9	8	28	12	45.8	24	48	20	47.8
rs76319950	chr10: 125039061	5' UTR	T	C	94	1	0	0.53	44	0	0	0	48	0	0	0	92	0	0	0

Due to the location of the primer binding sites in non-coding regions, certain parts of intronic and untranslated regions and in some cases complete introns were screened. MAF: minor allele frequency. 11: homozygous reference allele. 12: heterozygous. 22: homozygous alternative allele.

Supplementary Table 5: List of genotyped non-synonymous SNPs and the detected genotype counts.

SNP ID	Genomic position (GRCh38)	Ref allele	Alt allele	AA alteration	Method used	Patients with AN				Children and adolescents with severe obesity				Healthy-lean controls				Normal-weight controls			
						11	12	22	MAF (%)	11	12	22	MAF (%)	11	12	22	MAF (%)	11	12	22	MAF (%)
rs150867595	chr10: 125027603	G	A	Pro53Ser	RFLP	366	1	0	0.13	398	0	0	0	445	0	0	0	168	0	0	0
rs146900874	chr10: 125027546	G	A	Arg72Trp	TaqMan	367	0	0	0	398	0	0	0	444	1	0	0.11	168	0	0	0
rs137997200*	chr10: 125027235-125027236	(G)4A(CCGG)2CTGACT	-	Val132fs	RFLP*	367	0	0	0	397	1	0	0.13	445	0	0	0	168	0	0	0
rs141864737	chr10: 125027235-125027236	CT	-	Gln175fs	TaqMan	366	1	0	0.13	396	2	0	0.25	442	3	0	0.34	166	2	0	0.6
rs375685611	chr10: 125026895	C	T	Val289Met	RFLP	367	0	0	0	398	0	0	0	445	0	0	0	168	0	0	0
rs769811964	chr10: 125026832	G	C	Leu310Val	RFLP	367	0	0	0	398	0	0	0	445	0	0	0	168	0	0	0
rs202010294	chr10: 125026676	C	G	Gly362Arg	RFLP	364	3	0	0.41	396	2	0	0.25	443	2	0	0.22	165	3	0	0.89
rs76134089	chr10: 125026571	G	C	Pro397Ala	TaqMan	367	0	0	0	398	0	0	0	444	1	0	0.11	168	0	0	0
rs113477585	chr10: 125026556	G	A	Pro402Ser	TaqMan	367	0	0	0	398	0	0	0	445	0	0	0	168	0	0	0

*This SNP was detected in a RFLP approach for another SNP and was initially not identified in the preceding mutation screen. AA: amino acid. MAF: minor allele frequency. RFLP: restriction fragment length polymorphism.

Supplementary Table 6: List of genotype counts of rare non-synonymous variants detected in the mutation screen and genotyping.

**SNP ID**	**Genomic position (GRCh38)**	**Ref allele**	**Alt allele**	**AA alteration**	**Patients with AN**				**Children and adolescents with severe obesity**			
					**11**	**12**	**22**	**MAF (%)**	**11**	**12**	**22**	**MAF (%)**
rs150867595	chr10: 125027603	G	A	Pro53Ser	461	1	0	0.11	489	1	0	0.1
rs146900874	chr10: 125027546	G	A	Arg72Trp	461	1	0	0.11	490	0	0	0
rs137997200*	chr10: 125027235-125027236	(G)4A(CCGG)2 CTGACT	-	Val132*fs*	462	0	0	0	489	1	0	0.1
rs141864737	chr10: 125027235-125027236	CT	-	Gln175*fs*	461	1	0	0.11	487	3	0	0.31
rs375685611	chr10: 125026895	C	T	Val289Met	461	1	0	0.11	490	0	0	0
rs769811964	chr10: 125026832	G	C	Leu310Val	462	0	0	0	489	1	0	0.1
rs202010294	chr10: 125026676	C	G	Gly362Arg	458	4	0	0.43	488	2	0	0.2
rs76134089	chr10: 125026571	G	C	Pro397Ala	462	0	0	0	489	1	0	0.1
rs113477585	chr10: 125026556	G	A	Pro402Ser	462	0	0	0	489	1	0	0.1

AA: amino acid. MAF: minor allele frequency.

Supplementary Table 7: Detected variants and their respective frequencies in the European, non-Finnish population of gnomAD v3.1.2 (2).

SNP ID	Genomic position (GRCh38)	A1	A2	AA-alteration	Overall					
					A1 count	A2 count	A2 Freq(%)	A1 hom	het	A2 hom
rs2946994	chr10: 125026145	G	C	Gln539Glu	34248	33730	49.62	8628	16992	8369
rs894087529	chr10: 125026362	C	G	Pro466=	67986	0	0	33993	0	0
rs3012075	chr10: 125026397	A	G	Tyr455His	34105	33753	49.74	8573	16959	8397
rs3781413	chr10: 125026506	T	C	Ala418=	59051	8861	13.05	25670	7711	575
rs113477585	chr10: 125026556	G	A	Pro402Ser	64129	5	0.01	32062	5	0
rs76134089	chr10: 125026571	G	C	Pro397Ala	58126	16	0.03	29055	16	0
rs3781412	chr10: 125026585	A	G	Leu392Pro	41095	26825	39.49	12415	16265	5280
rs372118432	chr10: 125026591 - 125026597	-	*ins*CT(G)4CTGCAGGGCCGCAG	Pro391_leu392*ins*LeuGlnProGlnProAlaAla	59006	8840	13.03	25653	7700	570
rs535621897	chr10: 125026629	T	A	Glu377Asp	65640	46	0.07	32797	46	0
rs45535234	chr10: 125026632	G	A	Ser376=	66110	628	0.94	32747	616	6
rs202010294	chr10: 125026676	C	G	Gly362Arg	67800	170	0.25	33816	168	1
rs142101185	chr10: 125026767	A	C	Ser331=	67851	145	0.21	33853	145	0
rs769811964	chr10: 125026832	G	C	Leu310Val	68021	3	0	34009	3	0
rs3781411	chr10: 125026867	C	T	Arg298Gln	59107	8875	13.05	25689	7729	573
rs375685611	chr10: 125026895	C	T	Val289Met	68034	6	0.01	34014	6	0
rs45440394	chr10: 125026899	C	T	Arg287=	66966	1068	1.57	32960	1046	11
rs3781409	chr10: 125027060	C	T	Val234Met	50018	17968	26.43	18420	13178	2395
rs3781408	chr10: 125027123	C	T	Asp213Asn	64630	3390	4.98	30714	3202	94

rs116403181	chr10: 125027124	G	A	Ser212=	68031	5	0.01	34013	5	0
rs3781407	chr10: 125027175	G	A	Asp195=	66541	1493	2.19	32544	1453	20
rs141864737	chr10: 125027235 - 125027236	CT	-	Gln175*fs*	67821	205	0.30	33809	203	1
rs146900874	chr10: 125027546	G	A	Arg72Trp	68000	22	0.03	33989	22	0
rs150867595	chr10: 125027603	G	A	Pro53Ser	67963	57	0.08	33953	57	0
rs1379972000	chr10: 125027347-125027367	(G)4A(CCGG)2CTGACT	-	Val132*fs*	68012	2	0	34005	2	0

Allele counts were extracted from gnomAD's European, non-Finnish population (males and females) (2). A1: reference allele. A2: alternative allele. AA: amino acid. A2 Freq (%): frequency of alternative allele in percentage. N: sample size. hom: homozygous. het: heterozygous.

Supplementary Table 8: Detected variants and their respective frequencies in the female European, non-Finnish population of gnomAD v3.1.2 (2).

SNP ID	Genomic position (GRCh38)	A1	A2	AA-alteration	Female					
					A1 count	A2 count	A2 Freq(%)	A1 hom	het	A2 hom
rs2946994	chr10: 125026145	G	C	Gln539Glu	19749	19581	49.79	4970	9809	4886
rs894087529	chr10: 125026362	C	G	Pro466=	39328	0	0.00	19664	0	0
rs3012075	chr10: 125026397	A	G	Tyr455His	19661	19589	49.91	4936	9789	4900
rs3781413	chr10: 125026506	T	C	Ala418=	34181	5109	13.00	14853	4475	317
rs113477585	chr10: 125026556	G	A	Pro402Ser	37145	3	0.01	18571	3	0
rs76134089	chr10: 125026571	G	C	Pro397Ala	33577	7	0.02	16785	7	0
rs3781412	chr10: 125026585	A	G	Leu392Pro	23838	15470	39.36	7231	9376	3047
rs372118432	chr10: 125026591 - 125026597	-	*ins*CT(G)4CTGCAGGGCCGCAG	Pro391_Leu392*ins*LeuGlnProGlnProAlaAla	34176	5102	12.99	14853	4470	316
rs535621897	chr10: 125026629	T	A	Glu377Asp	38027	25	0.07	19001	25	0
rs45535234	chr10: 125026632	G	A	Ser376=	27865	287	1.02	13792	281	3
rs202010294	chr10: 125026676	C	G	Gly362Arg	39227	99	0.25	19564	99	0
rs142101185	chr10: 125026767	A	C	Ser331=	39260	84	0.21	19588	84	0
rs769811964	chr10: 125026832	G	C	Leu310Val	39359	1	0.00	19679	1	0
rs3781411	chr10: 125026867	C	T	Arg298Gln	34205	5119	13.02	14861	4483	318
rs375685611	chr10: 125026895	C	T	Val289Met	39358	4	0.01	19677	4	0
rs45440394	chr10: 125026899	C	T	Arg287=	38730	636	1.62	19054	622	7
rs3781409	chr10: 125027060	C	T	Val234Met	28975	10353	26.32	10694	7587	1383

rs3781408	chr10: 125027123	C	T	Asp213Asn	37441	1907	4.85	17816	1809	49
rs116403181	chr10: 125027124	G	A	Ser212=	39357	3	0.01	19677	3	0
rs3781407	chr10: 125027175	G	A	Asp195=	38496	862	2.19	18829	838	12
rs141864737	chr10: 125027235 - 125027236	CT	-	Gln175*fs*	39234	126	0.32	19555	124	1
rs146900874	chr10: 125027546	G	A	Arg72Trp	39345	9	0.02	19668	9	0
rs150867595	chr10: 125027603	G	A	Pro53Ser	39314	38	0.10	19638	38	0
rs1379972000	chr10: 125027347-125027367	(G)4A(CCGG)2C TGACT	-	Val132*fs*	39345	1	0.00	19672	1	0

Allele counts were extracted from gnomAD's female European, non-Finnish population (2). A1: reference allele. A2: alternative allele. AA: amino acid. A2 Freq (%): frequency of alternative allele in percentage. N: sample size. hom: homozygous. het: heterozygous.

Supplementary Table 9: Detected variants and their respective frequencies in the male European, non-Finnish population of gnomAD v3.1.2 (2).

SNP ID	Genomic position (GRCh38)	A1	A2	AA-alteration	Male					
					A1 count	A2 count	A2 Freq(%)	A1 hom	het	A2 hom
rs2946994	chr10: 125026145	G	C	Gln539Glu	14499	14149	49.39	3658	7183	3483
rs894087529	chr10: 125026362	C	G	Pro466=	28658	0	0.00	14329	0	0
rs3012075	chr10: 125026397	A	G	Tyr455His	14444	14164	49.51	3637	7170	3497
rs3781413	chr10: 125026506	T	C	Ala418=	24870	3752	13.11	10817	3236	258
rs113477585	chr10: 125026556	G	A	Pro402Ser	26984	2	0.01	13491	2	0
rs76134089	chr10: 125026571	G	C	Pro397Ala	24549	9	0.04	12270	9	0
rs3781412	chr10: 125026585	A	G	Leu392Pro	17257	11355	39.69	5184	6889	2233
rs372118432	chr10: 125026591 - 125026597	-	*ins*CT(G)4CTGCAGGGCCGCAG	Pro391_Leu392*ins*LeuGlnProGlnProAlaAla	24830	3738	13.08	10800	3230	254
rs535621897	chr10: 125026629	T	A	Glu377Asp	27613	21	0.08	13796	21	0
rs45535234	chr10: 125026632	G	A	Ser376=	38245	341	0.88	18955	335	3
rs202010294	chr10: 125026676	C	G	Gly362Arg	28573	71	0.25	14252	69	1
rs142101185	chr10: 125026767	A	C	Ser331=	28591	61	0.21	14265	61	0
rs769811964	chr10: 125026832	G	C	Leu310Val	28662	2	0.01	14330	2	0
rs3781411	chr10: 125026867	C	T	Arg298Gln	24902	3756	13.11	10828	3246	255
rs375685611	chr10: 125026895	C	T	Val289Met	28676	2	0.01	14337	2	0
rs45440394	chr10: 125026899	C	T	Arg287=	28236	432	1.51	13906	424	4
rs3781409	chr10: 125027060	C	T	Val234Met	21043	7615	26.57	7726	5591	1012

rs3781408	chr10: 125027123	C	T	Asp213Asn	27189	1483	5.17	12898	1393	45
rs116403181	chr10: 125027124	G	A	Ser212=	28674	2	0.01	14336	2	0
rs3781407	chr10: 125027175	G	A	Asp195=	28043	633	2.21	13713	617	8
rs141864737	chr10: 125027235 - 125027236	CT	-	Gln175*fs*	28587	79	0.28	14254	79	0
rs146900874	chr10: 125027546	G	A	Arg72Trp	28655	13	0.05	14321	13	0
rs150867595	chr10: 125027603	G	A	Pro53Ser	28649	19	0.07	14315	19	0
rs1379972000	chr10: 125027347- 125027367	(G)4A(CCGG)2C TGACT	-	Val132*fs*	28667	1	0.00	14333	1	0

Allele counts were extracted from gnomAD's male European, non-Finnish population (2). A1: reference allele. A2: alternative allele. AA: amino acid. A2 Freq (%): frequency of alternative allele in percentage. N: sample size. hom: homozygous. het: heterozygous.

Supplementary Table 10: Association testing with in-house control.

SNP ID	AA alteration	Obesity vs. healthy-lean controls				Obesity vs. Normal weight controls				Obesity (female) vs. Healthy-lean (female)				Obesity (female) vs. Normal-weight (female)				AN vs. Healthy-lean (female)			
		**Used test**	**p**	**95% CI**	**OR**	**Used test**	**p**	**95% CI**	**OR**	**Used test**	**p**	**95% CI**	**OR**	**Used test**	**p**	**95% CI**	**OR**	**Used test**	**p**	**95% CI**	**OR**
rs141864737	p.Gln175*fs*	Fisher	1	0.12-6.80	0.91	Fisher	0.61	0.06-6.17	0.51	Fisher	1	0.07-13.63	0.99	Fisher	0,12	0.02-2.12	0,24	NA	NA		
rs202010294*	p.Gly362Arg	NA	NA			NA	NA			Fisher	0.62	0.01-9.48	0.49	Fisher	0,18	0.003-3.51	0,18	Fisher	1	0.17-13.21	1.19

Only NSVs which were detected in our mutation screen and genotyping approach more than once in at least two study groups were considered. *The NSV was detected only in females. Thus, the statistical analyses were only performed in females. AA: amino acid. p: p-value. 95% CI: 95% confidence interval of odds ratio. OR: odds ratio. NA: not available/applicable.

Supplementary Table 11: Association testing with an European, non-Finnish control population extracted from gnomAD v3.1.2 (2).

SNP ID	AA alteration	Obesity vs. gnomAD (EU, non-Fin)				Obesity (female) vs. gnomAD (EU, non-Fin; female)				Obesity (male) vs. gnomAD (EU, non-Fin; male)				AN (female) vs. gnomAD (EU, non-Fin; female)			
		Used test	p	95% CI	OR	Used test	p	95% CI	OR	Used test	p	95% CI	OR	Used test	p	95% CI	OR
rs2946994	Gln539Glu	Chi-square	0.53	0.68-1.22	0.91	Chi-square	0.51	0.76-1.71	1.14	Chi-square	0.11	0.46-1.08	0.71	Chi-square	0.43	0.84-1.49	1.12
rs894087529	Pro466=	NA				NA				NA				NA			
rs3012075	Tyr455His	Chi-square	0.51	0.68-1.21	0.91	Chi-square	0.53	0.76-1.71	1.14	Chi-square	0.11	0.46-1.08	0.71	Chi-square	0.45	0.84-1.49	1.12
rs3781413	Ala418=	Chi-square	0.27	0.46-1.21	0.77	Chi-square	0.17	0.28-1.20	0.62	Chi-square	0.87	0.48-1.73	0.96	Chi-square	0.88	0.62-1.46	0.97
rs3781412	Leu392Pro	Chi-square	0.96	0.74-1.35	1.01	Chi-square	0.32	0.52-1.23	0.81	Chi-square	0.27	0.83-1.93	1.27	Chi-square	0.79	0.71-1.29	0.96
rs372118432	Pro391_Leu392*ins*	Chi-square	0.28	0.47-1.21	0.77	Chi-square	0.18	0.28-1.20	0.62	Chi-square	0.87	0.48-1.73	0.96	Chi-square	0.88	0.62-1.47	0.97
rs535621897	Glu377Asp	NA				NA				NA				NA			
rs45535234	Ser376=	NA				NA				NA				Fisher	0.4488	0.3165-4.6686	1.5575
rs142101185	Ser331=	NA				NA				NA				NA			
rs3781411	Arg298Gln	Chi-square	0.27	0.46-1.21	0.77	Chi-square	0.17	0.27-1.20	0.62	Chi-square	0.87	0.48-1.73	0.96	Chi-square	0.87	0.62-1.46	0.97
rs45440394*	Arg287=	NA				Fisher	0.67	0.15-4.83	1.30	NA				Fisher	0.77	0.08-2.38	0.65
rs3781409	Val234Met	Chi-square	0.37	0.84-1.58	1.16	Chi-square	0.95	0.61-1.54	0.99	Chi-square	0.18	0.86-2.11	1.36	Chi-square	0.87	0.70-1.34	0.97
rs3781408	Asp213Asn	Chi-square	0.54	0.63-2.16	1.23	Fisher	0.23	0.60-3.32	1.54	Fisher	1	0.23-2.32	0.87	Chi-square	0.55	0.62-2.15	1.22
rs116403181	Ser212=	NA				NA				NA				NA			
rs3781407	Asp195=	Fisher	0.13	0.70-3.72	1.76	Fisher	0.47	0.29-4.36	1.44	Fisher	0.13	0.56-5.63	2.11	Fisher	0.07	0.83-3.97	1.96

Only NSVs which were detected in our mutation screen and genotyping approach more than once in at least two study groups were considered. *The NSV was detected only in females. Thus, the statistical analyses were performed only for females. AA: amino acid. p: p-value. 95%CI: 95% confidence interval of odds ratio. OR: odds ratio. NA: not available/applicable.

Supplementary Table 12: Analyzed species and sequences extracted from Ensembl (https://www.ensembl.org/index.html).

Superorder	Species	Version	RIBEYE			CTBP2-L/S		
			Transcript ID	cDNA length	Protein length	Transcript ID	cDNA length	Protein length
Human		GRCh38.p13	ENST00000309035.11	8471	985	ENST00000337195.9	6939	445
Primates	Gorilla	gorGor4	ENSGGOT00000007336.3	2958	985	ENSGGOT00000033882.2	1767	445
	Bonobo	panpan1.1	ENSPPAT00000042272.1	2958	985	ENSPPAT00000042257.1	2642	445
	Chimpanzee	Pan_TRO_3.0	ENSPTRT00000005782.6	2958	985	ENSPTRT00000091768.1	2667	445
	Sumatran orangutan	Susie_PABv2	ENSPPYT00000003315.2	4143	985	ENSPPYT00000003314.3	3133	445
	Pig-tailed macaque	Mnem_1.0	ENSMNET00000065384.1	2964	987	ENSMNET00000065365.1	1918	434
	Drill	Mleu.le_1.0	ENSMLET00000064384.1	2958	985	ENSMLET00000064383.1	2532	445
	Olive baboon	Panubis1.0	ENSPANT00000035943.2	3151	992	ENSPANT00000022708.3	2112	445
	Sooty mangabey	Caty_1.0	ENSCATT00000066980.1	5655	992	ENSCATT00000066975.1	4760	445
	Macaque	Mmul_10	ENSMMUT00000030999.4	2958	985	ENSMMUT00000099570.1	2700	445
	Golden snub-nosed monkey	Rrox_v1	ENSRROT00000060860.1	2958	985	ENSRROT00000060849.1	2895	445
Rodents and related species	Squirrel	SpeTri2.0	ENSSTOT00000036535.1	3248	985	ENSSTOT00000014198.3	2540	445
	Eurasian red squirrel	mSciVul1.1	ENSSVLT00005000453.1	2958	985	ENSSVLT00005000443.1	1338	445
	Alpine marmot	marMar2.1	ENSMMMT00000003026.1	2940	979	ENSMMMT00000003018.1	1338	445
	Mouse	GRCm39	ENSMUST00000169570.8	3604	988	ENSMUST00000033269.15	2321	445
	Northern American deer mouse	HU_Pman_2.1	ENSPEMT00000021243.2	2964	987	ENSPEMT00000021238.2	1338	444
	Ryukyu mouse	CAROLI_EIJ_v1.1	MGP_CAROLIEiJ_T0082832.1	3590	988	MGP_CAROLIEiJ_T0082833.1	1338	445
	Shrew mouse	PAHARI_EIJ_v1.1	MGP_PahariEiJ_T0019620.1	3607	989	MGP_PahariEiJ_T0019622.1	2329	445
	Steppe mouse	MUSP714	ENSMSIT00000035608.1	2967	988	ENSMSIT00000035556.1	2024	445
	Algerian mouse	SPRET_EiJ_v1	MGP_SPRETEiJ_T0086247.1	3605	988	MGP_SPRETEiJ_T0086249.1	1338	445
	Rat	mRatBN7.2	ENSRNOT00000023404.7	3577	988	ENSRNOT00000114061.1	4577	445
Laurasiatheria	Arabian camel	CamDro2	ENSCDRT00005010696.1	3673	983	ENSCDRT00005010640.1	2000	445

	Horse	EquCab3.0	ENSECAT00000024259.3	3138	980	ENSECAT00000060371.1	2222	445
	Blue whale	mBalMus1-v2	ENSBMST00010021268.1	3267	984	ENSBMST00010021217.1	1648	467
	Chacoan peccary	CatWag_v2_BIUU_UCD	ENSCWAT00000024563.1	3326	975	ENSCWAT00000024525.1	2131	445
	Vaquita	mPhoSin1.pri	ENSPSNT00000007959.1	4107	976	ENSPSNT00000007938.1	2804	445
	Pig-Duroc	Sscrofa11.1	ENSSSCT00000011751.3	3647	982	ENSSSCT00000038362.2	2239	445
	Domestic yak	LU_Bosgru_v3.0	ENSBGRT00000014500.1	3119	972	ENSBGRT00000014326.1	1547	448
	Hybrid - Bos Indicus	UOA_Brahman_1	ENSBIXT00005032712.1	4130	971	ENSBIXT00005032740.1	2761	445
	Wild yak	BosGru_v2.0	ENSBMUT00000017452.1	2922	973	ENSBMUT00000017442.1	1948	445
	Cow	ARS-UCD1.2	ENSBTAT00000004405.6	4206	982	ENSBTAT00000004404.6	2518	445
Sauropsida	Indian cobra	Nana_v5	ENSNNAT00000005273.1	3869	992	ENSNNAT00000005268.1	2321	434
	Green anole	AnoCar2.0v2	ENSACAT00000051638.1	3534	1015	ENSACAT00000039130.1	1876	447
Fish	Spotted gar	LepOcu1	ENSLOCT00000009701.1	6003	1027	ENSLOCT00000009685.1	4492	445
	Asian bonytongue	fSclFor1.1	ENSSFOT00015059817.1	3171	1056	ENSSFOT00015015758.2	1299	432
	Northern pike	Eluc_v4	ENSELUT00000082419.1	3216	1071	ENSELUT00000010616.2	1840	457
	Brown trout	fSalTru1.1	ENSSTUT00000030027.1	4972	1089	ENSSTUT00000029977.1	3611	449
	Chinook salmon	Otsh_v1.0	ENSOTST00005026223.1	4188	1079	ENSOTST00005026213.1	1936	442
	Mangrove rivulus	ASM164957v1	ENSKMAT00000018306.1	3745	877	ENSKMAT00000018270.1	2482	445
	Tongue sole	Cse_v1.0	ENSCSET00000024420.1	2151	716	ENSCSET00000024408.1	1380	436
	Lumpfish	fCycLum1.pri	ENSCLMT00005046440.1	2958	696	ENSCLMT00005046429.1	2838	422

Supplementary Table 13: Conservation analyses.

SNP ID	Genomic position (GRCh38)	A1	A2	AA alteration	cDNA					Protein				
					Primate	Laurasiatheria	Sauropsida and Fish	Rodents	Cper.	Primate	Laurasiatheria	Sauropsida and Fish	Rodents	Cper.
rs2946994	chr10: 125026145	G	C	Gln539Glu	10	10	7	10	90.24	10	10	7	10	90.24
rs894087529	chr10: 125026362	C	G	Pro466=	9	9	0	1	46.34	NA				
rs3012075	chr10: 125026397	A	G	Tyr455His	10	10	8	10	92.68	9	9	7	10	85.37
rs3781413	chr10: 125026506	T	C	Ala418=	0	2	1	10	31.71	NA				
rs113477585	chr10: 125026556	G	A	Pro402Ser	10	10	2	7	70.73	9	7	0	0	39.02
rs76134089	chr10: 125026571	G	C	Pro397Ala	10	10	8	10	92.68	10	10	8	10	92.68
rs3781412	chr10: 125026585	A	G	Leu392Pro	10	10	8	10	92.68	10	9	2	10	75.61
rs535621897	chr10: 125026629	T	A	Glu377Asp	10	0	1	3	34.15	10	10	2	10	78.05
rs45535234	chr10: 125026632	G	A	Ser376=	10	9	2	0	51.22	NA				
rs202010294	chr10: 125026676	C	G	Gly362Arg	10	6	2	10	68.29	10	6	1	10	65.85
rs142101185	chr10: 125026767	A	C	Ser331=	10	3	3	9	60.98	NA				
rs769811964	chr10: 125026832	G	C	Leu310Val	10	10	6	10	87.80	10	10	8	10	92.68
rs3781411	chr10: 125026867	C	T	Arg298Gln	10	10	8	9	90.24	10	10	8	9	90.24
rs375685611	chr10: 125026895	C	T	Val289Met	9	10	8	6	80.49	9	9	4	6	68.29
rs45440394	chr10: 125026899	C	T	Arg287=	10	10	6	10	87.80	NA				
rs3781409	chr10: 125027060	C	T	Val234Met	10	9	9	10	92.68	10	9	8	10	90.24
rs3781408	chr10: 125027123	C	T	Asp213Asn	10	5	9	10	82.93	10	5	9	10	82.93
rs116403181	chr10: 125027124	G	A	Ser212=	9	9	4	9	75.61	NA				

rs3781407	chr10: 125027175	G	A	Asp195=	10	8	2	3	56.10	NA				
rs146900874	chr10: 125027546	G	A	Arg72Trp	10	10	9	10	95.12	3	8	0	10	51.22
rs150867595	chr10: 125027603	G	A	Pro53Ser	10	6	7	9	78.05	10	10	7	3	73.17

A1: reference allele. A2: alternative allele. AA: amino acid. Cper.: conservation percentage in %.

Supplementary Table 14: Look-up of detected variants in GWAS for BMI (1).

SNP ID	Genomic position (GRCh38)	A1	A2	AA alteration	Combined sexes					Females					Males				
					EA	NEA	FRQ	Beta	P	EA	NEA	FRQ	Beta	P	EA	NEA	FRQ	Beta	P
**rs2946994**	**chr10: 125026145**	**G**	**C**	**Gln539Glu**	**C**	**G**	**0.49**	**-0.01**	**9.98 E-11**	**C**	**G**	**0.49**	**-0.01**	**1.38 E-08**	C	G	0.49	-0.01	0.001
rs894087529	chr10: 125026362	C	G	Pro466=	NA														
**rs3012075**	**chr10: 125026397**	**A**	**G**	**Tyr455His**	**G**	**A**	**0.49**	**-0.01**	**2.66 E-10**	**G**	**A**	**0.49**	**-0.01**	**2.61 E-08**	G	A	0.49	-0.01	0.002
rs3781413	chr10: 125026506	T	C	Ala418=	C	T	0.13	-0.002	0.35	C	T	0.13	-0.003	0.41	C	T	0.13	-0.001	0.82
rs113477585	chr10: 125026556	G	A	Pro402Ser	A	G	0.001	-0.03	0.35	A	G	0.001	-0.02	0.59	A	G	0.001	-0.01	0.85
rs76134089	chr10: 125026571	G	C	Pro397Ala	C	G	0.002	-0.02	0.28	C	G	0.002	-0.03	0.30	C	G	0.002	-0.01	0.87
rs3781412	chr10: 125026585	A	G	Leu392Pro	G	A	0.39	0.01	7.46 E-08	G	A	0.39	0.01	1.55 E-05	G	A	0.39	0.01	0.003
rs372118432	chr10: 125026591 - 125026597	-	*ins*CT(G)4 CTGCAG GGCCGC AG	Pro391_Leu 392*ins*LeuG lnProGlnPro AlaAla	NA														
rs535621897	chr10: 125026629	T	A	Glu377Asp	A	T	0.001	0.006	0.83	A	T	0.001	0.03	0.48	A	T	0.001	-0.02	0.73
rs45535234	chr10: 125026632	G	A	Ser376=	A	G	0.008	0.02	0.14	A	G	0.01	0.03	0.11	A	G	0.01	0.003	0.87
rs202010294	chr10: 125026676	C	G	Gly362Arg	G	C	0.002	0.05	0.04	G	C	0.002	0.06	0.05	G	C	0.002	0.01	0.87
rs142101185	chr10: 125026767	A	C	Ser331=	C	A	0.002	-0.03	0.26	C	A	0.002	-0.03	0.27	C	A	0.002	-0.01	0.81
rs769811964	chr10: 125026832	G	C	Leu310Val	NA														
rs3781411	chr10: 125026867	C	T	Arg298Gln	T	C	0.13	-0.004	0.09	T	C	0.13	-0.01	0.09	T	C	0.13	-0.002	0.53
rs375685611	chr10: 125026895	C	T	Val289Met	NA														
rs45440394	chr10: 125026899	C	T	Arg287=	T	C	0.02	0.007	0.42	T	C	0.02	0.003	0.79	T	C	0.02	0.01	0.30
**rs3781409**	**chr10: 125027060**	**C**	**T**	**Val234Met**	**T**	**C**	**0.27**	**0.01**	**1.74 E-11**	**T**	**C**	**0.27**	**0.01**	**3.11 E-08**	T	C	0.27	0.01	0.0004
rs3781408	chr10:	C	T	Asp213Asn	T	C	0.05	0.006	0.17	T	C	0.05	0.01	0.16	T	C	0.05	0.004	0.49

	125027123																		
rs116403181	chr10: 125027124	G	A	Ser212=	A	G	0.001	-0.02	0.54	A	G	0.001	0.01	0.85	A	G	0.001	-0.05	0.40
rs3781407	chr10: 125027175	G	A	Asp195=	A	G	0.02	0.007	0.25	A	G	0.02	0.01	0.46	A	G	0.024	0.02	0.06
rs141864737	chr10: 125027235 - 125027236	CT	-	Gln175*fs*	NA														
rs146900874	chr10: 125027546	G	A	Arg72Trp	A	G	0.0001	0.18	0.18	A	G	0.0001	0.14	0.43	A	G	0.0001	0.23	0.28
rs150867595	chr10: 125027603	G	A	Pro53Ser	A	G	0.001	0.03	0.25	A	G	0.001	-0.001	0.97	A	G	0.001	0.06	0.17
rs1379972000	chr10: 125027347-125027367	(G)4A(CCGG)2CTGACT	-	Val132*fs*	NA														

The genome-wide significant variants (for at least one test, so either BMI combined sexes, BMI female, BMI male) are shown in bold (1).
A1: reference allele. A2: alternative allele. AA: amino acid. EA: effect allele. NEA: non-effect allele. FRQ: frequency of effect allele. Beta: effect size. P: P-value. NA: not available/applicable.

Supplementary Table 15: Look-up of detected variants in GWAS for AN (3).

SNP ID	Genomic position (GRCh38)	A1	A2	AA alteration	AN GWAS (3)			
					EA	NEA	Beta	P
rs2946994	chr10: 125026145	G	C	Gln539Glu	C	G	0.013	0.0006
rs894087529	chr10: 125026362	C	G	Pro466=	NA			
rs3012075	chr10: 125026397	A	G	Tyr455His	G	A	0.013	0.0007
rs3781413	chr10: 125026506	T	C	Ala418=	C	T	0.02	0.08
rs113477585	chr10: 125026556	G	A	Pro402Ser	NA			
rs76134089	chr10: 125026571	G	C	Pro397Ala	NA			
rs3781412	chr10: 125026585	A	G	Leu392Pro	G	A	0.014	0.18
rs372118432	chr10: 125026591 - 125026597	-	*ins*CT(G)4CTGCAG GGCCGCAG	Pro391_Leu392*ins*LeuGlnProGln ProAlaAla	NA			
rs535621897	chr10: 125026629	T	A	Glu377Asp	NA			
rs45535234	chr10: 125026632	G	A	Ser376=	NA			
rs202010294	chr10: 125026676	C	G	Gly362Arg	NA			
rs142101185	chr10: 125026767	A	C	Ser331=	NA			
rs769811964	chr10: 125026832	G	C	Leu310Val	NA			
rs3781411	chr10: 125026867	C	T	Arg298Gln	T	C	0.02	0.07
rs375685611	chr10: 125026895	C	T	Val289Met	NA			
rs45440394	chr10: 125026899	C	T	Arg287=	NA			
rs3781409	chr10: 125027060	C	T	Val234Met	T	C	0.02	0.005

rs3781408	chr10: 125027123	C	T	Asp213Asn	T	C	0.03	0.86
rs116403181	chr10: 125027124	G	A	Ser212=	NA			
rs3781407	chr10: 125027175	G	A	Asp195=	A	G	0.05	0.90
rs141864737	chr10: 125027235 – 125027236	CT	-	Gln175*fs*	NA			
rs146900874	chr10: 125027546	G	A	Arg72Trp	NA			
rs150867595	chr10: 125027603	G	A	Pro53Ser	NA			
rs1379972000	chr10: 125027347-125027367	(G)4A(CCGG)2C TGACT	-	Val132*fs*	NA			

A1: reference allele. A2: alternative allele. AA: amino acid. EA: effect allele. NEA: non-effect allele. FRQ: frequency of effect allele. Beta: effect size. P: P-value. NA: not available/applicable.

Supplementary Table 16: Overall predictions of all detected variants in RIBEYE/CTBP2 via MutationTaster2, CADD, PredicSNP2.

SNP ID*	AA alteration	MutationTaster2 (4)			CADD (5)		PredictSNP2 (version: GRCh38.p6) (6)																	
					Version: GRCh38-v1.6		PredictSNP2			CADD			DANN10			FATHMM11			FunSeq2			GWAVA		
		Pred.	PhyloP	Phast Cons	Raw Score	PHRED	Pred.	Score	Exp. acc.	Pred.	Score	Exp. acc.	Pred.	Score	Exp. acc.	Pred.	Score	Exp. acc.	Pred.	Score	Exp. acc.	Pred.	Score	Exp. acc.
rs150867595	Pro53Ser	neut.	2.07	0.99	2.37	22.00	del.	1.00	0.87	del.	19.16	0.52	del.	1.00	0.64	del.	0.93	0.56	del.	4.00	0.62	neut.	0.21	0.54
rs146900874	Arg72Trp	del.	5.33	1.00	3.36	24.30	del.	1.00	0.87	del.	27.10	0.80	del.	1.00	0.77	del.	0.95	0.62	del.	4.00	0.62	neut.	0.19	0.53
rs3781408	Asp213Asn	neut.	1.07	0.01	0.69	8.32	neut.	-1.00	0.89	neut.	10.32	0.86	neut.	0.98	0.67	neut.	0.20	0.84	del.	3.00	0.61	del.	0.25	0.51
rs3781409	Val234Met	neut.	2.98	1.00	2.56	22.60	del.	0.14	0.82	del.	26.10	0.74	del.	1.00	0.75	neut.	0.82	0.63	del.	4.00	0.62	neut.	0.16	0.51
rs375685611	Val289Met	neut.	1.59	0.00	0.43	5.76	neut.	-0.15	0.67	neut.	14.24	0.71	del.	1.00	0.60	neut.	0.30	0.83	del.	3.00	0.61	?14	0.15	0.48
rs3781411	Arg298Gln	neut.	3.09	1.00	3.22	23.90	del.	1.00	0.87	del.	32.00	0.84	del.	1.00	0.77	del.	0.94	0.59	del.	4.00	0.62	neut.	0.14	0.52
rs769811964	Leu310Val	del.	0.24	0.94	1.19	13.53	neut.	-1.00	0.89	neut.	16.60	0.58	neut.	0.98	0.69	neut.	0.87	0.63	del.	3.00	0.61	neut.	0.14	0.52
rs202010294	Gly362Arg	neut.	0.41	0.00	1.36	14.80	neut.	-1.00	0.89	neut.	14.97	0.67	neut.	0.95	0.79	neut.	0.11	0.88	del.	3.00	0.61	-	0.15	0.48
rs535621897	Glu377Asp	neut.	0.03	0.22	0.18	2.86	neut.	-1.00	0.89	neut.	13.64	0.73	neut.	0.31	0.94	neut.	0.10	0.89	del.	3.00	0.61	neut.	0.10	0.52
rs3781412	Leu392Pro	neut.	1.26	1.00	3.02	23.40	neut.	0.00	0.63	del.	19.03	0.52	del.	1.00	0.60	neut.	0.78	0.67	del.	3.00	0.61	neut.	0.16	0.51
rs76134089	Pro397Ala	neut.	2.77	0.98	2.24	21.20	neut.	0.03	0.63	neut.	15.86	0.62	neut.	0.99	0.60	del.	0.90	0.56	del.	4.00	0.62	neut.	0.20	0.53
rs113477585	Pro402Ser	neut.	0.43	0.00	0.18	2.85	neut.	-1.00	0.89	neut.	12.71	0.76	neut.	0.98	0.65	del.	0.18	0.85	del.	4.00	0.62	neut.	0.16	0.51
rs3012075	Tyr455His	neut.	2.97	0.11	1.42	15.24	neut.	-0.08	0.65	del.	20.50	0.52	del.	1.00	0.64	neut.	0.48	0.81	del.	4.00	0.62	neut.	0.14	0.52
rs2946994	Gln539=	neut.	5.65	1.00	3.73	25.50	del.	0.11	0.82	del.	23.30	0.56	neut.	0.99	0.60	del.	0.97	0.73	del.	4.00	0.62	neut.	0.21	0.54
rs3781407	Asp195=	neut.	-0.01	0.00	-0.53	0.10	neut.	-1.00	0.96	neut.	0.02	0.95	neut.	0.42	0.97	neut.	0.03	0.96	neut.	0.00	0.93	neut.	0.25	0.55
rs116403181	Ser212=	neut.	-0.42	0.00	-0.68	0.05	neut.	-1.00	0.96	neut.	0.34	0.95	neut.	0.61	0.96	neut.	0.05	0.94	neut.	0.00	0.93	neut.	0.23	0.59
rs45440394	Arg287=	del.	1.47	0.92	0.07	1.86	neut.	-1.00	0.96	neut.	9.04	0.92	neut.	0.43	0.97	neut.	0.60	0.69	neut.	0.00	0.93	neut.	0.15	0.60
rs142101185	Ser331=	del.	-2.97	0.00	0.06	1.76	neut.	-1.00	0.96	neut.	0.00	0.95	neut.	0.39	0.97	neut.	0.06	0.92	neut.	0.00	0.93	neut.	0.18	0.60
rs45535234	Ser376=	del.	-0.96	0.00	-0.01	1.28	neut.	-1.00	0.96	neut.	6.29	0.93	neut.	0.76	0.90	neut.	0.06	0.92	neut.	0.00	0.93	neut.	0.18	0.60
rs3781413	Ala418=	neut.	-1.46	0.26	-0.58	0.08	neut.	-1.00	0.96	neut.	0.02	0.95	neut.	0.25	0.97	neut.	0.02	0.96	neut.	0.00	0.93	neut.	0.13	0.60
rs894087529	Pro466=	del.	-1.55	0.00	-0.57	0.08	neut.	-1.00	0.96	neut.	7.16	0.91	neut.	0.56	0.97	neut.	0.04	0.96	neut.	0.00	0.93	neut.	0.16	0.60

*Frameshift mutations were not included.

MutationTaster2 (4): http://mutationtaster.org/. PhyloP: A method to determine the grade of conservation of a given nuleotide: A positive PhyloP score (values range between -14 and +6) predicts conservation while sites predicted to be fast-evolving are assigned negative scores - PhastCons: The PhatCons values vary between 0 and 1 and determine the probability of the conservation of nucleotides. The closer the value is to 1, the higher the probability of a conserved position.

CADD (5): https://cadd.gs.washington.edu/snv - RawScore: Raw CADD scores come directly from the modle. The negative scores describe that the variants are likely to be "neutral; positive scores annotate the variants which are likely to be "simulated/not observed""/"deleterious". PHRED: The raw scores are normalized and exported as scaled scores. ~8.6 billion SNPs are scored and ranked. SNPs at the 10%th of CADD scores are assigned to 10; top 1% to 20; 0.1% to 30.
PredictSNP2 (6) (version: GRCh38.p6): https://loschmidt.chemi.muni.cz/predictsnp2/. PredictSNP2: is a consensus classifer for prediction of the effect of nucleotide variants. CADD: Data from CADD is invoked and consolidated by PredictSNP2. DANN: Deleteriouus Annotation of Genetic Variants using Neural Networks (DANN) is a tool for scoring the deleteriousness of single nucleotide variants based on deep neural network lassifier. FATHMM: Functional analysis through hidden markov models (FATHMM) is a tool for scoring the deleteriousness of single nucleotide variants based on SVM classifer. FunSeq2: a tool designed for scoring the deleterousness of SNPs based on weighted scoring system that combines genetic, epigenetic and gene expression information. GWAVA: Genoeme-wide-annotation of variants (GWAVA) is a tool for scoring the deleteriousness of SNPs based on random forest; Exp.acc.: The expect accuracy of prediction tools. The accuracy below 80% will be excluded.
Del.: deleterious. Neut.: neutral. Pred.: prediction.

Supplementary Table 17: Prediction of the position of amino acid deleteriousness via PolyPhen2.0, PROVEAN, SIFT, and HOPE.

SNP ID	AA alteration	PolyPhen2.0 (7)								PROVEAN (8, 9)		SIFT (10)		HOPE (11)						
		HumDiv				HumVar				Cutoff = -2.5		Cutoff = 0.05		Cons.	Mutated AA comparing to wild-type AA				Structure of protein	
		Pred.	score	Sen.	Spec.	Pred.	score	Sen.	Spec.	Pred.	score	Pred.	score	Pred.	size	charge	Hydro-phobicity	Possible effect	Evidence	Pred.
rs150867595	Pro53Ser	Del.	0.717	0.86	0.92	Neut.	0.347	0.85	0.78	Neut.	-1.54	Del.	0.023	Del.	smaller	same	less hydrophobic	loss of interactions;<br>loss of hydrophobic interactions;<br>disturbing the local structure	Proline is rigid and induces a special back bone conformation.	Del.
rs146900874	Arg72Trp	Del.	0.998	0.27	0.99	Del.	0.629	0.8	0.84	Neut.	-1.06	Del.	0.001	Neut.	bigger	wild-type: positive<br><br>mutated: neutral	more hydrophobic	loss of interactions with other molecules or residues;<br>lead to bumps; loss of hydrogen bonds and/or disturb correct folding	NA	NA
rs3781408	Asp213Asn	Neut.	0.078	0.93	0.85	Neut.	0.014	0.95	0.53	Neut.	-1.08	neut.	0.299	Neut.	same	wild-type: negative<br><br>mutated: neutral	same	loss of interactions with other molecules or residues	NA	NA
rs3781409	Val234Met	Neut.	0.208	0.92	0.88	Neut.	0.117	0.9	0.7	Neut.	-0.80	Del.	0.001	Neut.	bigger	same	same	lead to bumps	preferred secondary structures between wild-type and mutated are different	Del.
rs375685611	Val289Met	Neut.	0.28	0.91	0.88	Neut.	0.051	0.93	0.63	Neut.	-0.47	Neut.	0.073	Del.	bigger	same	same	lead to bumps	NA	NA
rs3781411	Arg298Gln	Del.	0.999	0.14	0.99	Del.	0.873	0.71	0.89	Neut.	-0.90	Del.	0.001	Del.	smaller	wild-type: positive<br><br>mutated: neutral	same	loss of interactions with other molecules or residues;<br>loss of interactions	NA	NA
rs769811964	Leu310Val	Del.	0.75	0.85	0.92	Neut.	0.168	0.89	0.72	Neut.	-0.79	Del.	0.000	Neut.	smaller	same	same	loss of interactions	NA	NA
rs202010294	Gly362Arg	Neut.	0.372	0.9	0.89	Neut.	0.238	0.88	0.75	Neut.	-1.13	Del.	0.004	Del.	bigger	wild-type: neutral<br><br>mutated: positive	less hydrophobic	causes repulsion of ligands or other residues;<br>lead to bumps;<br>disturb the local structure	Glycine. the most flexible of all residues. which might be necessary for the protein's function. Glycine can abolish this function	Del.
rs535621897	Glu377Asp	Neut.	0.009	0.96	0.77	Neut.	0.01	0.96	0.5	Neut.	-0.04	Neut.	0.580	Neut.	smaller	same	same	loss of interactions	NA	NA
rs3781412	Leu392Pro	Del.	0.986	0.74	0.96	Del.	0.858	0.72	0.89	Neut.	-1.08	Del.	-1.08	Neut.	smaller	same	same	loss of interactions	NA	NA

rs76134089	Pro397Ala	Del.	0.837	0.84	0.93	Del.	0.475	0.83	0.81	Neut.	-2.09	Del.	0.000	Del.	smaller	same	same	loss of interactions	Proline is rigid and induces a special back bone conformation.	Del.
rs113477585	Pro402Ser	Neut.	0.011	0.96	0.78	Neut.	0.014	0.95	0.53	Neut.	-1.05	Del.	0.014	Del.	smaller	same	less hydrophobic	loss of interactions; loss of hydrophobic interactions	Proline is rigid and induces a special back bone conformation.	Del.
rs3012075	Tyr455His	Neut.	0.001	0.99	0.15	Neut.	0.003	0.98	0.26	Neut.	-0.51	Del.	0.031	Neut.	smaller	same	less hydrophobic	loss of interactions; loss of hydrophobic interactions	NA	NA
rs2946994	Gln539Glu	Del.	0.925	0.81	0.94	Del.	0.513	0.82	0.81	Neut.	-0.37	Neut.	0.086	Neut.	same	wild-type: neutral<br><br>mutated: negative	same	cause repulsion of ligands or other residues	Glutamic acid is located within a stretch of residues annotated as a special region: disordered.	Del.

PolyPhen2.0 [1]: http://genetics.bwh.harvard.edu/pph2/ - To train and test PolyPhen2.0 the datasets HumDiv and HumVar were utilized. The sensitivity value represents the true positive rate which is the chance that the mutaton is classified as damaging when it is indeed damaging. The specificity value: specificity = 1 - false positive rate which is the chance that the benign mutation is correctly classified as benign.
PROVEAN [2,3]: http://provean.jcvi.org/seq_submit.php.
SIFT [4]: Prediction of variants exported from 'Sorting Tolerant From Intolerant' (SIFT)
HOPE [5]: https://www3.cmbi.umcn.nl/hope/. If "smaller": alternative amino acid residue is smaller than the wildtype residue. If "same": charge of alternative and wild-type amino acid residue is unchanged. If "less hydrophobic": alternative amino acid residue is less hydrophobic comparing to the wildtype residue. If "NA": corresponding information/prediction was missing in HOPE.
Cons.: conservation. Del.: deleterious. Neut.: neutral. Pred.: prediction. Sen.: sensitivity. Spec.: specificity.

Supplementary Table 18: The prediction of the impact of NSVs on the alteration of RIBEYE protein stability via MUpro, iStable2.0, and I-Mutant2.0.

SNP ID	AA alteration	MUpro (12)						iStable2.0 (13)				I-Mutant2.0 (14)
		Recommended Predictor		SVM		Neural network		ΔΔG				
		Effect	ΔΔG	effect	Confidence Score	effect	Confidence Score	I-Mutant2.0 SEQ	Mupro_SVM	Mupro_NM	iPTREE-STAB	ΔΔG
rs150867595	Pro53Ser	decrese stability	-0.98	decrese stability	-1.00	decrese stability	-0.98	-1.59	-1.00	-0.98	-4.75	-0.07
rs146900874	Arg72Trp	decrese stability	-0.04	decrese stability	-0.44	decrese stability	-0.76	-0.26	-0.44	-0.76	-1.57	-0.55
rs3781408	Asp213Asn	decrese stability	-1.28	decrese stability	-0.37	decrese stability	-0.80	-1.22	-0.37	-0.80	-2.60	-0.99
rs3781409	Val234Met	decrese stability	-0.86	decrese stability	-1.00	decrese stability	-0.88	-1.02	-1.00	-0.88	-1.38	-0.87
rs375685611	Val289Met	decrese stability	-1.01	decrese stability	-0.83	decrese stability	-0.68	-1.54	-0.83	-0.68	-1.83	-0.07
rs3781411	Arg298Gln	decrese stability	-0.56	decrese stability	-0.35	decrese stability	-0.61	-0.15	-0.35	-0.61	-0.02	-0.24
rs769811964	Leu310Val	decrese stability	-1.04	decrese stability	-0.97	decrese stability	-0.79	-1.20	-0.97	-0.79	0.20	-0.29
rs202010294	Gly362Arg	decrese stability	-0.48	decrese stability	-0.08	decrese stability	-0.67	-0.08	-0.08	-0.67	-1.38	-0.22
rs535621897	Glu377Asp	decrese stability	-1.12	increase stability	0.84	decrese stability	-0.50	-0.37	0.84	-0.50	0.31	-3.58
rs3781412	Leu392Pro	decrese stability	-1.55	decrese stability	-0.72	decrese stability	-0.99	-1.32	-0.72	-0.99	-2.73	0.12
rs76134089	Pro397Ala	decrese stability	-1.69	increase stability	0.08	decrese stability	-0.60	-0.82	0.08	-0.60	-0.89	0.73

rs113477585	Pro402Ser	decrese stability	-0.14	decrese stability	-0.81	increase stability	0.55	-1.52	-0.81	0.55	-5.10	0.53
rs3012075	Tyr455His	decrese stability	-1.30	decrese stability	-0.57	decrese stability	-0.95	-1.19	-0.57	-0.95	-1.17	-0.19
rs2946994	Gln539Glu	decrese stability	-1.20	decrese stability	-0.49	decrese stability	-0.96	0.08	-0.27	-0.84	0.70	0.40

MUpro [12]: http://mupro.proteomics.ics.uci.edu/. Recommended Predictor: Predicted both value and sign of energy change using Support vector machine (SVM) and sequence information only. SVM: Support vector machine. Prediction of sign (direction) of energy change using SVM and sequence information only. Neural network: Prediction of sign (direction) of energy change using Neural Network and sequence information only. decrease stability: The NSV decrease the stability of protein structure. ΔΔG: Free energy change responding to the relative stability change. The sign of ΔΔG predict the directions of mutations directly. Positive ΔΔG: increase stability of protein; Negative ΔΔG: decrease stability of protein. Confidence Score: The range of confidence score is between -1 to 1 to measure the confidence of prediction. A score less than 0 means the mutation decreases the protein stability. The smaller the score, the more confident the prediction is. A score larger than 0, means the mutation increase the protein stability.
iStable2.0 (13): http://ncblab.nchu.edu.tw/iStable2/seqsubmit.html. I-Mutant2.0 SEQ: The prediction is extracted from I-Mutant2.0 adopting an SVM model to approximate the ΔΔG value of the protein and predicts the direcion of stability change utilizing amino acid sequence information. Mupro_SVM & Mupro_NM: The predictions are invoked from Mupro, SVM: Support vector machine, NM: Neural network. iPTREE-STAB: a web-server designed for discriminating the stability of protein (stabilizing or destabilizing) and predicting their stability changes (ΔΔG) upon single amino acid substitutions from amino acid sequence.
I-Mutant2.0 (14): https://folding.biofold.org/i-mutant/i-mutant2.0.html.

Supplementary Table 19: The predictions of SVs via TraP.

SNP ID	AA alteration	TraP (15)	Genoox ACMG Classification		Population frequency	Prediction				Clinical evedence
		Score	prediction	Evidences		Classification	Revel	MetaLR	SpliceAI	ClinVar+UniProt
rs3781407	Asp195=	0.057	Benign	BP7,BA1,BS2	0.0341	BP3	NA	NA	Benign	NA
rs116403181	Ser212=	0.149	Benign	BP7,BA1,BS2,BP6	0.0032	BP3	NA	NA	Benign	Benign
rs45440394	Arg287=	0.065	Benign	BP7,BA1,BS2	0.01	BP3	NA	NA	Benign	NA
rs142101185	Ser331=	0.028	Likely Benign	PM2,BP7,BS2	0.0011	BP3	NA	NA	NA	NA
rs45535234	Ser376=	0.155	Benign	BP7,BS1,BS2	0.0064	BP3	NA	NA	Benign	NA
rs3781413	Ala418=	0.086	Benign	BP7,BA1,BS2	0.133	BP3	NA	NA	NA	NA
rs894087529	Pro466=	0.027	VUS2	PM2,BP7	<0.01%	BP3	NA	NA	NA	NA

TraP (15): http://trap-score.org/. VUS: variants of uncertain significance. NA: not available.

Supplementary Table 20: The possible effects of SVs on the pre-mRNA alternative splicing pattern via ESEfinder3.0, Spliceman and SpliceAI.

SNP ID	AA change	Geno-type	ESEfinder3.0															
			SRSF1			SRSF1 (IgM-BRCA1)			SRSF2			SRSF5			SRSF6			In n/5 ESE
			threshold: 1.956			threshold: 1.867			threshold: 2.383			threshold: 2.67			threshold: 2.676			
			Pos*	Site	Score	Pos*	Site	Score	Pos*	Site	Score	Pos*	Site	Score	Pos*	Site	Score	n
rs3781407	Asp195 =	WT	35 (-17)	CTCCCGT	4.12	35 (-17)	CTCCCGT	4.34	33 (-19)	TGCTCCCG	3.53	15 (-37)	CCATACC	2.43	37 (-15)	CCCGTC	3.00	0
		MUT	35 (-17)	CTCCCGT	4.12	35 (-17)	CTCCCGT	4.34	33 (-19)	TGCTCCCG	3.53	15 (-37)	CCATACC	2.43	37 (-15)	CCCGTC	3.00	
rs116403181	Ser212=	WT	38 (-15)	CTGAGGG	3.89	38 (-15)	CTGAGGG	3.95	21 (-32)	TGTCGCTG	2.93	2 (-51)	TCAATGG	4.51	6 (-47)	TGGGTC	3.07	0
		MUT	38 (-15)	CTGAGGG	3.89	38 (-15)	CTGAGGG	3.95	21 (-32)	TGTCaCTG	3.36	2 (-51)	TCAATGG	4.51	6 (-47)	TGGGTC	3.07	
rs45440394	Arg287=	WT	15 (-37)	CTCAGGG	4.14	15 (-37)	CTCAGGG	4.33	43 (-9)	GGACCCTG	5.44	14 (-38)	ACTCAGG	4.23	24 (-28)	CACGGT	1.32	0
		MUT	15 (-37)	CTCAGGG	4.14	15 (-37)	CTCAGGG	4.33	43 (-9)	GGACCCTG	5.44	14 (-38)	ACTCAGG	4.23	24 (-28)	CACGGT	1.32	
rs142101185	Ser331=	WT	11 (-46)	CCCAGGT	3.91	11 (-46)	CCCAGGT	4.29	43 (-14)	GTCCTCCA	3.95	46 (-11)	CTCCAGG	3.26	40 (-17)	CGCGTC	5.31	0
		MUT	11 (-46)	CCCAGGT	3.91	11 (-46)	CCCAGGT	4.29	43 (-14)	GTCCTCCA	3.95	46 (-11)	CTCCAGG	3.26	40 (-17)	CGCGTC	5.31	
rs45535234	Ser376=	WT	3 (-59)	CAGAGAA	3.16	3 (-59)	CAGAGAA	3.03	54 (-8)	GGACCGCG	3.88	49 (-13)	CTGCTGG	2.80	32 (-30)	CGCTTC	3.00	2
		MUT	3 (-59)	CAGAGAA	3.16	3 (-59)	CAGAGAA	3.03	54 (-8)	GGACCGCG	3.88	31 (-31)	TCaCTTC	3.21	44 (-18)	TGAAGC	2.57	
rs3781413	Ala418=	WT	27 (-37)	CTGCCGT	3.87	27 (-37)	CTGCCGT	3.95	25 (-39)	GGCTGCCG	4.68	34 (-30)	CTCCAGG	3.26	44 (-20)	TGCTGA	2.65	1
		MUT	27 (-37)	CcGCCGT	3.27	57 (-7)	CGCTGGA	3.83	25 (-39)	GGCcGCCG	4.91	34 (-30)	CTCCAGG	3.26	44 (-20)	TGCTGA	2.65	
rs894087529	Pro466=	WT	29 (-33)	GGCCCGG	2.84	48 (-14)	CACCCCT	3.12	48 (-14)	CACCCCTG	4.06	22 (-40)	TTTGAGG	3.24	2 (-60)	TGGGGC	2.26	1
		MUT	48 (-14)	CACCCCT	2,66	48 (-14)	CACCCCT	3,12	48 (-14)	CACCCCTG	4,06	22 (-40)	TTTGAGG	3,24	2 (-60)	TGGGGC	2,26	

ESEfinder3.0 (16, 17): http://rulai.cshl.edu/cgi-bin/tools/ESE3/esefinder.cgi?Process=home. SRSF1: SF2/ASF round winner. SRSFrom(IgM-BRCA1): Smith06-HMG-matrix). SRSF2: SC35 round 3 winner. SRSF5: SRp40 round 3 winner. SRSF6: SRp55 round 3 winner. In n/5 putative ESE: n/5 refers to the number of ESE element which was influenced by variant.

Spliceman (18):http://fairbrother.biomed.brown.edu/spliceman/. L1 distance: Manhattan distance. Ranking(L1): the ranking-percentile of the tested variants based on L1 distance. If the ranking of a variant that was located in the exon, intron, and 3’ region, is larger than 0.57, 0.66, and 0.71, respectively, it is predicted as pathogenic [3].

SpliceAI (19): https://spliceailookup.broadinstitute.org/. Δscore: The range is from 0~1. It can be interpreted as the probability that the variant affects splicing at any position within a window around it. 0.2<Δscore<0.5 high recall; 0.5<Δscore<0.8 recommended; Δscore>0.8 high precision. pre-mRNA position: the possible different positions in the pre-mRNA being splice acceptors or donors affecting by the variants.

Supplementary Table 21:LD analysis for detected variants and three Hinney et al. 2017 AN and BMI relevant SNPs (20).

R^2																					
RS_number	rs11245456	rs12771627	rs1561589	rs2946994	rs3012075	rs3781413	rs113477585	rs76134089	rs3781412	rs535621897	rs45535234	rs202010294	rs142101185	rs3781411	rs45440394	rs3781409	rs3781408	rs116403181	rs3781407	rs141864737	rs150867595
rs11245456	1	0.564	0.397	0.173	0.178	0.045	NA	NA	0.111	NA	0.001	NA	NA	0.045	0	0.244	0.017	NA	0.01	0.004	NA
rs12771627	0.564	1	0.631	0.335	0.342	0.043	NA	NA	0.18	NA	0.01	NA	NA	0.043	0.008	0.345	0.016	NA	0.01	0.004	NA
rs1561589	0.397	0.631	1	0.543	0.554	0.069	NA	NA	0.412	NA	0.042	NA	NA	0.069	0.013	0.73	0.026	NA	0.016	0.006	NA
rs2946994	0.173	0.335	0.543	1	0.98	0.106	NA	NA	0.652	NA	0.024	NA	NA	0.106	0.022	0.419	0.045	NA	0.024	0.011	NA
rs3012075	0.178	0.342	0.554	0.98	1	0.108	NA	NA	0.665	NA	0.025	NA	NA	0.108	0.021	0.428	0.044	NA	0.025	0.011	NA
rs3781413	0.045	0.043	0.069	0.106	0.108	1	NA	NA	0.162	NA	0.003	NA	NA	1	0.002	0.05	0.005	NA	0.231	0.001	NA
rs113477585	NA	NA	NA	NA	NA	NA	NA	NA	NA	NA	NA	NA	NA	NA	NA	NA	NA	NA	NA	NA	NA
rs76134089	NA	NA	NA	NA	NA	NA	NA	NA	NA	NA	NA	NA	NA	NA	NA	NA	NA	NA	NA	NA	NA
rs3781412	0.111	0.18	0.412	0.652	0.665	0.162	NA	NA	1	NA	0.037	NA	NA	0.162	0.014	0.643	0.029	NA	0.037	0.007	NA
rs535621897	NA	NA	NA	NA	NA	NA	NA	NA	NA	NA	NA	NA	NA	NA	NA	NA	NA	NA	NA	NA	NA
rs45535234	0.001	0.01	0.042	0.024	0.025	0.003	NA	NA	0.037	NA	1	NA	NA	0.003	0.001	0.058	0.001	NA	0.001	0	NA
rs202010294	NA	NA	NA	NA	NA	NA	NA	NA	NA	NA	NA	NA	NA	NA	NA	NA	NA	NA	NA	NA	NA
rs142101185	NA	NA	NA	NA	NA	NA	NA	NA	NA	NA	NA	NA	NA	NA	NA	NA	NA	NA	NA	NA	NA
rs3781411	0.045	0.043	0.069	0.106	0.108	1	NA	NA	0.162	NA	0.003	NA	NA	1	0.002	0.05	0.005	NA	0.231	0.001	NA
rs45440394	0	0.008	0.013	0.022	0.021	0.002	NA	NA	0.014	NA	0.001	NA	NA	0.002	1	0.009	0.001	NA	0.001	0	NA
rs3781409	0.244	0.345	0.73	0.419	0.428	0.05	NA	NA	0.643	NA	0.058	NA	NA	0.05	0.009	1	0.019	NA	0.012	0.005	NA
rs3781408	0.017	0.016	0.026	0.045	0.044	0.005	NA	NA	0.029	NA	0.001	NA	NA	0.005	0.001	0.019	1	NA	0.001	0	NA
rs116403181	NA	NA	NA	NA	NA	NA	NA	NA	NA	NA	NA	NA	NA	NA	NA	NA	NA	NA	NA	NA	NA
rs3781407	0.01	0.01	0.016	0.024	0.025	0.231	NA	NA	0.037	NA	0.001	NA	NA	0.231	0.001	0.012	0.001	NA	1	0	NA
rs141864737	0.004	0.004	0.006	0.011	0.011	0.001	NA	NA	0.007	NA	0	NA	NA	0.001	0	0.005	0	NA	0	1	NA

rs150867595	NA	NA	NA	NA	NA	NA	NA	NA	NA	NA	NA	NA	NA	NA	NA	NA	NA	NA	NA	NA	NA

D'

RS_number	rs11245456	rs12771627	rs1561589	rs2946994	rs3012075	rs3781413	rs113477585	rs76134089	rs3781412	rs535621897	rs45535234	rs202010294	rs142101185	rs3781411	rs45440394	rs3781409	rs3781408	rs116403181	rs3781407	rs141864737	rs150867595
rs11245456	1	0.77	0.774	0.674	0.678	1	NA	NA	0.436	NA	0.305	NA	NA	1	0.132	0.518	1	NA	1	1	NA
rs12771627	0.77	1	1	0.963	0.963	1	NA	NA	0.569	NA	1	NA	NA	1	1	0.632	1	NA	1	1	NA
rs1561589	0.774	1	1	0.973	0.973	1	NA	NA	0.684	NA	1	NA	NA	1	1	1	1	NA	1	1	NA
rs2946994	0.674	0.963	0.973	1	1	1	NA	NA	1	NA	1	NA	NA	1	1	1	1	NA	1	1	NA
rs3012075	0.678	0.963	0.973	1	1	1	NA	NA	1	NA	1	NA	NA	1	1	1	1	NA	1	1	NA
rs3781413	1	1	1	1	1	1	NA	NA	1	NA	1	NA	NA	1	1	1	1	NA	1	1	NA
rs113477585	NA	NA	NA	NA	NA	NA	NA	NA	NA	NA	NA	NA	NA	NA	NA	NA	NA	NA	NA	NA	NA
rs76134089	NA	NA	NA	NA	NA	NA	NA	NA	NA	NA	NA	NA	NA	NA	NA	NA	NA	NA	NA	NA	NA
rs3781412	0.436	0.569	0.684	1	1	1	NA	NA	1	NA	1	NA	NA	1	1	1	1	NA	1	1	NA
rs535621897	NA	NA	NA	NA	NA	NA	NA	NA	NA	NA	NA	NA	NA	NA	NA	NA	NA	NA	NA	NA	NA
rs45535234	0.305	1	1	1	1	1	NA	NA	1	NA	1	NA	NA	1	1	1	1	NA	1	1	NA
rs202010294	NA	NA	NA	NA	NA	NA	NA	NA	NA	NA	NA	NA	NA	NA	NA	NA	NA	NA	NA	NA	NA
rs142101185	NA	NA	NA	NA	NA	NA	NA	NA	NA	NA	NA	NA	NA	NA	NA	NA	NA	NA	NA	NA	NA
rs3781411	1	1	1	1	1	1	NA	NA	1	NA	1	NA	NA	1	1	1	1	NA	1	1	NA
rs45440394	0.132	1	1	1	1	1	NA	NA	1	NA	1	NA	NA	1	1	1	1	NA	1	1	NA
rs3781409	0.518	0.632	1	1	1	1	NA	NA	1	NA	1	NA	NA	1	1	1	1	NA	1	1	NA
rs3781408	1	1	1	1	1	1	NA	NA	1	NA	1	NA	NA	1	1	1	1	NA	1	1	NA
rs116403181	NA	NA	NA	NA	NA	NA	NA	NA	NA	NA	NA	NA	NA	NA	NA	NA	NA	NA	NA	NA	NA
rs3781407	1	1	1	1	1	1	NA	NA	1	NA	1	NA	NA	1	1	1	1	NA	1	1	NA
rs141864737	1	1	1	1	1	1	NA	NA	1	NA	1	NA	NA	1	1	1	1	NA	1	1	NA
rs150867595	NA	NA	NA	NA	NA	NA	NA	NA	NA	NA	NA	NA	NA	NA	NA	NA	NA	NA	NA	NA	NA

The SNPs identified to be associated with AN and BMI in (20) are shown in blue. The SNPs in yellow were identified in the here performed analyses.
NA: not available.

Supplementary Table 22: R^2 values of LD analysis for detected variants and genome-wide significant Pulit et al. BMI relevant SNPs (1).

R^2	SNP ID	rs2946994	rs3012075	rs3781413	rs113477585	rs76134089	rs3781412	rs535621897	rs45535234	rs202010294	rs142101185	rs3781411	rs45440394	rs3781409	rs3781408	rs116403181	rs3781407	rs141864737	rs150867595
	rs11245405	0.03	0.028	0.003			0.007		0.008			0.003	0	0.016	0.003		0	0.003	
	rs11245406	0.03	0.028	0.003			0.007		0.008			0.003	0	0.016	0.003		0	0.003	
	rs11245407	0.03	0.028	0.003			0.007		0.008			0.003	0	0.016	0.003		0	0.003	
	rs10901833	0.03	0.028	0.003			0.007		0.008			0.003	0	0.016	0.003		0	0.003	
	rs12355063	0.03	0.028	0.003			0.007		0.008			0.003	0	0.016	0.003		0	0.003	
	rs12247671	0.026	0.025	0.004			0.005		0.007			0.004	0	0.014	0.002		0	0.003	
	rs7923382	0.026	0.025	0.004			0.005		0.007			0.004	0	0.014	0.002		0	0.003	
	rs35186116	0.162	0.167	0.032			0.104		0.001			0.032	0	0.211	0.017		0.011	0.004	
	rs72828935	0.141	0.146	0.043			0.096		0.001			0.043	0	0.217	0.016		0.01	0.004	
	rs17636031	0.155	0.16	0.045			0.096		0.001			0.045	0	0.221	0.017		0.01	0.004	
	rs75274506	0.043	0.042	0.002			0.021		0.002			0.002	0.002	0.016	0.004		0.004	0.001	
	rs12220302	0.212	0.202	0.011			0.084		0.032			0.011	0.016	0.057	0.001		0.013	0.008	
	rs75011412	0.038	0.037	0.001			0.018		0.002			0.001	0.002	0.014	0.004		0.005	0.001	
	rs4962708	0.07	0.072	0.024			0.016		0			0.024	0.004	0.056	0.009		0.006	0.002	
	rs4962709	0.075	0.078	0.025			0.013		0			0.025	0.005	0.05	0.009		0.006	0.002	
	rs67609008	0.155	0.16	0.045			0.096		0.001			0.045	0	0.221	0.017		0.01	0.004	
	rs11245446	0.176	0.182	0.027			0.086		0.002			0.027	0.001	0.175	0.021		0.013	0.005	
	rs11245450	0.421	0.41	0			0.223		0.019			0	0.009	0.24	0.056		0.015	0.014	
	rs78159028	0.033	0.032	0.001			0.015		0.002			0.001	0.002	0.012	0.003		0.006	0.001	

**rs76497870**	0.043	0.042	0.002			0.021		0.002			0.002	0.002	0.016	0.004		0.004	0.001	
**rs11245453**	0.188	0.193	0.048			0.127		0.001			0.048	0	0.272	0.018		0.011	0.004	
**rs11245454**	0.188	0.193	0.048			0.127		0.001			0.048	0	0.272	0.018		0.011	0.004	
**rs11245455**	0.225	0.231	0.044			0.152		0.001			0.044	0	0.305	0.017		0.01	0.004	
**rs11245456**	0.173	0.178	0.045			0.111		0.001			0.045	0	0.244	0.017		0.01	0.004	
**rs3781446**	0.474	0.462	0.016			0.326		0.02			0.016	0.008	0.275	0.001		0	0.013	
**rs11245458**	0.192	0.198	0.045			0.127		0.01			0.045	0.008	0.268	0.017		0.01	0.004	
**rs12414708**	0.637	0.622	0.032			0.373		0.022			0.032	0.024	0.285	0.049		0.007	0.012	
**rs12782469**	0.319	0.326	0.03			0.17		0.001			0.03	0.008	0.305	0.017		0.001	0.004	
**rs3781445**	0.536	0.524	0.033			0.315		0.019			0.033	0.029	0.229	0.058		0.019	0.007	
**rs3781444**	0.733	0.749	0.073			0.635		0.032			0.073	0.017	0.452	0.034		0.032	0.008	
**rs11598549**	0.326	0.333	0.028			0.211		0.001			0.028	0.008	0.358	0.016		0.001	0.004	
**rs7923776**	0.761	0.778	0.065			0.676		0.029			0.065	0.018	0.503	0.037		0.029	0.009	
**rs3781442**	0.761	0.778	0.065			0.676		0.029			0.065	0.018	0.503	0.037		0.029	0.009	
**rs12771627**	0.335	0.342	0.043			0.18		0.01			0.043	0.008	0.345	0.016		0.01	0.004	
**rs1561589**	0.543	0.554	0.069			0.412		0.042			0.069	0.013	0.73	0.026		0.016	0.006	
**rs3781433**	0.781	0.797	0.067			0.691		0.03			0.067	0.018	0.513	0.037		0.03	0.009	
**rs1970811**	0.703	0.718	0.012			0.599		0.033			0.012	0.016	0.568	0.033		0.02	0.008	
**rs2028398**	0.543	0.554	0.069			0.412		0.042			0.069	0.013	0.73	0.026		0.016	0.006	
**rs2028397**	0.543	0.554	0.069			0.412		0.042			0.069	0.013	0.73	0.026		0.016	0.006	
**rs4962719**	0.543	0.554	0.069			0.412		0.042			0.069	0.013	0.73	0.026		0.016	0.006	

**rs4962720**	0.344	0.352	0.044			0.19		0.01			0.044	0.008	0.361	0.017		0.01	0.004	
**rs4962416**	0.344	0.352	0.044			0.19		0.01			0.044	0.008	0.361	0.017		0.01	0.004	
**rs4962419**	0.344	0.352	0.044			0.19		0.01			0.044	0.008	0.361	0.017		0.01	0.004	
**rs12769019**	0.344	0.352	0.044			0.19		0.01			0.044	0.008	0.361	0.017		0.01	0.004	
**rs12769682**	0.344	0.352	0.044			0.19		0.01			0.044	0.008	0.361	0.017		0.01	0.004	
**rs2018366**	0.419	0.428	0.05			0.643		0.058			0.05	0.009	1	0.019		0.012	0.005	
**rs11245462**	0.391	0.399	0.05			0.608		0.058			0.05	0.009	0.953	0.019		0.012	0.005	
**rs3781428**	0.419	0.428	0.05			0.643		0.058			0.05	0.009	1	0.019		0.012	0.005	
**rs3781426**	0.419	0.428	0.05			0.643		0.058			0.05	0.009	1	0.019		0.012	0.005	
**rs60306160**	0.401	0.409	0.051			0.623		0.057			0.051	0	0.977	0.019		0.012	0.005	
**rs3781424**	0.419	0.428	0.05			0.643		0.058			0.05	0.009	1	0.019		0.012	0.005	
**rs4962723**	0.419	0.428	0.05			0.643		0.058			0.05	0.009	1	0.019		0.012	0.005	
**rs3781422**	0.781	0.797	0.067			0.691		0.03			0.067	0.018	0.513	0.037		0.03	0.009	
**rs3781421**	0.419	0.428	0.05			0.643		0.058			0.05	0.009	1	0.019		0.012	0.005	
**rs4962420**	0.419	0.428	0.05			0.643		0.058			0.05	0.009	1	0.019		0.012	0.005	
**rs4109292**	0.94	0.96	0.087			0.632		0.025			0.087	0.021	0.428	0.044		0.025	0.011	
**rs12357688**	0.409	0.418	0.049			0.628		0.06			0.049	0.009	0.976	0.018		0.011	0.004	
**rs7073257**	0.419	0.428	0.05			0.643		0.058			0.05	0.009	1	0.019		0.012	0.005	
**rs7086797**	0.471	0.48	0.004			0.722		0.052			0.004	0.01	0.891	0.021		0.052	0.005	
**rs3781416**	0.419	0.428	0.05			0.643		0.058			0.05	0.009	1	0.019		0.012	0.005	
**rs3781415**	0.96	0.98	0.106			0.652		0.024			0.106	0.022	0.419	0.045		0.024	0.011	

**rs4411245**	0.481	0.491	0.001			0.739		0.051			0.001	0.011	0.871	0.022		0.051	0.005	
**rs11245469**	0.419	0.428	0.05			0.643		0.058			0.05	0.009	1	0.019		0.012	0.005	
**rs10444192**	0.419	0.428	0.05			0.643		0.058			0.05	0.009	1	0.019		0.012	0.005	
**rs2363893**	0.98	1	0.108			0.665		0.025			0.108	0.021	0.428	0.044		0.025	0.011	
**rs2938005**	0.921	0.941	0.102			0.626		0.023			0.102	0.023	0.402	0.013		0.023	0.011	
**rs3012067**	0.921	0.941	0.102			0.626		0.023			0.102	0.023	0.402	0.013		0.023	0.011	
**rs558740521**																		
**rs2938004**	0.921	0.941	0.102			0.626		0.023			0.102	0.023	0.402	0.013		0.023	0.011	
**rs11245472**	0.419	0.428	0.05			0.643		0.058			0.05	0.009	1	0.019		0.012	0.005	
**rs2938002**	0.921	0.941	0.102			0.626		0.023			0.102	0.023	0.402	0.013		0.023	0.011	
**rs2919286**	0.921	0.941	0.102			0.626		0.023			0.102	0.023	0.402	0.013		0.023	0.011	
**rs2949369**	0.921	0.941	0.102			0.626		0.023			0.102	0.023	0.402	0.013		0.023	0.011	
**rs2913113**	0.921	0.941	0.102			0.626		0.023			0.102	0.023	0.402	0.013		0.023	0.011	
**rs2938001**	0.921	0.941	0.102			0.626		0.023			0.102	0.023	0.402	0.013		0.023	0.011	
**rs2913112**	0.921	0.941	0.102			0.626		0.023			0.102	0.023	0.402	0.013		0.023	0.011	
**rs893857**	0.409	0.418	0.049			0.628		0.06			0.049	0.009	0.976	0.018		0.011	0.004	
**rs2839737**	0.921	0.941	0.102			0.626		0.023			0.102	0.023	0.402	0.013		0.023	0.011	
**rs1561586**	0.902	0.921	0.104			0.606		0.024			0.104	0.022	0.383	0.012		0.024	0.011	
**rs2043953**	0.921	0.941	0.102			0.626		0.023			0.102	0.023	0.402	0.013		0.023	0.011	
**rs2913111**	0.902	0.921	0.104			0.606		0.024			0.104	0.022	0.383	0.012		0.024	0.011	
**rs3012074**	0.921	0.941	0.102			0.626		0.023			0.102	0.023	0.402	0.013		0.023	0.011	

**rs10901851**	0.409	0.418	0.049			0.628		0.06			0.049	0.009	0.976	0.018		0.011	0.004	
**rs11245478**	0.391	0.399	0.05			0.608		0.058			0.05	0.009	0.953	0.019		0.012	0.005	
**rs4962421**	0.391	0.399	0.05			0.608		0.058			0.05	0.009	0.953	0.019		0.012	0.005	
**rs3781396**	0.428	0.437	0.001			0.68		0.047			0.001	0.011	0.814	0.002		0.047	0.006	
**rs2949368**	0.902	0.922	0.1			0.613		0.023			0.1	0.023	0.394	0.013		0.023	0.012	
**rs2937999**	0.902	0.922	0.1			0.613		0.023			0.1	0.023	0.394	0.013		0.023	0.012	
**rs2028395**	0.633	0.647	0.138			0.848		0.032			0.138	0.017	0.545	0.007		0.032	0.008	
**rs3781395**	0.391	0.399	0.05			0.608		0.058			0.05	0.009	0.953	0.019		0.012	0.005	
**rs2289431**	0.428	0.437	0.001			0.68		0.047			0.001	0.011	0.814	0.002		0.047	0.006	
**rs11245479**	0.391	0.399	0.05			0.608		0.058			0.05	0.009	0.953	0.019		0.012	0.005	
**rs11245480**	0.428	0.437	0.001			0.68		0.047			0.001	0.011	0.814	0.002		0.047	0.006	
**rs2946993**	0.902	0.922	0.1			0.613		0.023			0.1	0.023	0.394	0.013		0.023	0.012	
**rs10901852**	0.382	0.39	0.049			0.593		0.06			0.049	0.009	0.93	0.018		0.011	0.004	
**rs4962422**	0.391	0.399	0.05			0.608		0.058			0.05	0.009	0.953	0.019		0.012	0.005	
**rs4962424**	0.428	0.437	0.001			0.68		0.047			0.001	0.011	0.814	0.002		0.047	0.006	
**rs2935653**	0.883	0.902	0.102			0.626		0.023			0.102	0.023	0.402	0.013		0.023	0.011	
**rs2936546**	0.865	0.886	0.096			0.589		0.022			0.096	0.024	0.379	0.014		0.022	0.012	
**rs11245481**	0.391	0.399	0.05			0.608		0.058			0.05	0.009	0.953	0.019		0.012	0.005	
**rs4962724**	0.384	0.392	0.052			0.604		0.056			0.052	0.01	0.954	0.02		0.012	0.005	
**rs4962725**	0.57	0.583	0.01			0.399		0.032			0.01	0.017	0.545	0.007		0.032	0.008	
**rs10901854**	0.374	0.382	0.051			0.589		0.057			0.051	0.009	0.931	0.019		0.012	0.005	

	**rs2949371**	0.848	0.868	0.094			0.577		0.022			0.094	0.025	0.371	0.015		0.022	0.012	
	**rs2949372**	0.848	0.868	0.094			0.577		0.022			0.094	0.025	0.371	0.015		0.022	0.012	
	**rs3012065**	0.442	0.451	0.056			0.171		0.013			0.056	0.01	0.355	0.021		0.013	0.005	
	**rs3012066**	0.442	0.451	0.056			0.171		0.013			0.056	0.01	0.355	0.021		0.013	0.005	
	**rs2946996**	0.472	0.483	0.005			0.226		0.017			0.005	0.014	0.303	0.004		0.039	0.007	
	**rs2919290**	0.674	0.69	0.124			0.395		0.023			0.124	0.019	0.193	0.009		0.029	0.009	
	**rs718947**	0.125	0.131	0.002			0.23		0.003			0.002	0.015	0.293	0.005		0.036	0.014	
	**rs718948**	0.148	0.155	0			0.26		0.004			0	0.015	0.302	0.015		0.037	0.014	

SNPs with a genome-wide significance for the BMI which are located in *CTBP2* and adjacent regions (± 500 kb) (1) were included in the LD analyses. Here, just the LD values (R^2) for the genome-wide significant SNPs with the here detected variants are shown. No LD structures between the genome-wide SNP are presented.

Supplementary Table 23: D’ values of LD analysis for detected variants and genome-wide significant Pulit et al. BMI relevant SNPs (1).

D'	SNP ID	rs2946994	rs3012075	rs3781413	rs113477585	rs76134089	rs3781412	rs535621897	rs45535234	rs202010294	rs142101185	rs3781411	rs45440394	rs3781409	rs3781408	rs116403181	rs3781407	rs141864737	rs150867595
	**rs11245405**	0.31	0.303	0.094			0.185		1			0.094	0.029	0.351	0.45		0.12	1	
	**rs11245406**	0.31	0.303	0.094			0.185		1			0.094	0.029	0.351	0.45		0.12	1	
	**rs11245407**	0.31	0.303	0.094			0.185		1			0.094	0.029	0.351	0.45		0.12	1	
	**rs10901833**	0.31	0.303	0.094			0.185		1			0.094	0.029	0.351	0.45		0.12	1	
	**rs12355063**	0.31	0.303	0.094			0.185		1			0.094	0.029	0.351	0.45		0.12	1	
	**rs12247671**	0.294	0.287	0.1			0.167		1			0.1	0.036	0.336	0.438		0.1	1	
	**rs7923382**	0.294	0.287	0.1			0.167		1			0.1	0.036	0.336	0.438		0.1	1	
	**rs35186116**	0.644	0.648	0.829			0.416		0.317			0.829	0.147	0.477	1		1	1	
	**rs72828935**	0.625	0.629	1			0.415		0.28			1	0.1	0.501	1		1	1	
	**rs17636031**	0.638	0.642	1			0.406		0.305			1	0.132	0.493	1		1	1	
	**rs75274506**	0.657	0.654	0.418			0.569		1			0.418	1	0.618	1		0.125	1	
	**rs12220302**	0.531	0.513	0.28			0.311		1			0.28	1	0.321	0.1		0.64	1	
	**rs75011412**	0.636	0.632	0.381			0.542		1			0.381	1	0.594	1		0.13	1	
	**rs4962708**	0.588	0.592	1			0.226		0.028			1	1	0.339	1		1	1	
	**rs4962709**	0.599	0.603	1			0.201		0.022			1	1	0.318	1		1	1	
	**rs67609008**	0.638	0.642	1			0.406		0.305			1	0.132	0.493	1		1	1	
	**rs11245446**	0.619	0.623	0.695			0.349		0.391			0.695	0.238	0.439	1		1	1	
	**rs11245450**	0.726	0.723	0.068			0.655		1			0.068	0.562	0.847	1		0.65	1	
	**rs78159028**	0.612	0.608	0.34			0.511		1			0.34	1	0.567	1		0.134	1	

**rs76497870**	0.657	0.654	0.418			0.569		1			0.418	1	0.618	1		0.125	1	
**rs11245453**	0.685	0.689	1			0.455		0.329			1	0.161	0.535	1		1	1	
**rs11245454**	0.685	0.689	1			0.455		0.329			1	0.161	0.535	1		1	1	
**rs11245455**	0.779	0.781	1			0.516		0.293			1	0.116	0.587	1		1	1	
**rs11245456**	0.674	0.678	1			0.436		0.305			1	0.132	0.518	1		1	1	
**rs3781446**	0.755	0.752	0.431			0.775		1			0.431	0.554	0.888	0.108		0.09	1	
**rs11245458**	0.711	0.714	1			0.466		1			1	1	0.544	1		1	1	
**rs12414708**	0.831	0.83	0.57			0.787		1			0.57	1	0.859	1		0.57	1	
**rs12782469**	0.926	0.927	0.823			0.547		0.293			0.823	1	0.587	1		0.293	1	
**rs3781445**	0.836	0.835	0.642			0.794		1			0.642	1	0.844	1		1	1	
**rs3781444**	0.977	0.977	0.728			0.865		1			0.728	1	0.911	1		1	1	
**rs11598549**	0.962	0.962	0.817			0.624		0.267			0.817	1	0.652	1		0.267	1	
**rs7923776**	0.956	0.956	0.717			0.93		1			0.717	1	1	1		1	1	
**rs3781442**	0.956	0.956	0.717			0.93		1			0.717	1	1	1		1	1	
**rs12771627**	0.963	0.963	1			0.569		1			1	1	0.632	1		1	1	
**rs1561589**	0.973	0.973	1			0.684		1			1	1	1	1		1	1	
**rs3781433**	0.978	0.978	0.72			0.931		1			0.72	1	1	1		1	1	
**rs1970811**	0.976	0.977	0.286			0.824		1			0.286	1	1	1		1	1	
**rs2028398**	0.973	0.973	1			0.684		1			1	1	1	1		1	1	
**rs2028397**	0.973	0.973	1			0.684		1			1	1	1	1		1	1	
**rs4962719**	0.973	0.973	1			0.684		1			1	1	1	1		1	1	

**rs4962720**	0.963	0.964	1			0.577		1			1	1	0.639	1		1	1	
**rs4962416**	0.963	0.964	1			0.577		1			1	1	0.639	1		1	1	
**rs4962419**	0.963	0.964	1			0.577		1			1	1	0.639	1		1	1	
**rs12769019**	0.963	0.964	1			0.577		1			1	1	0.639	1		1	1	
**rs12769682**	0.963	0.964	1			0.577		1			1	1	0.639	1		1	1	
**rs2018366**	1	1	1			1		1			1	1	1	1		1	1	
**rs11245462**	0.966	0.967	1			0.972		1			1	1	0.976	1		1	1	
**rs3781428**	1	1	1			1		1			1	1	1	1		1	1	
**rs3781426**	1	1	1			1		1			1	1	1	1		1	1	
**rs60306160**	0.967	0.967	1			0.973		1			1	0.202	1	1		1	1	
**rs3781424**	1	1	1			1		1			1	1	1	1		1	1	
**rs4962723**	1	1	1			1		1			1	1	1	1		1	1	
**rs3781422**	0.978	0.978	0.72			0.931		1			0.72	1	1	1		1	1	
**rs3781421**	1	1	1			1		1			1	1	1	1		1	1	
**rs4962420**	1	1	1			1		1			1	1	1	1		1	1	
**rs4109292**	0.98	0.98	0.898			0.975		1			0.898	1	1	1		1	1	
**rs12357688**	1	1	1			1		1			1	1	1	1		1	1	
**rs7073257**	1	1	1			1		1			1	1	1	1		1	1	
**rs7086797**	1	1	0.25			1		1			0.25	1	1	1		1	1	
**rs3781416**	1	1	1			1		1			1	1	1	1		1	1	
**rs3781415**	0.98	1	1			1		1			1	1	1	1		1	1	

**rs4411245**	1	1	0.113			1		1			0.113	1	1	1		1	1	
**rs11245469**	1	1	1			1		1			1	1	1	1		1	1	
**rs10444192**	1	1	1			1		1			1	1	1	1		1	1	
**rs2363893**	1	1	1			1		1			1	1	1	1		1	1	
**rs2938005**	0.979	1	1			1		1			1	1	1	0.524		1	1	
**rs3012067**	0.979	1	1			1		1			1	1	1	0.524		1	1	
**rs558740521**																		
**rs2938004**	0.979	1	1			1		1			1	1	1	0.524		1	1	
**rs11245472**	1	1	1			1		1			1	1	1	1		1	1	
**rs2938002**	0.979	1	1			1		1			1	1	1	0.524		1	1	
**rs2919286**	0.979	1	1			1		1			1	1	1	0.524		1	1	
**rs2949369**	0.979	1	1			1		1			1	1	1	0.524		1	1	
**rs2913113**	0.979	1	1			1		1			1	1	1	0.524		1	1	
**rs2938001**	0.979	1	1			1		1			1	1	1	0.524		1	1	
**rs2913112**	0.979	1	1			1		1			1	1	1	0.524		1	1	
**rs893857**	1	1	1			1		1			1	1	1	1		1	1	
**rs2839737**	0.979	1	1			1		1			1	1	1	0.524		1	1	
**rs1561586**	0.959	0.979	1			0.974		1			1	1	0.966	0.519		1	1	
**rs2043953**	0.979	1	1			1		1			1	1	1	0.524		1	1	
**rs2913111**	0.959	0.979	1			0.974		1			1	1	0.966	0.519		1	1	
**rs3012074**	0.979	1	1			1		1			1	1	1	0.524		1	1	

**rs10901851**	1	1	1			1		1			1	1	1	1		1	1	
**rs11245478**	0.966	0.967	1			0.972		1			1	1	0.976	1		1	1	
**rs4962421**	0.966	0.967	1			0.972		1			1	1	0.976	1		1	1	
**rs3781396**	0.912	0.913	0.151			0.927		1			0.151	1	1	0.293		1	1	
**rs2949368**	0.979	1	1			1		1			1	1	1	0.529		1	1	
**rs2937999**	0.979	1	1			1		1			1	1	1	0.529		1	1	
**rs2028395**	0.907	0.908	1			1		1			1	1	1	0.444		1	1	
**rs3781395**	0.966	0.967	1			0.972		1			1	1	0.976	1		1	1	
**rs2289431**	0.912	0.913	0.151			0.927		1			0.151	1	1	0.293		1	1	
**rs11245479**	0.966	0.967	1			0.972		1			1	1	0.976	1		1	1	
**rs11245480**	0.912	0.913	0.151			0.927		1			0.151	1	1	0.293		1	1	
**rs2946993**	0.979	1	1			1		1			1	1	1	0.529		1	1	
**rs10901852**	0.966	0.966	1			0.972		1			1	1	0.976	1		1	1	
**rs4962422**	0.966	0.967	1			0.972		1			1	1	0.976	1		1	1	
**rs4962424**	0.912	0.913	0.151			0.927		1			0.151	1	1	0.293		1	1	
**rs2935653**	0.959	0.979	1			1		1			1	1	1	0.524		1	1	
**rs2936546**	0.979	1	1			1		1			1	1	1	0.537		1	1	
**rs11245481**	0.966	0.967	1			0.972		1			1	1	0.976	1		1	1	
**rs4962724**	0.935	0.935	1			0.946		1			1	1	1	1		1	1	
**rs4962725**	0.861	0.862	0.333			0.686		1			0.333	1	1	0.444		1	1	
**rs10901854**	0.933	0.934	1			0.945		1			1	1	0.976	1		1	1	

	**rs2949371**	0.978	1	1			1		1			1	1	1	0.542		1	1	
	**rs2949372**	0.978	1	1			1		1			1	1	1	0.542		1	1	
	**rs3012065**	0.969	0.969	1			0.487		1			1	1	0.631	1		1	1	
	**rs3012066**	0.969	0.969	1			0.487		1			1	1	0.631	1		1	1	
	**rs2946996**	0.869	0.871	0.248			0.486		1			0.248	1	0.673	0.373		1	1	
	**rs2919290**	0.89	0.891	1			0.718		1			1	1	0.625	0.473		1	1	
	**rs718947**	0.428	0.434	0.165			0.49		0.311			0.165	1	0.69	0.404		1	1	
	**rs718948**	0.472	0.477	0.034			0.515		0.317			0.034	1	0.692	0.698		1	1	

SNPs with a genome-wide significance for the BMI which are located in *CTBP2* and adjacent regions (± 500 kb) (1) were included in the LD analyses. Here, just the LD values (D') for the genome-wide significant SNPs with the here detected variants are shown. No LD structures between the genome-wide SNP is presented.

Ribeye-F

5'- CCCAGAACTGCCTACTCCACCTCGTACACGGTGCCCATGGA**ACTGCTTAAGAGGGAACGCA**GCATGACCGCCTCTCCGC

TGCCCAGCCCGCACGGCAGCCCCCAGCTCCTGCGGAAACCTGGAGCAGCCCCCGTGGAGCCGTCTACCCTGCCACCAGTCAGC

CAAAGTCTCCACACACCTCATTCACCGTACCAGAAGGTGGCCCGGCGGACTGGGGCTCCCATCATCGTGTCAACCATGCTAAC

exon 1 **exon 2**

TCCAGAACCAA | GTATCCGCCCCCAGATCATGAACGGCCCCCTGCACCCCCGCCCCCTGGTGGCACTGCTGGATGGCA**GAGAC**

TGCACTGTGGAGATGCCCATCCTGAAGGACCTGGC**CACTGTGGCCTTCTGTGATG** -3'

Ribeye-1R Ribeye-R

Supplementary Figure 1: Part of the Ribeye mRNA-sequence showing the primer binding sites for the nested PCR.

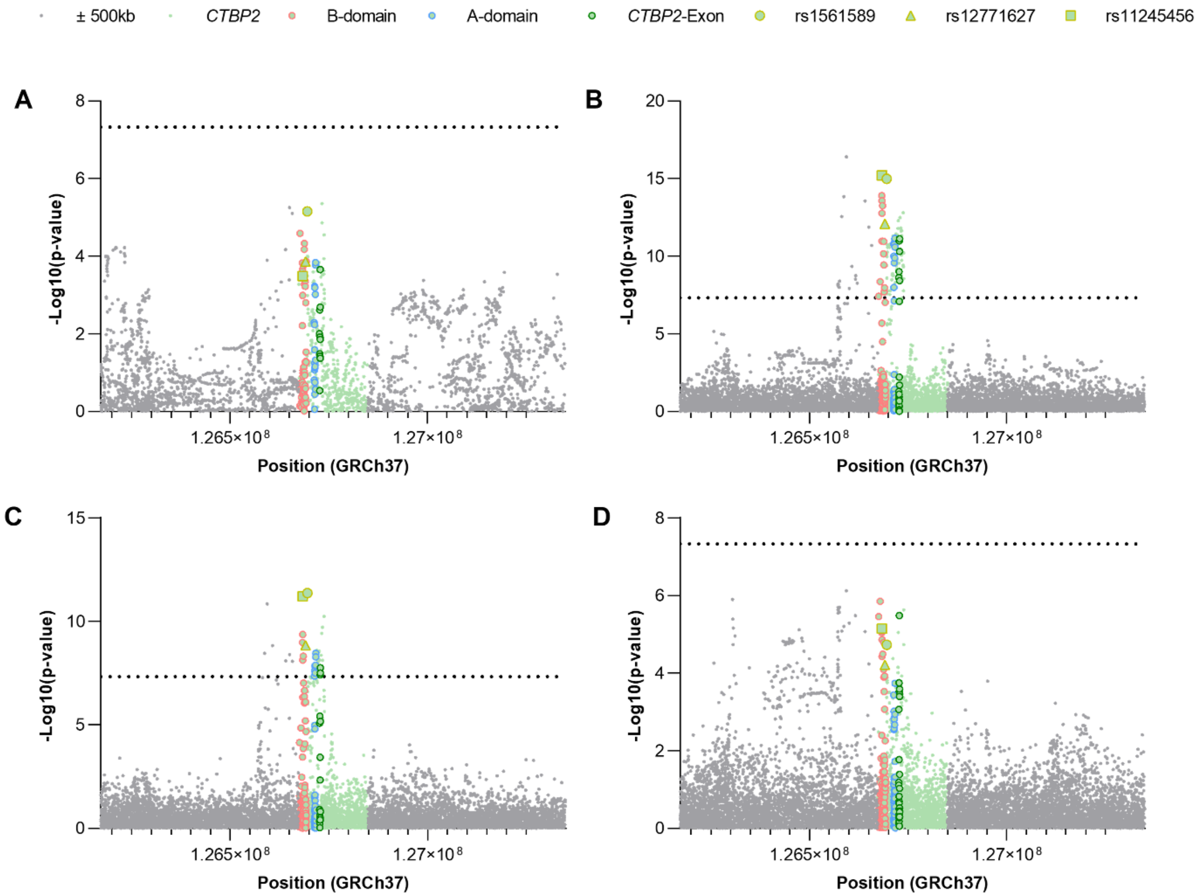

Supplementary Figure 2: Plots of chromosomal location and p values of variants located in CTBP2 and ± 500kb adjacent regions based on BMI and AN GWAS data. Variants were extracted from **A** Watson et al. GWAS for AN (21) and Pulit et al. GWAS for BMI in **B** combined sexes, **C** females and **D** males (22). The −log10(p values) for the association are shown on the y-axis and the chromosomal locations are ordered on the x-axis. Dashed lines depict the genome wide significant threshold p-value < $5*10^{-8}$.

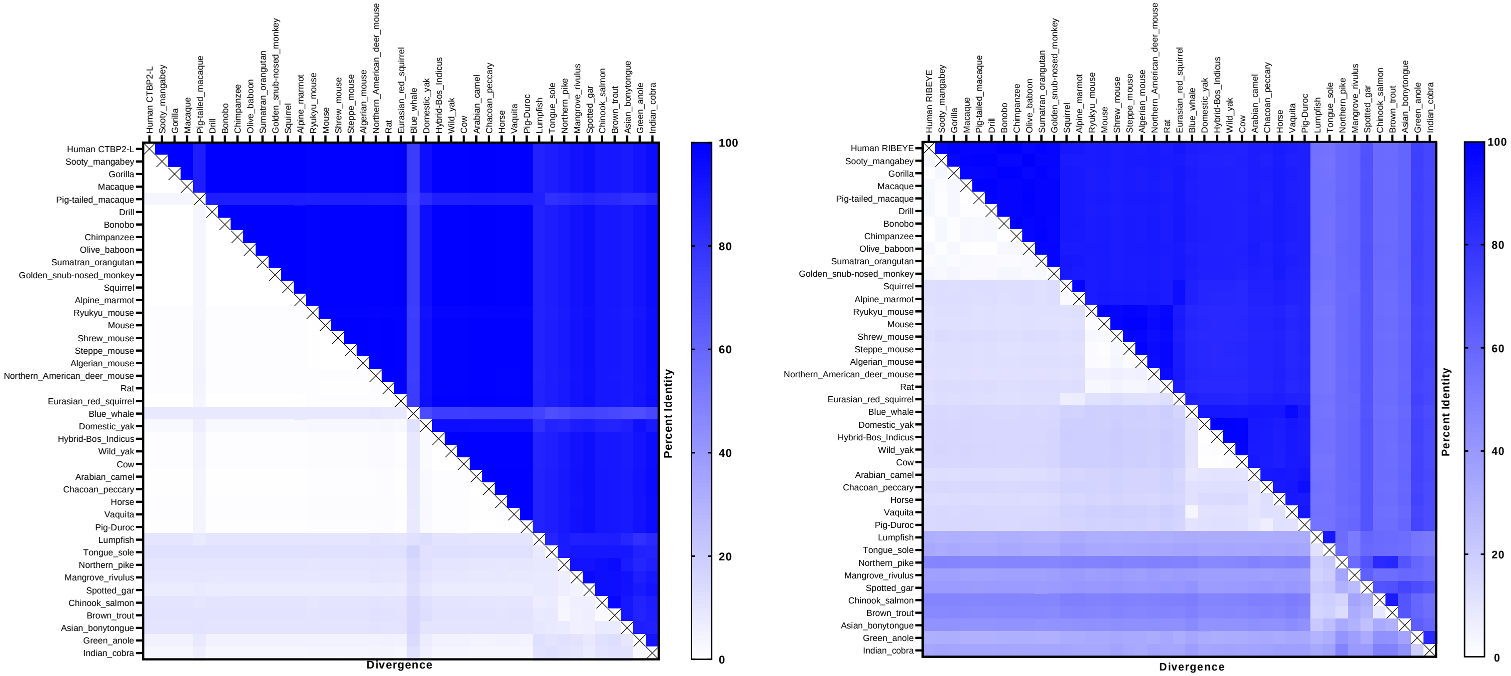

Supplementary Figure 3: Sequence distances between human and 40 species from five superorders in protein level. Left: CTBP2-L; right: RIBEYE.

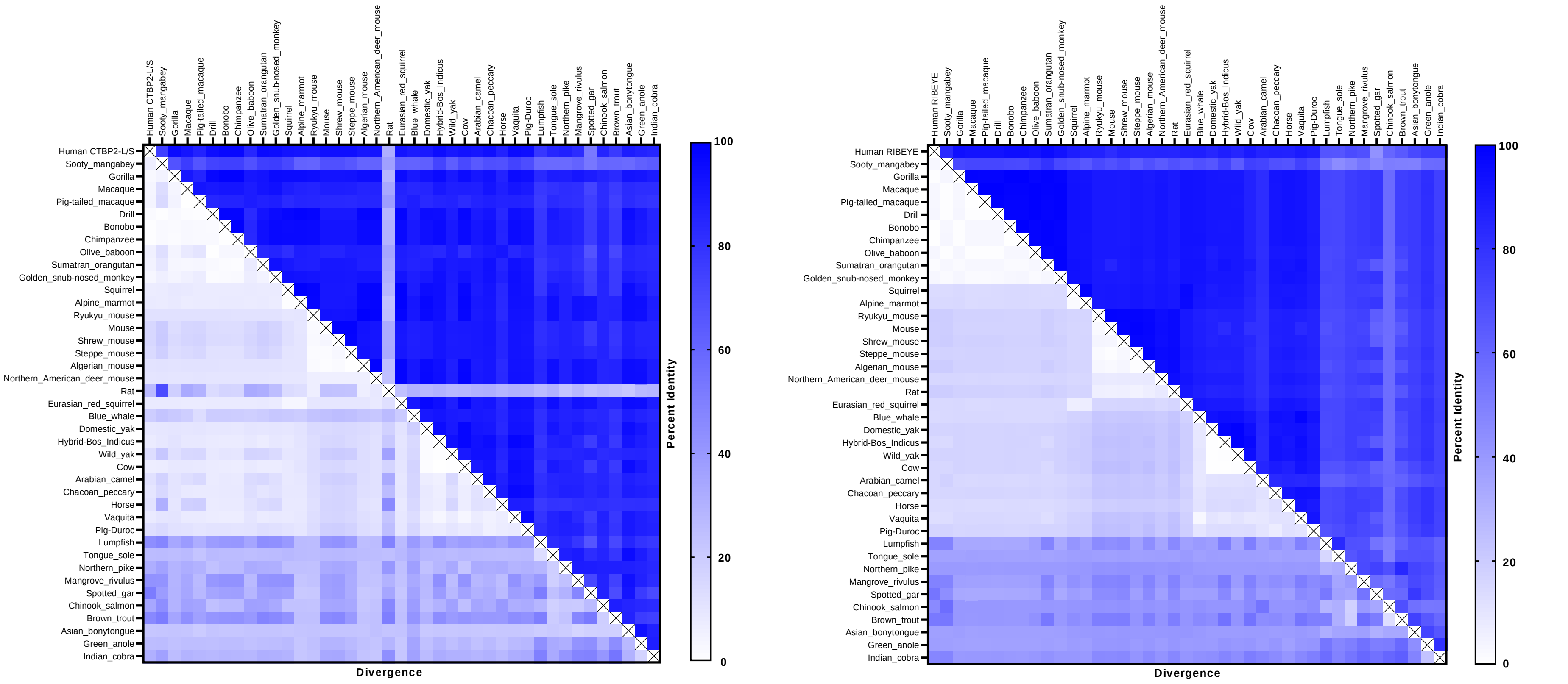

Supplementary Figure 4: Sequence distances between human and 40 species from five superorders in mRNA level. Left: CTBP2-L; right: RIBEYE.

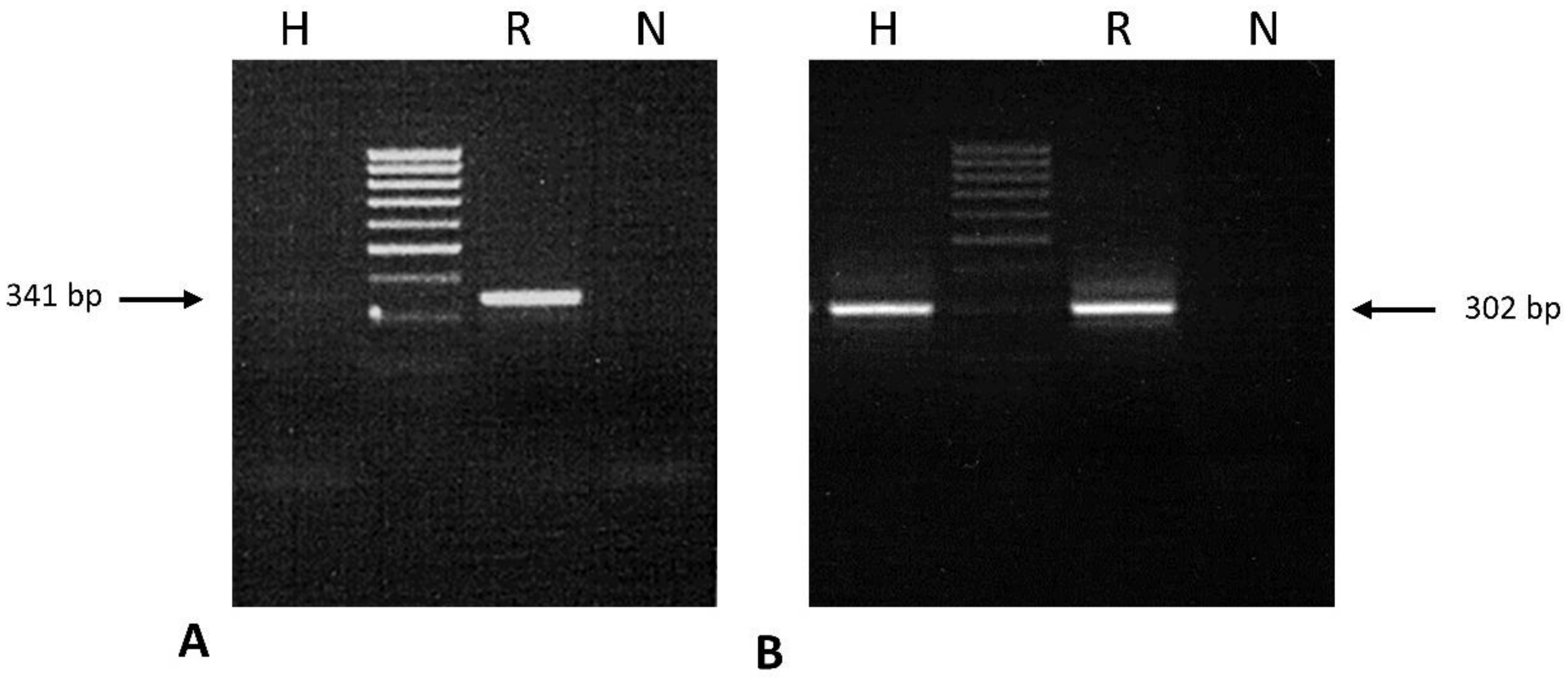

Supplementary Figure 5: Analysis of *Ribeye* expression in murine hypothalamus by two-step PCR. A. Separation of amplified samples with external primers to amplify 341 bp DNA. B. Second PCR amplification using internal primers and first-round PCR product. H = Hypothalamus; R = Retina; N = Negative control. A 100 bp ladder was used as size marker.

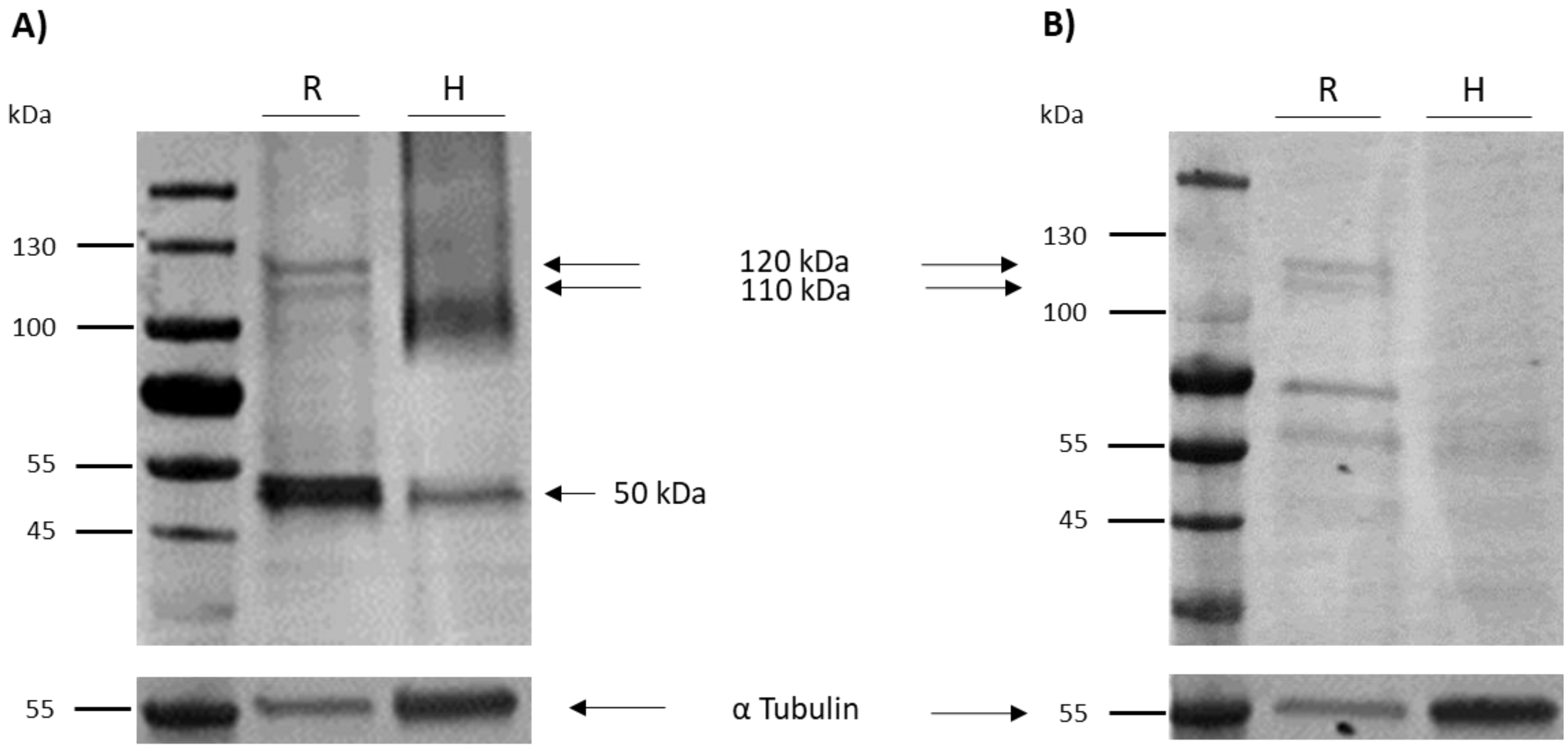

Supplementary Figure 6: Immunoblotting of RIBEYE expression in murine hypothalamus and retina. Mouse hypothalamic (H) and retinal (R) homogenates were used for Western Blot analysis with R4 A: (i)mouse monoclonal anti-CTBP2/RIBEYE B-domain clone 2D9 or R4 B: (ii)mouse monoclonal anti-RIBEYE A-domain antibody clone 12A10, as indicated. The hypothalamus and retina (positive control) of female wildtype C57BL/6J mice (30 µg of total protein for each) were and two different antibodies were utilized (please find the description in Table 1): one (i)against the B-domain (recognize RIBEYE and CTBP2-L/S, Figure R4 A) and (ii)against A-domain (recognize RIBEYE, Figure R4 B). Both antibodies detected two protein bands of ~ 110 and ~ 120 kDa in the retina, both corresponding to RIBEYE. In the hypothalamus none of the RIBEYE-specific protein bands appeared. Also a protein band of ~ 50 kDa, representing CTBP2-L/S is identified by the anti-RIBEYE B-domain antibody. Additional protein bands above and below RIBEYE in hypothalamus-could represent RIBEYE/CTBP2 splice variants or degradation products

References

1. Pulit SL, Stoneman C, Morris AP, Wood AR, Glastonbury CA, Tyrrell J, et al. Meta-analysis of genome-wide association studies for body fat distribution in 694 649 individuals of European ancestry. Hum Mol Genet. 2019;28(1):166-74.
2. Chen S, Francioli LC, Goodrich JK, Collins RL, Kanai M, Wang Q, et al. A genomic mutational constraint map using variation in 76,156 human genomes. Nature. 2024;625(7993):92-100.
3. Watson HJ, Yilmaz Z, Thornton LM, Hubel C, Coleman JRI, Gaspar HA, et al. Genome-wide association study identifies eight risk loci and implicates metabo-psychiatric origins for anorexia nervosa. Nat Genet. 2019;51(8):1207-14.
4. Schwarz JM, Cooper DN, Schuelke M, Seelow D. MutationTaster2: mutation prediction for the deep-sequencing age. Nat Methods. 2014;11(4):361-2.
5. Rentzsch P, Schubach M, Shendure J, Kircher M. CADD-Splice-improving genome-wide variant effect prediction using deep learning-derived splice scores. Genome Med. 2021;13(1):31.
6. Bendl J, Musil M, Stourac J, Zendulka J, Damborsky J, Brezovsky J. PredictSNP2: A Unified Platform for Accurately Evaluating SNP Effects by Exploiting the Different Characteristics of Variants in Distinct Genomic Regions. PLoS Comput Biol. 2016;12(5):e1004962.
7. Adzhubei IA, Schmidt S, Peshkin L, Ramensky VE, Gerasimova A, Bork P, et al. A method and server for predicting damaging missense mutations. Nat Methods. 2010;7(4):248-9.
8. Choi Y, Sims GE, Murphy S, Miller JR, Chan AP. Predicting the functional effect of amino acid substitutions and indels. PLoS One. 2012;7(10):e46688.
9. Choi Y. A fast computation of pairwise sequence alignment scores between a protein and a set of single-locus variants of another protein. Proceedings of the ACM Conference on Bioinformatics, Computational Biology and Biomedicine; Orlando, Florida: Association for Computing Machinery; 2012. p. 414–7.
10. Ng PC, Henikoff S. Predicting deleterious amino acid substitutions. Genome Res. 2001;11(5):863-74.
11. Venselaar H, Te Beek TA, Kuipers RK, Hekkelman ML, Vriend G. Protein structure analysis of mutations causing inheritable diseases. An e-Science approach with life scientist friendly interfaces. BMC Bioinformatics. 2010;11:548.
12. Cheng J, Randall A, Baldi P. Prediction of protein stability changes for single-site mutations using support vector machines. Proteins. 2006;62(4):1125-32.
13. Chen CW, Lin J, Chu YW. iStable: off-the-shelf predictor integration for predicting protein stability changes. BMC Bioinformatics. 2013;14 Suppl 2(Suppl 2):S5.
14. Capriotti E, Fariselli P, Casadio R. I-Mutant2.0: predicting stability changes upon mutation from the protein sequence or structure. Nucleic Acids Res. 2005;33(Web Server issue):W306-10.
15. Gelfman S, Wang Q, McSweeney KM, Ren Z, La Carpia F, Halvorsen M, et al. Annotating pathogenic non-coding variants in genic regions. Nat Commun. 2017;8(1):236.
16. Smith PJ, Zhang C, Wang J, Chew SL, Zhang MQ, Krainer AR. An increased specificity score matrix for the prediction of SF2/ASF-specific exonic splicing enhancers. Human Molecular Genetics. 2006;15(16):2490-508.

17. Cartegni L, Wang J, Zhu Z, Zhang MQ, Krainer AR. ESEfinder: A web resource to identify exonic splicing enhancers. Nucleic Acids Res. 2003;31(13):3568-71.
18. Lim KH, Fairbrother WG. Spliceman--a computational web server that predicts sequence variations in pre-mRNA splicing. Bioinformatics. 2012;28(7):1031-2.
19. Jaganathan K, Kyriazopoulou Panagiotopoulou S, McRae JF, Darbandi SF, Knowles D, Li YI, et al. Predicting Splicing from Primary Sequence with Deep Learning. Cell. 2019;176(3):535-48 e24.
20. Hinney A, Kesselmeier M, Jall S, Volckmar AL, Focker M, Antel J, et al. Evidence for three genetic loci involved in both anorexia nervosa risk and variation of body mass index. Mol Psychiatry. 2017;22(2):321-2.
21. Watson HJ, Yilmaz Z, Thornton LM, Hübel C, Coleman JRI, Gaspar HA, et al. Genome-wide association study identifies eight risk loci and implicates metabo-psychiatric origins for anorexia nervosa. Nature Genetics. 2019;51(8):1207-14.
22. Pulit SL, Stoneman C, Morris AP, Wood AR, Glastonbury CA, Tyrrell J, et al. Meta-analysis of genome-wide association studies for body fat distribution in 694 649 individuals of European ancestry. Human molecular genetics. 2019;28(1):166-74.
